# Synthesis and Ambiphilic Reactivity of Metalated Diorgano‐Phosphonite Boranes

**DOI:** 10.1002/chem.202005437

**Published:** 2021-02-26

**Authors:** Thomas D. Hettich, Richard Rudolf, Christoph M. Feil, Nicholas Birchall, Martin Nieger, Dietrich Gudat

**Affiliations:** ^1^ Institut für Anorganische Chemie University of Stuttgart Pfaffenwaldring 55 70550 Stuttgart Germany; ^2^ Department of Chemistry University of Helsinki P.O Box 55 00014 Helsinki Finland

**Keywords:** alkali metals, ambiphiles, anions, metalation, phosphines

## Abstract

Unprecedented metalated phosphonite boranes were prepared from PH‐substituted precursors and silyl amides. Although potassium derivatives were thermally stable and could even be isolated and structurally characterised, lithiated analogues proved to be unstable towards self‐condensation under cleavage of LiOR at ambient temperature. Reaction studies revealed that the metalated phosphonite boranes exhibit ambiphilic character. Their synthetic potential as nucleophilic building blocks was demonstrated in the synthesis of the first stannylated phosphonite representing a new structural motif in phosphine chemistry.

## Graphical abstract

**Metalation of diorganophosphonite boranes** gave unprecedented O‐substituted phosphide reagents as spectroscopically detectable and even isolable species. The target compounds are ambiphiles that undergo P−O bond functionalisation upon treatment with nucleophiles and act as nucleophilic building blocks towards electrophiles. The latter reactions allow accessing phosphines with unmatched substituent patterns.

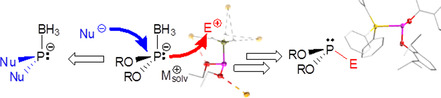

Metathesis of metal phosphides **I** with suitable electrophiles provides beside the complementary reaction of phosphorus‐based electrophiles **II** with nucleophiles a main synthetic avenue to tertiary phosphines **III** (Scheme 1).^[1]^ However, while the “P‐electrophile” route allows accessing a wide variety of products, including specimens with O‐ and N‐based substituents (R=NR′_2_, OR′), the “phosphide approach” is mainly focused on the synthesis of tailored alkyl and aryl phosphines (R=alkyl, aryl), and its application to preparing heteroatom‐functionalized phosphines is clearly underdeveloped. The reason for this bias is a lack of suitable nucleophilic building blocks **I**. Alleviating this deficiency would be certainly desirable and open new routes to organo‐substituted diaminophosphines and phosphonites, which are sought for as tunable ligands for application in catalysis.

**Scheme 1:**
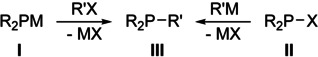
Metathesis routes in phosphine synthesis (X=halide, R′=alkyl, aryl, R=aryl, alkyl, OR′, NR′_2_).

For amino‐derivatives, a change was initiated by Knochel et al.,^[2]^ who first demonstrated the trapping of an elusive transient diaminophosphide borane Li[**1 b**] (A, Scheme 2) by substitution with organic electrophiles (E=alkyl, aryl). Elaborating on this theme, our group introduced metalation of diaminophosphine precursors as alternative route (B, Scheme 2) to a series of nucleophilic building blocks M[**1 a**–**e**] (M=Li, Na, K), and showed that these species are well‐defined reagents that can be characterized spectroscopically and even be isolated.^[3]^

**Scheme 2:**
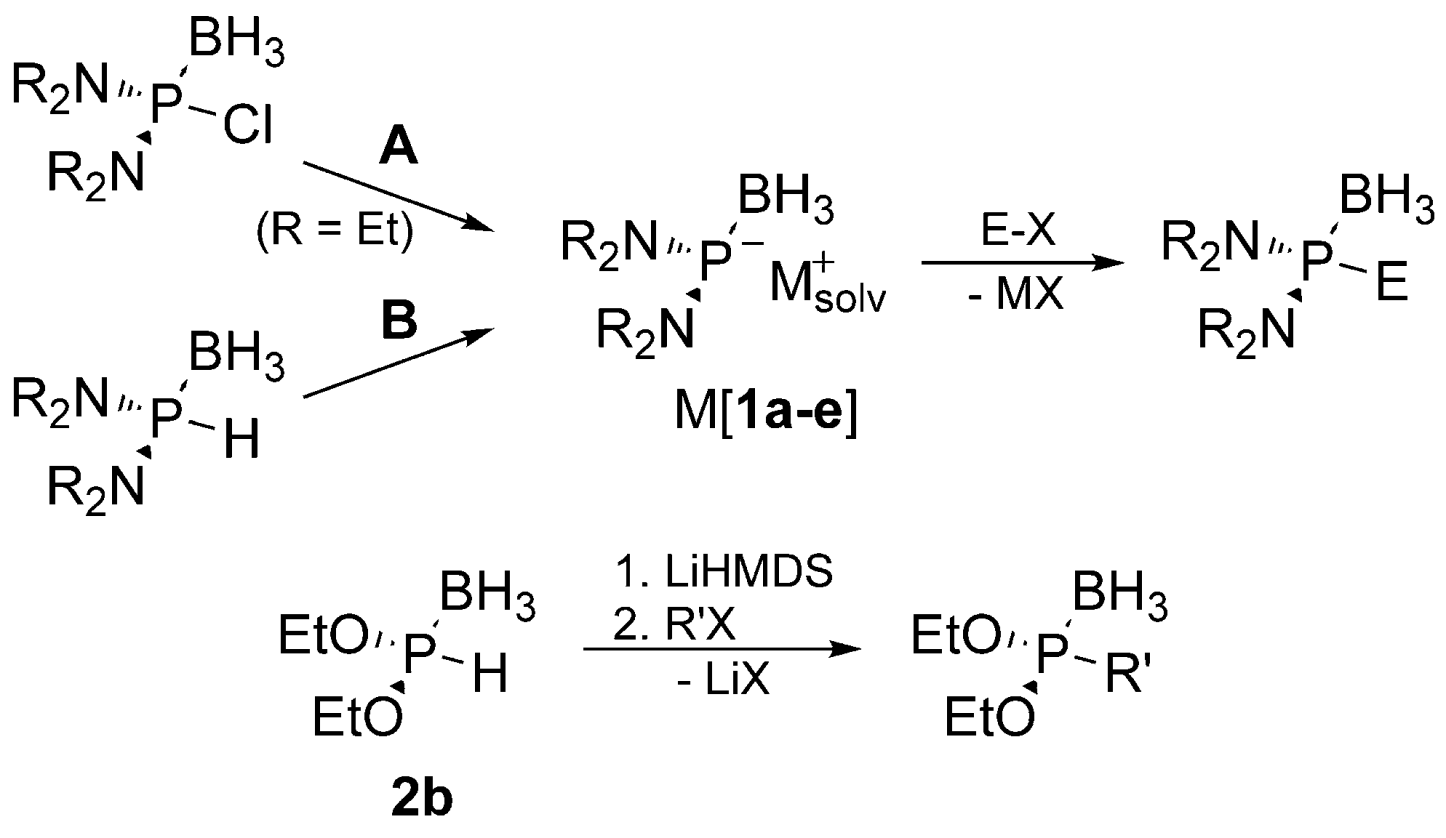
Previously reported phosphine syntheses by metalation of heteroatom substituted secondary phosphine boranes (reaction A: Li, C_10_H_8_ (catalytic); reaction B: MN(SiMe_3_)_2_ or LDA; R=Me (**1 a**), Et (**1 b**), *i*Pr (**1 c**); (R_2_N)_2_=‐N(Ar)CH_2_CH_2_N(Ar)‐ (**1 d**), ‐N(Ar)CHCHN(Ar)‐ (**1 e**); M=Li, K; E=alkyl, aryl, ClSiMe_2_SiMe_2_Cl, (TMEDA)ZnCl, (IDipp)Cu, R′=alkyl).[^2^, ^3^, ^6^]

To the best of our knowledge, P‐metalated diorganophosphonites (i.e., diesters of the elusive phosphonous acid HP(OH)_2_) are not known. The finding that amides react with dialkoxy phosphonites (R′O)_2_PH under displacement of an R′O group rather than deprotonation^[4]^ suggests that, like in diaminophosphines,^[5]^ PH‐metalation is impractical. However, sporadic reports on base‐assisted electrophilic functionalization of borane adducts of dialkyl or mixed alkyl/silylesters of phosphonous acid[^6^, ^7^] led us to speculate that the enhancement of PH‐acidity induced by the borane coordination^[8]^ might suffice to enable selective PH‐metalation in these adducts and generate O‐analogues of M[**1 a**–**e**] as isolable or at least spectroscopically detectable species. In this communication, we present for the first time spectroscopic and structural evidence of metalated phosphonite boranes and their use in the synthesis of new phosphine derivatives. Moreover, reaction studies reveal a unique example of electrophilic behaviour of a phosphide reagent.

Previous reports on dialkyl phosphonite boranes are confined to dimethoxyphosphine borane (**2 a**),^[9]^ which is pyrophoric despite borane protection, and diethoxyphosphine borane **2 b** as well as some sterically encumbered alkyl/silyl derivatives,^[6a]^ respectively. Using the same synthetic protocol, we further prepared new dialkyl and diaryl phosphonite boranes **2 c**,**d** from the respective chlorophosphites and LiBH_4_ (Scheme 3). Alkylated **2 b**,**c** were purified by aqueous work up; **2 d** decomposes when exposed to excess water, but could be isolated under anhydrous conditions as a crystalline solid (see Supporting Information for details). Treatment of **2 b**–**d** with potassium hexamethyldisilazide (KHMDS) furnished hexamethyldisilazane along with new phosphorus‐containing species identified as the expected metalation products K[**3 b**–**d**] by spectroscopic data and chemical trapping (see below).

**Scheme 3:**
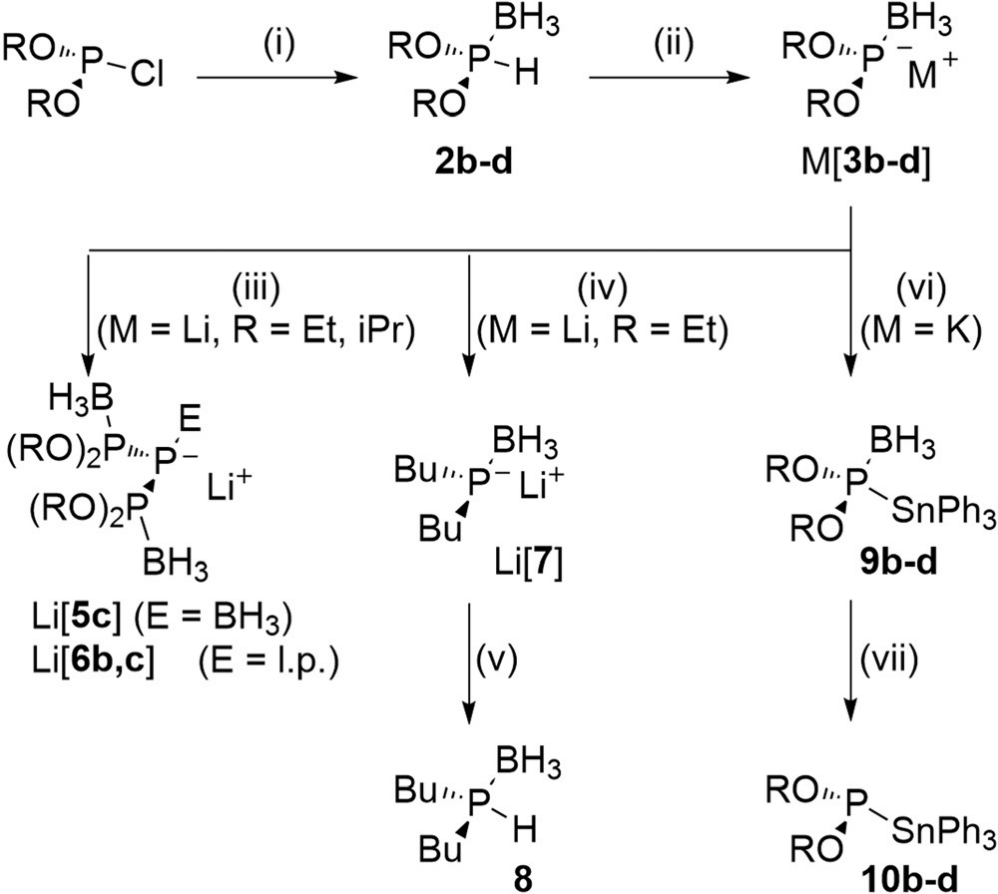
Synthesis and reactivity of metalated phosphonite boranes. Reagents and conditions: (i) LiBH_4_, THF, −78 °C to rt. (ii) KHMDS in THF (K[**3 b**,**c**]) or toluene, −78 °C to rt (K[**3 d**]), or LiHMDS, THF, −78 °C (Li[**3 b**,**c**]). (iii) −78 °C to rt. (iv) excess BuLi, THF, −78 °C. (v) MeOH, THF, −78 °C to rt. (vi) Ph_3_SnCl, THF or toluene, rt. (vii) excess Et_3_N, toluene, rt (**10 d**) or DABCO, C_6_D_6_, 50 °C (**10 b**,**c**). R=Et (**b**), *i*Pr (**c**), 2,6‐diisopropylphenyl (**d**).

The dialkyl derivatives K[**3 b**] and K[**3 c**] could be precipitated from the reaction mixture and were isolated as colourless, air and moisture sensitive solids. Even if these materials proved unsuitable for XRD studies, we serendipitously succeeded in obtaining a single‐crystal from a reaction of **2 b** with an excess of KHMDS. The crystal structure reveals the presence of a double salt containing equal amounts of K[**3 b**] and KHMDS (Figure 1). Both types of anions and two crystallographically independent cations connect in an alternating sequence via K ⋅⋅N and K ⋅⋅O contacts to form one‐dimensional arrays aligned parallel to the crystallographic b‐axis. Additional BH⋅⋅⋅K agostic interactions enforce the ion stacking in each array and induce pairing of two arrays to form ribbon‐like superstructures by cross‐linking each cation with a borane unit in the other ion stack (Figure S1). The coordination sphere of the cations is filled up by an additional THF ligand and the formation of secondary contacts to Me_3_Si‐groups. Altogether, the amide anions act as μ_2_‐κ*N*:κ*N*‐bridging ligands to two metals, whereas the phosphide borane units bind predominantly via their BH_3_ units and oxygen atoms to four metals. The shortest metal‐phosphorus contacts (K ⋅⋅P 3.528(1) to 3.788(1) Å), albeit well below the sum of van‐der‐Waals radii (4.63 Å),^[10]^ exceed those in K[(Me_2_N)_2_P(BH_3_)] (K[**1 a**]) (K‐P 3.301(1), P2‐K2 3.352(1) Å)^[3b]^ and lack the specific orientation implied by a direct metal‐ligand interaction. We view these features as indication that the dialkoxyphosphide borane anion in K[**3 b**] shows, in contrast to amino‐substituted **1 a^−^**,^[3b]^ no strong inclination for P‐coordination and the metal‐ligand interaction is governed by electrostatic attraction between the metal ion and the *N*‐ and O‐atoms.

**Figure 1:**
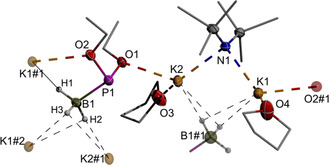
Representation of the molecular structures of the cations and anions in crystalline [(KHDMS)(K[**3 b**])(THF)_2_]. Thermal ellipsoids are drawn at the 50 % probability level. For clarity, carbon atoms are drawn using a wire model, hydrogen atoms except those in BH_3_ units are omitted, and only one orientation of disordered fragments (one SiMe_3_ group and one THF) is shown. Atoms labelled #1 are parts of adjacent formula units. Selected distances [Å]: P1−O1 1.6540(13), P1−O2 1.6619(13), P1−B1 1.926(2), K1−O2 2.7237(13), K1−O4 2.7396(17), K1−N1 2.7959(15), K2−O3 2.7049(15), K2−O1 2.7456(13), K2−N1 2.8189(15), B1−H1 1.11(2), B1−H2 1.13(2), B1−H3 1.13(2), K1−H3#1 2.78(2), K1−H2#1 2.90(2), K2−H2#1 2.81(2), K2−H3#1 2.86(2), K1#1−H1 2.80(2).

The ^31^P NMR signals of K[**3 b**–**d**] still show the splitting arising from spin coupling between the ^31^P and ^11^B (I=3/2) nuclei, but lack the doublet structure caused by ^1^ *J*_PH_ coupling across a P−H bond, thus confirming that deprotonation was successful. Comparison of the ^31^P and ^11^B NMR data of K[**3 b**–**d**] with those of precursors **2 b**–**d** (Table 1) shows that the metalation induces a modest rise in δ^11^B along with a stark increase of some 170 ppm in δ^31^P and a decrease in the magnitude of ^1^ *J*_P11B_. The same trends hold for diaminophosphide boranes.^[3]^

**Table 1:** ^31^P and ^11^B NMR chemical shifts [ppm] and ^1^ *J*_P11B_ coupling constants [Hz] for **2 b**–**d** and M[**3 b**–**d**] (M=Li, K).

	**2 b** ^[a]^	M[**3 b**]^[b,c]^	**2 c** ^[a]^	M[**2 c**]^[b,c]^	**2 d** ^[d]^	K[**2 d**]^[e]^
*δ* ^31^P	127.3	303.5 [294.5]	119.3	290.9 [287.8]	140.8	325.8
*δ* ^11^B	−42.8	−27.4 [−33.5]	41.3	−30.3 [−32.8]	−39.9	−29.6
^1^ *J*_P11B_	72	37 [26]	76	36. [31]	52.	27.

[a] In CDCl_3_. [b] In [D_8_]THF. [c] For M=K [M=Li]. [d] In C_6_D_6_. [e] In [D_8_]toluene.

The increase in δ^31^P is counter intuitive when compared to the negative metalation shifts of alkyl and aryl phosphine boranes,^[3]^ but prevails as well in phosphinidenoid complexes Li_solv_[(Me_3_Si)_2_CH‐P(X){M(CO)_5_}] (X=halide, M=Cr, Mo, W) featuring a single electronegative halide substituent on phosphorus.^[11]^ Preliminary DFT studies relate the deshielding in **3 b^−^** relative to **2 b** to a strong increase in the paramagnetic shielding term that is as in cyclic diaminophosphines^[12]^ dominated by a large contribution from the lone pair at phosphorus (Tables S2, S3). The origin of this effect can be traced to a marked decline of the HOMO–LUMO gap, which is an important precondition for amphiphilic behaviour.

Treatment of **2 b**,**c** with LiHMDS furnished products that were assigned as Li[**3 b**,**c**] but proved unstable at ambient temperature (see below) and were only characterized spectroscopically. The observation of a metal influence on ^31^P and ^11^B NMR chemical shifts (Table 1) suggests the presence of contact ion pairs. This conjecture was confirmed by a ^1^H,^7^Li HOESY spectrum of Li[**3 b**] (see Supporting Information) showing cross peaks between the signals of the metal ion and the EtO groups. The DOSY spectrum of a mixture containing Li[**3 b**] and (EtO)_3_P(BH_3_) (**4**) recorded at −70 °C (Figure S20) revealed similar diffusion coefficients for both species (*D*(Li[**3 b**])/*D*(**4**)=0.8), and led us to formulate Li[**3 b**] like its amino‐substituted analogues Li[(**1 a**–**e**)]^[3]^ as a monomer.

At ambient temperature, Li[**3 c**] decays in solution to furnish the triphosphide tris‐borane adduct Li[**5 c**] (Scheme 3) identified by a single‐crystal XRD study (Figure 2). Further reaction with triethyl amine proceeded surprisingly under deprotection of the central, negatively charged phosphorus atom (rather than the formally neutral terminal phosphorus atoms) to afford a mixture of Et_3_NBH_3_ and bis‐borane complex Li[**6 c**]. Transient phosphide Li[**3 b**] undergoes a similar condensation to furnish directly bis‐borane adduct Li[**6 b**]. Formation of LiOEt as a by‐product was established by ^1^H NMR spectroscopy, and the constitution of Li[**6 b**] confirmed by a single‐crystal XRD study (Figure 2).

**Figure 2:**
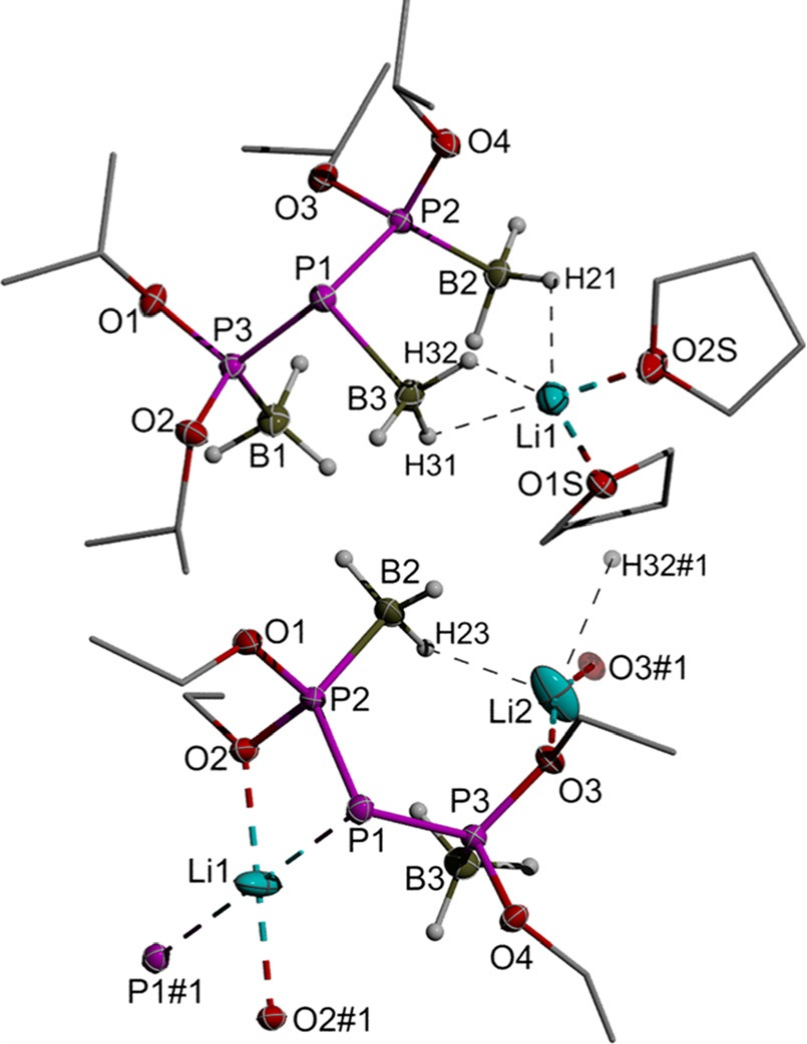
Representations of the molecular structure of (THF)_2_Li[**5 c**] (top) and the anion and adjacent cations (occupancy 0.5) in crystalline Li[**6 b**] (bottom). Thermal ellipsoids are drawn at the 50 % probability level. For clarity, carbon atoms are drawn using a wire model, hydrogen atoms except those in BH_3_ units are omitted, and only one of two disordered positions for Li2 in Li[**6 b**] is shown (see cif files for details of the disorder). Atoms labelled #1 are parts of adjacent formula units. Selected distances [Å]: (THF)_2_Li[**5 c**]: P1−P2 2.173(5), P1−P3 2.1822(5), P1−B3 1.993(2), P2−O3 1.594(1), P2−O4 1.598(1), P2−B2 1.910(2), P3−O1 1.586(1), P3−O2 1.602(1), P3−B1 1.902(2), Li1−O2S 1.927(3), Li1−O1S 1.945(3), Li1−H21 1.96(2), Li1−H31 1.97(2), Li1−H32 2.15(2); Li[**6 b**]: P1−P2 2.1361(4), P1−P3 2.1206(4), P1−Li1 2.7907(4), P2−O1 1.594(1), P2−O2 1.630(1), P2−B2 1.909(2), P3−O4 1.598(1), P3−O3 1.621(1), P3−B3 1.908(2), O2−Li1 2.001(1), O3−Li2 1.97(1), Li2−H23 2.04(2), Li2−H32#1 2.32(2).

The molecular structure of Li(THF)_2_[**5 c**] consists of isolated ion pairs. The lithium ion binds to two of the borane units and two THF ligands; there is no Li‐P contact. Preferred coordination of alkali metal ions to the BH_3_ unit of alkyl/aryl‐phosphide boranes had previously been observed both in the crystalline state^[13]^ and in solution.^[14]^ The P‐B distance in the central phosphide unit (P1‐B3 1.993(2) Å) exceeds those in the neutral phosphine units (P3‐B1 1.902(2), P2‐B2 1.910(2) Å). A similar lengthening is also found in metalated aryl/alkyl‐phosphide boranes^[15]^ as well as M[**1 a**] (M=Na, K)^[3b]^ and K[**3 b**], but the P‐B distance in Li[**5 c**] is beyond the previously known range of 1.94 to 1.97 Å.[^3b^, ^13^, ^15^] This exceptional lengthening seems well suitable to explain both the easy displacement of the borane unit as well as the absence of significant line broadening or splitting arising from ^31^P,^11^B spin coupling in the ^31^P NMR spectrum.

Crystalline Li[**6 b**] is composed of coordination polymeric arrays of μ_2_‐bridging anions and bare lithium cations occupying two crystallographically distinguishable sites. Both metal ions feature contacts to oxygen atoms of two EtO‐moieties (Li2‐O3 1.93(1), Li1‐O2 2.001(1) Å) from different anions, and the coordination spheres are completed by contacts to the central phosphorus atoms of two triphosphides (Li1, Li1‐P1 2.7907(4) Å) or agostic interactions with B−H bonds of two BH_3_ units (Li2). Very short P‐P bonds (2.1361(4) and 2.1206(4) Å) imply that the negative charge of the triphosphide is stabilized by hyperconjugation.

The P−P bond formation processes leading to Li[**5 b**,**c**] and Li[**6 b**] may in principle follow either an associative route characterized by nucleophilic substitution of the alkoxy groups in one molecule Li[**3 b**,**c**] by two more phosphides, or a dissociative pathway initiated by α‐elimination of lithium alkoxide to yield transient phosphinidenes which would then undergo intermolecular P−O bond insertion. Since we detected neither the formation of cyclic oligophosphines as by‐products nor succeeded in trapping a phosphinidene by cycloaddition with 2,3‐dimethyl‐1,3‐butadiene, which are considered typical signatures of reactions involving transient phosphinidenes,^[17]^ we consider the dissociative route unlikely. On the other hand, the susceptibility of the phosphorus atom in Li[**3 b**] towards nucleophilic attack is corroborated by its instantaneous reaction with an excess of *n*BuLi at −50 °C to yield a spectroscopically detectable product (δ^31^P −72.1 ppm, ^1^ *J*_P11B_=37 Hz) that was cleanly converted into known phosphine borane **8** ^[18]^ upon quenching the reaction with MeOH, and is therefore assigned as dibutylphosphide borane Li[**7**]^[19]^ (Scheme 3). To the best of our knowledge, this transformation of Li[**3 b**] represents the first clear example for a phosphide derivative acting as an electrophile.

We further tested the application of K[**3 b**–**d**] as nucleophilic building blocks in reactions with Ph_3_SnCl. Metathesis to afford the expected products **9 b**–**d** occurred with equal selectivity in THF and toluene solution. The reaction in THF was faster due to superior solubility of the phosphide, but using toluene facilitated work‐up and isolating pure products after crystallization from hexane. The ^31^P chemical shifts of **9 b**–**d** (178 to 198 ppm) are intermediate between those of phosphine boranes **2 b**–**d** and the respective phosphides K[**3 b**–**d**], and ^31^P,^119^Sn coupling constants (77–384 Hz) match those in the few neutral stannylphosphine boranes known (80–308 Hz).^[20]^ The molecular structures of **9 b**–**d** (see Figures 3 and S3, S4) show no anomalies apart from a distortion of the tetrahedral coordination at phosphorus that is manifested in a widening of one and contraction of the other Sn‐P‐O angle and arises presumably from steric interactions between the bulky Ph_3_Sn‐ and alkoxy‐/phenoxy groups.

**Figure 3:**
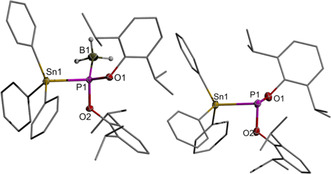
Representations of the molecular structure of **9 d** (left) and **10 d** (right) in the crystal. Thermal ellipsoids are drawn at the 50 % probability level. For clarity, carbon atoms are drawn using a wire model, hydrogen atoms except those in BH_3_ units are omitted, and only one position of the disordered *i*Pr groups (on one of the Dipp substituents) is shown. Selected distances [Å] and angles [°]: **9 d**: Sn1−P1 2.5334(3), P1−O2 1.620(1), P1−O1 1.622(1), P1−B1 1.880(2), O2‐P1‐O1 103.50(5), O2‐P1‐Sn1 93.17(3), O1‐P1‐Sn1 113.68(3); **10 d**: Sn1−P1 2.571(1), P1−O2 1.662(3), P1−O1 1.667(2), O2‐P1‐O1 102.05(13), O2‐P1‐Sn1 85.60(9), O1‐P1‐Sn1 100.46(9).

To evaluate the possibility of removing the borane unit, we studied reactions of **9 b**–**d** with amines.^[21]^ Treatment of **9 d** with excess NEt_3_ in toluene at room temperature resulted in the clean formation of Et_3_NBH_3_ and stannyl phosphonite **10 d**, which was isolated after work‐up as colourless crystals. Deprotection of **9 b**,**c** required more forcing conditions but could be achieved by reaction with DABCO at 50 °C. Reaction monitoring by ^31^P NMR spectroscopy (Figure S60) revealed that within 90 min ca. 93 % of phosphine borane **9 b** was converted into a mixture of two species assigned as tetraethoxy diphosphine^[22]^ and stannyl phosphonite **10 b** (22 % and 71 % by integration of NMR signals). Similar results were obtained for **9 c**. The detection of the signal of Ph_6_Sn_2_ ^[23]^ in the ^119^Sn NMR spectra of both reaction mixtures suggests that the diphosphines arise from dismutation of **9 b**,**c**. That prolonged heating affected both further consumption of the residual phosphine borane and increased diphosphine formation is in line with this hypothesis.

Comparing the molecular structure of **10 d** (Figure 3) with that of **9 d** reveals that loss of the borane induces lengthening of the P−O and P−Sn distances and an increased pyramidalization (sum of O−P−O/Sn angles 288.1(3)° vs. 310.4(1)° in **9 d**), while the pronounced asymmetry of O−P−Sn angles is conserved. The changes are in line with the expectation that deprotection is accompanied by rehybridisation enhancing the s‐character of the lone pair at phosphorus. The ^31^P NMR chemical shifts of **10 b**–**d** (231 to 273 ppm) are not as large as in alkali metal derivatives M[**3 b**–**d**] but clearly exceed those in phosphonites and even phosphites bearing three electronegative substituents. Our preliminary DFT studies allow relating this effect as well to a decrease in the HOMO–LUMO gap (Table S2) and suggest thus that **10 b**–**d** may likewise show ambiphilic reactivity.

In summary, we provided the first spectroscopic and structural proof for P‐metalated phosphonite boranes and demonstrated that these species are ambiphiles that can act both as nucleophilic building blocks and electrophiles. We also showed the feasibility of cleaving the borane protecting group to give unprecedented free stannyl phosphonites. Synthetic scope and ligand behaviour of the new ambiphilic reagents, as well as the possible involvement of phosphinidenes in triphosphide formation are currently under research and will be reported in forthcoming studies.

## Conflict of interest

The authors declare no conflict of interest.

## Supporting information

As a service to our authors and readers, this journal provides supporting information supplied by the authors. Such materials are peer reviewed and may be re‐organized for online delivery, but are not copy‐edited or typeset. Technical support issues arising from supporting information (other than missing files) should be addressed to the authors.

Supplementary
