# Supplementary material for: Synthesis and Ambiphilic Reactivity of Metalated Diorgano‐Phosphonite Boranes
Source: Chemistry. 2021 Feb 26;27(17):5412–6. doi: 10.1002/chem.202005437 (PMC8048877; doi:10.1002/chem.202005437)
Supplement: Supplementary file 1 — Supplementary [file CHEM-27-5412-s001.pdf]

Chemistry–A European Journal

Supporting Information

Synthesis and Ambiphilic Reactivity of Metalated Diorgano-Phosphonite Boranes

Thomas D. Hettich,[a] Richard Rudolf,[a] Christoph M. Feil,[a] Nicholas Birchall,[a]
Martin Nieger,[b] and Dietrich Gudat*[a]

EXPERIMENTAL PROCEDURES 2
CRYSTALLOGRAPHIC STUDIES 4
NMR-SPECTRA 9
IR-SPECTRA 41
MASS SPECTRA 46
COMPUTATIONAL STUDIES 46
REFERENCES 48

Experimental Procedures

If not stated otherwise, all manipulations were performed in flame-dried glassware under inert conditions using purified argon. Chlorophosphites and diethoxyphosphine borane were prepared as described elsewhere.[1]-[4] $LiBH_4$, metal hexamethyldisilazides (MHMDS, M = Li, K), Ph_3SnCl and 1,4-diazabicyclo(2.2.2)octane (DABCO) were purchased from Sigma Aldrich and stored under inert atmosphere. DABCO was sublimed prior to use. NMR spectra were recorded on Bruker Avance 250 (^{1}H: 250.0 MHz, ^{7}Li: 97.2 MHz, ^{11}B: 80.2 MHz, ^{13}C: 62.9 MHz, ^{31}P: 101.2 MHz, ^{119}Sn: 93.2 MHz) or Bruker Avance 400 (^{1}H: 400.1 MHz, ^{7}Li: 155.4 MHz, ^{11}B: 128.4 MHz, ^{13}C: 100.5 MHz, ^{31}P: 161.9 MHz, ^{119}Sn: 149.2 MHz,) NMR spectrometers at 293 K if not stated otherwise. ^{1}H Chemical shifts were referenced to TMS using the signals of the residual protons of the deuterated solvent (δ^1H = 7.27 ($CDCl_3$), 7.15 (C_6D_6), 2.09 (toluene-d_8), 1.73 (THF-D_8)) as secondary reference. Spectra of heteronuclei were referenced using the Ξ-scale[5] employing 85 % H_3PO_4 (Ξ = 40.480747 MHz, ^{31}P), $SnMe_4$ (Ξ = 37.290655 MHz, ^{119}Sn), $BF_3{\cdot}OEt_2$ (Ξ = 32.083974 MHz, ^{11}B) and LiCl (Ξ = 38.863797, ^{7}Li) as secondary reference, respectively. ^{119}Sn NMR spectra were recorded using the DEPT pulse sequence. Coupling constants involving boron and tin nuclei refer to the isotopes ^{11}B and ^{119}Sn if not stated otherwise and prefixes *i*-, *o*-, *m*-, *p*- denote the atomic positions in aromatic rings. FTIR spectra were recorded with a Thermo Scientific/Nicolet iS5 instrument equipped with an iD5 ATR accessory. Mass spectra were obtained with a Bruker Daltonics Microtof-Q mass spectrometer. Given masses refer to the peak representing the most abundant isotope combination. Elemental analyses were performed with an Elementar Micro Cube elemental analyser. The high chemical sensitivity of metalated phosphides precluded obtaining satisfactory elemental analyses in these cases.

Di(isopropoxy)phosphine borane (2c)

A solution of chlorodiisopropylphosphite (8.24 g, 4.46 mmol) in THF (80 mL) was cooled to -78 °C. Solid $LiBH_4$ (2.90 g, 4.46 mmol) was added. The mixture was stirred for 15 minutes at -78 °C and then for 1 h at room temperature. After re-cooling to -78 °C, water (100 mL) was slowly added. The product was extracted with EtOAc (2 x 50 mL), and the combined organic phases were dried over Na_2SO_4. Purification by column chromatography (petroleum ether/EtOAc 8:2) afforded the product as colourless oil (3.36 g, 20.5 mmol, 46 %). – ^{1}H NMR ($CDCl_3$): δ = 6.99 (d, $^1J_{PH}$ = 445 Hz, 1 H, PH), 4.58 (dsept, $^3J_{PH}$ = 9.2 Hz, $^3J_{HH}$ = 6.2 Hz, 2 H, OCH), 1.33 (d, $^3J_{HH}$ = 6.2 Hz, 12 H, CH_3), 0.57 (broad dq, $^1J_{BH}$ = 96 Hz, $^2J_{PH}$ = 13 Hz, 3 H, BH_3). – ^{31}P NMR ($CDCl_3$): δ = 119.3 (broad dq, $^1J_{PH}$ = 444 Hz, $^1J_{PB}$ = 76 Hz). ^{11}B NMR ($CDCl_3$): δ = 41.3 (dq, $^1J_{PB}$ = 76 Hz, $^1J_{BH}$ = 96 Hz). – ^{13}C{^{1}H} NMR ($CDCl_3$): δ = 73.8 (d, $^2J_{CP}$ = 7 Hz, CH), 24.0 (d, $^3J_{CP}$ = 4 Hz, CH_3), 23.7 (d, $^3J_{CP}$ = 3 Hz, CH_3). – IR (cm^{-1}): ṽ = 2393, 2346 (νPH, νBH). – (+)ESI-MS: m/z = 187.1028 (MNa^+, calcd. 187.1031). – ($C_6H_{18}BO_2P$ (163.99 g mol^{-1}): calcd. C 43.94 H 11.06, found C 43.66 H 10.85.

Bis(2,6-diisopropylphenoxy)phosphine borane (2d)

A solution of chloro-bis(2,6-diisopropylphenoxy)phosphine (4.18 g, 10.7 mmol) in THF (30 mL) was cooled to -78 °C. $LiBH_4$ (4 M solution in THF, 2.7 mL, 10.7 mmol) was added. The resulting colourless solution was warmed up to room temperature and CH_2Cl_2 (3 mL) was added. After stirring for 5 minutes, all volatiles were removed under reduced pressure. The residue was dissolved in hexane (20 mL) and filtered over dry silica. The separated solids were washed with hexane (3 x 20 mL). The volume of the filtrate was reduced to approx. 5 mL and stored at -25 °C to afford colourless crystals (1.533 g, 3.830 mmol, 36 %). – ^{1}H NMR (C_6D_6): δ = 7.37 (dm, $^1J_{PH}$ = 423 Hz, 1 H, PH), 7.03-6.98 (m, 6 H, aryl-H), 3.40 (sept, $^3J_{HH}$ = 6.0 Hz, 4 H, CH), 1.15 (d, $^3J_{HH}$ = 6.7 Hz, 12 H, CH_3), 1.14 (d, $^3J_{HH}$ = 6.7 Hz, 12 H, CH_3). – ^{31}P NMR (C_6D_6): δ = 140.8 (dm, $^1J_{PH}$ = 423 Hz). – ^{11}B NMR (C_6D_6): δ = -39.9 (dq, $^1J_{PB}$ = 52 Hz, $^1J_{BH}$ = 76 Hz). – ^{13}C{^{1}H} NMR (C_6D_6): δ = 148.4 (d, $^2J_{PC}$ = 11 Hz, *i*-C), 140.2 (d, $^3J_{PC}$ = 3 Hz, *o*-C), 126.4 (d, $^5J_{PC}$ = 2 Hz, *p*-C), 124.5 (d, $^4J_{PC}$ = 2 Hz, *m*-C), 27.5 (s, CH), 23.5 (s, CH_3), 23.1 (s, CH_3). – IR (cm^{-1}): ṽ = 2420 (νPH), 2389, 2344 (νBH). – (+)ESI-MS: m/z 423.2581 (MNa^+, calcd. 423.2599). – $C_{24}H_{38}BO_2P$ (400.35 g mol^{-1}): calcd. C 72.00 H 9.57, found C 71.70 H 9.50.

Potassium diethoxyphosphide borane (K[3b])

KHMDS (4.285 g, 21.48 mmol) was dissolved in hexane/Et_2O (80 mL/10 mL). Diethoxyphosphine borane **2b** (2.920 g, 21.48 mmol) was added dropwise, resulting in precipitation of the phosphide. The suspension was warmed to room temperature and filtered. The precipitate was washed with hexane (50 mL) and dried in vacuum to afford K[**3b**] as colourless solid (2.417 g, 13.89 mmol, 65 %). ^{1}H NMR (THF-d_8): δ = 3.95-3.70 (m, 4 H, OCH_2), 1.19 (t, $^3J_{HH}$ = 7.0 Hz, 6 H, CH_3), 0.29 (q, $^1J_{BH}$ = 88 Hz, 3 H, BH_3). – ^{31}P{^{1}H} NMR (THF-d_8): δ = 302.3 (q, $^1J_{PB}$ = 37 Hz). – ^{11}B{^{1}H} NMR (THF-d_8): δ = -27.4 (d, $^1J_{PB}$ = 37 Hz). – IR (cm^{-1}): ṽ = 2394, 2340(sh) (νBH).

Potassium diisopropoxyphosphide borane (K[3c])

The synthesis was performed as described for K[**3b**] using KHMDS (4.087g, 20.49 mmol) and **2c** (3.360, 20.49 mmol) in hexane/Et_2O (80 mL/10 mL). Colourless solid (2.303 g, 11.96 mmol, 55%). – ^{1}H NMR (THF-d_8): δ = 3.99 (dsept., $^3J_{PH}$ = 8.2 Hz, $^3J_{HH}$ = 6.2 Hz, 2 H, OCH, 1.19 (d, $^3J_{HH}$ = 6.0 Hz, 6 H, CH_3), 1.12 (d, $^3J_{HH}$ = 6.0 Hz, CH_3), 0.32 (q, $^1J_{BH}$ = 88 Hz, 3 H, BH_3). – ^{31}P{^{1}H} NMR (THF-d_8): δ = 290.9 (b). – ^{11}B{^{1}H} NMR (THF-d_8): δ = -30.3 (d, $^1J_{PB}$ = 36 Hz). – ^{13}C{^{1}H} NMR (THF-d_8): δ = 72.9 (b, OCH), 25.5 (s, CH_3), 25.4 (s, CH_3). – IR (cm^{-1}): ṽ = 2325, 2279 (νBH).

In situ preparation of potassium bis(2,6-diisopropylphenoxy)phosphide borane (K[3d])

A NMR tube was charged with bis(2,6-diisopropylphenoxy)phosphine borane (**2d**) (40 mg, 50 µmol) and KHMDS (22 mg, 60 µmol) and cooled to -78°C. Toluene-d_8 (0.6 mL) was slowly added maintaining the temperature. After homogenization, the mixture was investigated by NMR-spectroscopy. – ^{1}H NMR (Toluene-d_8): δ= 7.12-6.86 (m, aryl-H, 6 H), 3.73 (sept, $^3J_{HH}$ = 6.7 Hz, CH, 4 H), 1.26 (d, $^3J_{HH}$ = 7.0 Hz, CH_3, 12 H), 1.23 (d, $^3J_{HH}$ = 6.8 Hz, CH_3, 12 H). – ^{31}P{^{1}H} NMR (Toluene-d_8): δ= 325.8 (br). – ^{11}B{^{1}H} NMR (Toluene-d_8):

δ= -29.6 (d, $^{1}J_{PB}$ = 27 Hz). - $^{13}C\{^{1}H\}$ NMR (Toluene-d_8): δ = 159.9 (d, $^{2}J_{PC}$ = 6 Hz, *i*-C), 141.7 (d, $^{3}J_{PC}$ = 2 Hz, *o*-C), 123.9 (s, *m*-C) 123.5 (s, *p*-C), 27.6 (d, $^{4}J_{PC}$ = 5 Hz, CH), 24.3 (s, CH_3), 24.2 (s, CH_3).

In situ preparation of lithium diethoxyphosphide borane (Li[3b])

LiHMDS (25 mg, 0.15 mmol) was dissolved in THF-d_8 (0.6 mL) in an NMR tube. The solution was cooled to -78°C and **2b** (20 mg, 0.15 mmol) was added carefully. The sample was transferred into a precooled NMR spectrometer and characterised my multinuclear NMR spectra recorded at 203 K. – ^{1}H NMR (THF-d_8, 203 K): δ = 3.84-3.66 (m, 4 H, OCH_2), 1.12 (t, $^{3}J_{HH}$ = 7.0 Hz, 6 H, CH_3), 1.00-0.17 (broad, 3 H, BH_3). – ^{31}P NMR (THF-d_8, 203 K): δ = 294.5 (broad). – $^{11}B\{^{1}H\}$ NMR (THF-d_8, 203 K): δ = -33.5 ($^{1}J_{PB}$ = 26 Hz). – $^{13}C\{^{1}H\}$ NMR (THF-d_8, 203 K): δ = 64.4 (s, OCH_2), 17.3 (d, $^{3}J_{PC}$ = 3 Hz, CH_3). – ^{7}Li NMR (THF-d_8, 203 K): δ = -0.53 (s).

Lithium-1,1,3,3-tetraisopropoxy-triphosphide 1,2,3-tris-borane (Li[5c])

A solution of LiHMDS (153 mg, 0.91 mmol) in THF (4 mL) was cooled to -78 °C. Phosphine borane **2c** (150 mg, 0.91 mmol) was added dropwise and the solution was allowed to warm to room temperature. The solvent was removed and the crude product dissolved in hexane (1 mL). Storing the solution at -25°C afforded colourless crystals of Li[**5c**] (92 mg, 0.18 mmol, 19%). – ^{1}H NMR (C_6D_6): δ = 5.09-4.85 (m, 4 H, OCH), 3.54 (m, 6 H, THF), 2.36-1.15 (b, 43 H, BH_3, THF, CH_3). – ^{31}P NMR (C_6D_6): δ = 163.2 (broad d, $(iPrO)_2PBH_3$), -92.5 (t, $^{1}J_{PP}$ = 283 Hz, P*P*P). – ^{11}B NMR (C_6D_6): δ = -37.0 (broad). – $^{13}C\{^{1}H\}$ NMR (C_6D_6): δ = 72.9 (broad, OCH), 68.0 (s, THF), 25.1 (s, THF), 24.1 (b, CH_3), 23.8 (b, CH_3). – IR (cm^{-1}): ṽ = 2393, 2351, 2310(sh), 2275 (vBH).

Lithium-1,1,3,3-tetraethoxy-triphosphide 1,3-bis-borane (Li[6b])

A solution of LiHMDS (50 mg, 0.30 mmol) in THF (4 mL) was cooled to -78°C. Phosphine borane **2b** (40 mg, 0.30 mmol) was added dropwise and the mixture was warmed to room temperature. Volatiles were removed in vacuum. The oily residue was treated with hexane (1mL), and Et_2O was added until the product had completely dissolved. Storage at -25 °C produced single crystals suitable for an XRD study. For spectroscopic characterisation, the crude oily product obtained in another experiment was dissolved in THF-d_8 (0.5 mL). The main products were identified as Li[**6b**] and LiOEt/Li[$EtOBH_3$].

LiOEt/Li[$EtOBH_3$]: ^{1}H NMR (THF-d_8): δ = 3.54 (broad, 4 H, OCH_2), 1.69 (broad, 6 H, CH_3). – ^{11}B NMR (THF-d_8): δ = -10.7 (q, $^{1}J_{BH}$ = 87 Hz).

Li[**6b**]: ^{1}H NMR (THF-d_8): δ = 4.08-3.87 (m, 8 H, OCH_2), 1.17 (t, $^{3}J_{HH}$ = 7.0 Hz, 12 H, CH_3), 1.00-0.17 (broad, 6 H, BH_3),. – $^{31}P\{^{1}H\}$ NMR (THF-d_8): δ = 183.7 (dq, $^{1}J_{PP}$ = 466 Hz, $^{1}J_{PB}$ = 85 Hz, $(EtO)_2\mathbf{P}BH_3$), -144.5 (t, $^{1}J_{PP}$ = 466 Hz, P**P**P). – $^{11}B\{^{1}H\}$ NMR (THF-d_8): δ = -34.6 (d, $^{1}J_{PB}$ = 85 Hz). – ^{7}Li NMR (THF-d_8): δ = -0.13 (s).

Lithium-1,1,3,3-tetraisopropoxy-triphosphide 1,3-bis-borane (Li[6c])

A solution of Li[**5c**] (50 mg, 0.10 mmol) in NEt_3 (2 mL) was stirred for 18 h at room temperature. Volatiles were removed under reduced pressure and the residue dissolved in C_6D_6 (0.6 mL). NMR studies revealed the presence of a mixture of Li[**6c**] and Et_3NBH_3.. Data for Li[**6c**]: ^{1}H NMR (C_6D_6): δ = 5.05-4.83 (m, 4 H, OCH), 1.32 (d, $^{3}J_{HH}$ = 6 Hz, 6 H CH_3), 1.31 (d, $^{3}J_{HH}$ = 6 Hz, 6 H, CH_3). – $^{31}P\{^{1}H\}$ NMR (C_6D_6): δ = 180.2 (broad d, $(iPrO)_2PBH_3$), -127.4 (t, $^{1}J_{PP}$ = 449 Hz, P*P*P). – $^{11}B\{^{1}H\}$ NMR (C_6D_6): δ = -33.2 (d, $^{1}J_{PB}$ = 70 Hz). – $^{13}C\{^{1}H\}$ NMR (C_6D_6): δ = 68.7 (m, OCH), 22.1 (d, $^{3}J_{PC}$ = 4 Hz, CH_3), 22.0 (d, $^{3}J_{PC}$ = 4 Hz, CH_3).

Reaction of lithium diethoxyphosphide borane Li[3b] with BuLi

A solution of Li[**3b**] was prepared by dropwise addition of **2b** (75 mg, 0.55 mmol) to a solution of LiHMDS (92 mg, 0.55 mmol) in THF (4 mL) at -78°C. After the solution had been stirred for additional 15 minutes, BuLi (1 mL of a 2.5 M solution in hexanes, 2.48 mmol) was added dropwise. ^{31}P reaction monitoring disclosed the formation of a new product in an instantaneous reaction at -50 °C. The mixture was then allowed to warm to room temperature. Characterisation by NMR spectroscopy allowed to assign the reaction product as Li[$Bu_2P(BH_3)$] (Li[**7**]; $^{31}P\{^{1}H\}$ (THF): δ = -72.7 (q, $^{1}J_{PB}$ =37 Hz),$^{11}B\{^{1}H\}$ (THF): δ = -31.4 (d, $^{1}J_{PB}$ =37 Hz)). The mixture was re-cooled to -78°C, treated with MeOH (1 mL), and once more allowed to warm to room temperature. Volatiles were removed in vacuum. The residue was dissolved in petroleum ether/ethyl acetate (1:1), the solution filtered over silica, and the filtrate evaporated to dryness. Dibutylphosphine borane **8** was obtained as colourless oil (67 mg, 0.42 mmol, 76 %). The NMR spectra were in accordance with literature data.[5]

Diethoxytriphenylstannylphosphine borane (9b)

Potassium diethoxyphospanide borane K[**3b**] (181 mg, 1.04 mmol) and Ph_3SnCl (400 mg, 1.04 mmol) were suspended in toluene (20 mL). The mixture was stirred for 18 h and filtered. The filtrate was evaporated to dryness and the residue suspended in hexane (5 mL). The solvent was filtered off and the residue dried in vacuum. Colourless solid (238 mg, 491 µmol, 47%; m.p.(dec.) 72 °C). Single crystals suitable for X-Ray crystallography were grown from a concentrated hexane solution. – ^{1}H NMR (C_6D_6): δ = 7.90-7.69 (m, 6 H, Ph), 7.27-7.00 (m, 9 H, Ph), 4.10-3.70 (m, 4 H, OCH_2), 2.62-1.05 (b, 3 H, BH_3), 0.89 (t, $^{3}J_{HH}$ = 7.0 Hz, 6 H, CH_3). – $^{31}P\{^{1}H\}$ NMR (C_6D_6): δ = 182.0 (b). $^{11}B\{^{1}H\}$ NMR (C_6D_6): δ = -35.7 (d, $^{1}J_{PB}$ = 40 Hz). – δ = 137.4 (d, $^{3}J_{PC}$ = 2 Hz, *o*-Ph), 136.3 (d, $^{2}J_{PC}$ = 13 Hz, *i*-Ph) 129.6 (s, Ph), 128.9 (s, Ph), 64.8 (d, $^{2}J_{PC}$ = 9 Hz, OCH_2), 16.2 (d, $^{3}J_{PC}$ = 3 Hz, CH_3). – $^{119}Sn\{^{1}H\}$ NMR (C_6D_6): δ = 183 (d, $^{1}J_{PSn}$ = 320 Hz). – IR (cm^{-1}): ṽ = 2397, 2362, 2323 (vBH). – $C_{24}H_{32}BO_2PSn$ (512.01 g mol^{-1}): calcd. C 54.49 H 5.82, found C 54.52 H 5.83.

Diisopropoxytriphenylstannylphosphine borane (9c)

The synthesis was performed as described for **9b** with K[**3c**] (210 mg, 1.04 mmol) and Ph_3SnCl (400 mg, 1.04 mmol). Colourless solid (200 mg, 391 µmol, 38%; m.p.(dec.) 89 °C). Single crystals suitable for X-Ray crystallography were grown from a concentrated hexane solution. – ^{1}H NMR (C_6D_6): δ = 7.92-7.70 (m, 6 H, Ph), 7.30-7.04 (m, 9 H, Ph), 4.72 (dsept, $^{3}J_{HH}$ = 6 Hz, $^{3}J_{PH}$ = 10 Hz, 2 H,

OCH), 0.9 (broad, 3 H, BH_3), 1.05 (d, $^3J_{HH}$ = 6.2 Hz, 6 H, CH_3), 1.02 (d, $^3J_{HH}$ = 6.2 Hz, 6 H, CH_3). – $^{31}P\{^1H\}$ NMR (C_6D_6): δ = 178.4 (broad). – $^{11}B\{^1H\}$ NMR (C_6D_6): δ = -32.4 (d, $^1J_{PB}$ = 37 Hz). – $^{13}C\{^1H\}$ NMR (C_6D_6): δ = 138.0 (d, $^3J_{PC}$ = 2 Hz, *o*-Ph), 137.1 (d, $^2J_{PC}$ = 13 Hz, *i*-Ph), 130.1 (s, *p*-Ph), 129.5 (d, $^4J_{PC}$ = 1 Hz, *m*-Ph), 74.0 (d, $^2J_{PC}$ = 9 Hz,OCH), 24.3 (d, $^3J_{PC}$ = 4 Hz, CH_3), 24.2 (d, $^3J_{PC}$ = 4 Hz, CH_3). – $^{119}Sn\{^1H\}$ NMR (C_6D_6): δ = -138.5 (d, $^1J_{PSn}$ = 364 Hz). – IR (cm^{-1}): ṽ = 2388, 2339 (vBH). – (+)ESI-MS: m/z 537.1154 (MNa^+, calcd. 537.1155), 553.0888 (MK^+, calcd. 583.0893). – $C_{24}H_{32}BO_2PSn$ (512.01 g·mol^{-1}): calcd. C 56.19 H 6.29, found C 55.38 H 6.08.

Bis(2,6-diisopropylphenoxy)triphenylstannylphosphine borane (9d)

Phosphine borane **2d** (250 mg, 0.62 mmol) and KHMDS (125 mg, 0.62 mmol) were dissolved in toluene (12 mL) at -78°C. The mixture was stirred for 1h at -78 °C and then allowed to warmed to room temperature until it became homogeneous. After re-cooling to -78 °C, a solution of Ph_3SnCl (241 mg, 0.62 mmol) in toluene (2 mL) was slowly added. The mixture was stirred for 1 h at -78°C and then for 1 h at room temperature. Volatiles were removed in vacuum and the residue treated with hexane (5 mL). The resulting suspension was filtered. Evaporation of the filtrate to dryness afforded a colourless solid (285 mg, 380 µmol, 60%). Single crystal suitable for X-Ray crystallography were grown from a saturated hexane solution.

^1H NMR (C_6D_6): δ = 7.83-7.52 (m, 6 H, SnPh), 7.13-7.07 (m, 9 H, Sn-Ph), 7.01-6.93 (m, 6 H, OC_6H_3), 3.58 (sept, $^3J_{HH}$ = 6.8 Hz, 4 H, CH), 1.12 (d, $^3J_{HH}$ = 6.8 Hz, 12 H, CH_3), 0.91 (d, $^3J_{HH}$ = 6.7 Hz, 12H, CH_3), 2.5-0.5 (broad, 3 H, BH_3). – $^{31}P\{^1H\}$ NMR (C_6D_6): δ = 197.9 (broad). – $^{11}B\{^1H\}$ NMR (C_6D_6): δ = -31.4 (broad). – $^{13}C\{^1H\}$ NMR (C_6D_6): δ = 148.8 (d, $^2J_{PC}$ = 14 Hz, *i*-OAryl), 141.0 (d, $^5J_{PC}$ = 3 Hz, *p*-SnPh), 137.5 (d, $^2J_{SnC}$ = 40 Hz, $^3J_{PC}$ = 2 Hz, *o*-SnPh), 136.5 (d, $^3J_{PC}$ = 13 Hz, *o*-OAryl), 129.6 (s, $^4J_{SnC}$ = 12 Hz, *m*-SnPh), 128.9 (s, $^1J_{SnC}$ = 55 Hz, *i*-SnPh), 126.0 (d, $^4J_{PC}$ = 2 Hz, *m*-OAryl), 124.4 (d, $^5J_{PC}$ = 2 Hz, *p*-OAryl), 27.9 (s, CH), 23.9 (s, CH_3), 22.9 (s, CH_3). – $^{119}Sn\{^1H\}$ NMR (C_6D_6): δ = -176.2 (d, $^1J_{PSn}$ =77 Hz). – IR (cm^{-1}): ṽ = 2444, 2383 (vBH). – $C_{42}H_{52}BO_2PSn$ (749.37 g·mol^{-1}): calcd. C 67.32 H 6.99, found C 67.25 H 7.03.

Diethoxytriphenylstannylphosphine (10b)

In an NMR tube, phosphine borane **9b** (25 mg, 52 µmol) and DABCO (27 mg, 258 µmol) were dissolved in C_6D_6 (0.6 mL). The solution was heated to 50 °C in an oil bath and NMR spectra were measured after 0, 30, 60 and 90 minutes. Reaction products were identified in situ by their NMR data. – $^{31}P\{^1H\}$ NMR: δ = 239.0 (s, $^1J_{PSn}$ = 860 Hz, **10b**), 182.9 (s, $EtO)_2PP(OEt)_2$). – $^{119}Sn\{^1H\}$ NMR: δ = -198.9, $^1J_{PSn}$ = 860 Hz, **10b**), -141.3 (s, Sn_2Ph_6). – $^{11}B\{^1H\}$ NMR: δ = -10.2 (s, DABCO·BH_3).

Diisopropoxytriphenylstannylphosphine (10c)

The experiment was carried out as described for **10b** using **9c** (25 mg, 49 µmol) and DABCO (27 mg, 244 µmol). – $^{31}P\{^1H\}$ NMR: δ = 231.3 (s, $^1J_{PSn}$ = 830 Hz, **10c**), 176.7 (s, $iPrO)_2PP(OiPr)_2$). – $^{119}Sn\{^1H\}$ NMR: δ = -197.5, $^1J_{PSn}$ = 830 Hz, **10c**), -141.3 (s, Sn_2Ph_6). – $^{11}B\{^1H\}$ NMR: δ = -10.2 (s, DABCO·BH_3).

Bis(2,6-diisopropylphenoxy)triphenylstannylphosphine (10d)

A solution of **9d** (50 mg, 67 µmol) in toluene (3 mL) and NEt_3 (1.5 mL) was stirred for 16 h. Volatiles were removed under reduced pressure and the residue treated with pentane (3 mL). Insoluble components were removed by filtration and the volume of the filtrate was reduced to 0.5 mL. The product separated as colourless crystals (40 mg, 54 µmol, 82 %). – ^1H NMR (C_6D_6): δ = 7.91-7.68 (m, 6 H, SnPh), 7.25-6.99 (m, 15 H, SnPh and OAryl), 3.45 (sept, $^3J_{HH}$ = 6.8 Hz, 4 H, CH), 1.01 (d, $^3J_{HH}$ = 6.8 Hz, 12 H, CH_3), 0.97 (d, $^3J_{HH}$ = 6.8 Hz, 12 H, CH_3). – $^{31}P\{^1H\}$ NMR (C_6D_6): δ = 273.0 (s, $^1J_{PSn}$ = 770 Hz). – $^{119}Sn\{^1H\}$ NMR (C_6D_6): δ = -185.2 (d, $^1J_{PSn}$ = 770 Hz). – $^{13}C\{^1H\}$ NMR (C_6D_6): δ = 152.3 (d, $^2J_{PC}$ = 1 Hz, *i*-OAryl), 140.7 (d, $^3J_{PC}$ = 2 Hz, *m*-OAryl), 139.8 (d, $^3J_{PC}$ = 3 Hz, *o*-SnPh), 138.0 (s, $^1J_{SnC}$ = 38 Hz *i*-SnPh), 129.3 (s, *p*-SnPh), 129.1 (s, *m*-SnPh), 125.2 (d, $^4J_{PC}$ = 1 Hz, *p*-OAryl), 124.5 (d, $^4J_{PC}$ = 1 Hz, *m*-OAryl), 28.2 (d, $^4J_{PC}$ = 6 Hz, CH), 24.0 (d, $^5J_{PC}$ = 1 Hz, CH_3), 23.9 (broad, CH_3). – (+)ESI-MS: m/z 775.2123 (MK^+, calcd. 775.2131). – $C_{42}H_{49}O_2PSn$ (735.54 g·mol^{-1}): calcd. C 68.58 H 6.72, found C 68.60 H 6.72.

Crystallographic studies

X-ray diffraction data were collected on a Bruker Kappa Apex II Duo diffractometer equipped with an APEX II CCD-detector and a KRYO-FLEX cooling device with Mo-K_α radiation (λ = 0.71073 Å) at 130(2) K (K[**3d**]·K[$N(SiMe_3)_2$]$(THF)_2$), 135(2) K (**2d**, Li[**5c**], **9b-d**) or 140(2) K (Li[**6b**], **10d**), respectively. The structures were solved with direct methods (SHELXS-2014 [7]) and refined with a full-matrix least squares scheme on F^2 (SHELXL-2014 [7]). Semi-empirical or numerical absorption corrections (see Table S1) were applied. Non-hydrogen atoms were refined anisotropically and hydrogen atoms except those bound to phosphorus and boron using a riding model. One $SiMe_3$ moiety and the THF moieties in K[**3b**]-$KNTms_2$, the ethoxy groups in **9b**, and two iPr-groups in **9d** as well as all iPr-groups in **10d** are disordered. Further details on the refinement of the disorder is given in the cif-files and the incorporated res-files. CCDC-2046650 to CCDC-2046657 contain the crystallographic data for this paper, which can be obtained free of charge from the Cambridge Crystallographic Data Centre via www.ccdc.cam.ac.uk/data_request/cif.

Table S1 Crystallographic data for **2d**, [K(THF)][**3d**]·[K(THF)][N(SiMe$_3$)$_2$], Li[**5c**],Li[**6b**], **9b-d**, **10d.**

	**2d**	K[**3d**]·K[N(SiMe$_3$)$_2$](THF)$_2$	Li[**5c**]	Li[**6b**]
CCDC	2046654	2046650	2046652	2046657
Empirical formula	$C_{24}H_{38}BO_2P$	$C_{18}H_{47}BK_2NO_4PSi_2$	$C_{20}H_{53}B_3LiO_6P_3$	$C_8H_{26}B_2LiO_4P_3$
Formula weight/g mol^{-1}	400.32	517.72	521.90	307.76
T/K	135(2)	130(2)	135(2)	140(2)
Wavelength/Å	0.71073	0.71073	0.71073	0.71073
Crystal system	triclinic	monoclinic	monoclinic	triclinic
Space group	$P\bar{1}$	$P2_1/n$	$P2_1/c$	$P\bar{1}$
a/Å	9.4978(4)	12.4127(4)	16.8630(5)	8.7094(5)
b/Å	10.8815(5)	10.6275(3)	9.6999(3)	10.1661(6)
c/Å	12.4747(6)	23.2684(8)	19.8825(6)	10.7159(6)
α/°	74.228(3)	90	90	108.372(2)
β/°	88.499(2)	101.947(2)	103.554(2)	99.768(3)
γ/°	77.850(2)	90	90	103.710(3)
V/Å^{-3}	1212.27(10)	3002.99(17)	3161.59(17)	843.49(9)
Z	2	4	4	2
ρ_{calcd}/Mg m^{-3}	1.097	1.145	1.096	1.212
Absorption coeff./mm^{-1}	0.129	0.469	0.216	0.352
F(000)	436	1120	1136	328
Crystal size/mm^3	0.571 x 0.443 x 0.428	0.602 x 0.552 x 0.294	0.340 x 0.270 x 0.220	0.366 x 0.226 x 0.206
Θ-range for data collection/°	1.697 to 25.242	1.729 to 28.375	2.107 to 28.338	2.077 to 30.681
Index ranges	-14≤h≤14<br>-16≤k≤16<br>-19≤l≤19	-16≤h≤16,<br>-14≤k≤14,<br>-31≤l≤31	-22≤h≤22,<br>-12≤k≤12,<br>-26≤l≤26	-12 ≤ h ≤ 12<br>-14 ≤ k ≤ 14<br>-15 ≤ l ≤ 14
Refl. collected	35409	51893	31949	26856
Independent refl.	9113	7488	7851	5198
Completeness to θ = 25.242°	97.7%	99.6%	99.6%	99.2%
Abs. correction	semi-empirical	semi-empirical	semi-empirical	semi-empirical
Max. and min. transmission	0.7465 and 0.7163	0.7457 and 0.6633	0.7457 and 0.7002	0.7461 and 0.7107
Refinement method	Full-matrix least-squares on F^2	Full-matrix least-squares on F^2	Full-matrix least-squares on F^2	Full-matrix least-squares on F^2
Data / restraints / parameters	9113 / 3 / 265	7488 / 841 / 379	7851 / 9 / 325	5198 / 6 / 187
G.o.f. on F^2	1.035	1.066	1.021	1.044
Final R indices [I > 2σ(*I*)]	R1 = 0.0453<br>wR2 = 0.1175	R1 = 0.0367<br>wR2 = 0.0849	R1 = 0.0328<br>wR2 = 0.0769	R1 = 0.0305<br>wR2 = 0.0711
R indices (all data)	R1 = 0.0609<br>wR2 = 0.1296	R1 = 0.0545<br>wR2 = 0.0946	R1 = 0.0507<br>wR2 = 0.0834	R1 = 0.0461<br>wR2 = 0.0774
Largest diff. peak and hole/e Å^{-3}	0.697 and -0.422	0.573 and -0.278	0.314 and -0.243	0.390 and -0.280

Table S1 (cont.)

	9b	9c	9d	10d
CCDC	2046651	2046655	2046653	2046656
Empirical formula	$C_{22}H_{28}BO_2PSn$	$C_{24}H_{32}BO_2PSn$	$C_{42}H_{52}BO_2PSn$	$C_{42}H_{49}O_2PSn$
Formula weight/g mol^{-1}	484.91	512.96	749.30	735.47
T/K	135(2)	135(2)	135(2)	140(2)
Wavelength/nm	0.71073	0.71073	0.71073	0.71073
Crystal system	monoclinic	triclinic	triclinic	Monoclinic
Space group	$P2_1/n$	$P\overline{1}$	$P\overline{1}$	$P2_1/n$
a/Å	10.4232(5)	9.6946(4)	11.2181(6)	11.4262(7)
b/Å	16.3193(8)	10.8325(5)	11.2767(6)	16.5238(9)
c/Å	13.5035(6)	12.8287(6)	17.5903(9)	19.8992(11)
α/°	90	73.894(2)	74.358(3)	90
β/°	101.992(2)	79.830(2)	73.428(2)	92.342(2)
γ/°	90	72.892(3)	70.064(2)	90
V/Å^{-3}	2246.81(18)	1230.44(10)	1967.96(18)	3753.9(4)
Z	4	2	2	4
ρ_{calcd}/Mg m^{-3}	1.434	1.385	1.265	1.301
Absorption coeff./mm^{-1}	1.222	1.120	0.722	0.756
F(000)	984	524	780	1528
Crystal size/mm^3	0.812 x 0.276 x 0.174	0.476 x 0.257 x 0.130	0.938 x 0.583 x 0.468	0.200 x 0.175 x 0.166
Θ-range for data collection/°	1.984 to 25.026	1.661 to 30.583	1.957 to 33.274	1.602 to 25.027
Index ranges	-12 ≤ h ≤ 11<br>-15 ≤ k ≤ 19<br>-16 ≤ l ≤ 16	-13 ≤ h ≤ 13<br>-15 ≤ k ≤ 14<br>-18 ≤ l ≤ 18	-14 ≤ h ≤ 17<br>-16 ≤ k ≤ 17<br>-27 ≤ l ≤ 27	-13 ≤ h ≤ 13<br>-16 ≤ k ≤ 19<br>-20 ≤ l ≤ 23
Refl. collected	26536	34158	63478	30664
Independent refl.	3962	7552	14987	6591
Completeness to θ = 25.242°/66.548°	99.9%	99.8%	98.9%	99.4%
Absorption correction	numerical	semi-empirical	numerical	semi-empirical
Max. and min. transmission	0.8512 and 0.5041	0.6421 and 0.7461	0.8082 to 0.6540	0.9605 to 0.8431
Refinement method	Full-matrix least-squares on F^2	Full-matrix least-squares on F^2	Full-matrix least-squares on F^2	Full-matrix least-squares on F^2
Data / restraints / parameters	3962 / 117 / 291	7552 / 3 / 271	14987 / 153 / 470	6591 / 142 / 451
G.o.f. on F2	1.124	1.043	1.125	1.090
Final R indices [I > 2σ(*I*)]	R1 = 0.0178<br>wR2 = 0.0378	R1 = 0.0188<br>wR2 = 0.0417	R1 = 0.0267<br>wR2 = 0.0619	R1 = 0.0363<br>wR2 = 0.0810
R indices (all data)	R1 = 0.0224<br>wR2 = 0.0405	R1 = 0.0232<br>wR2 = 0.0436	R1 = 0.0339<br>wR2 = 0.0666	R1 = 0.0599<br>wR2 = 0.0937
Largest diff. peak and hole/e Å^{-3}	0.301 and -0.422	0.464 and -0.298	0.949 and -0.522	1.445 and -0.516

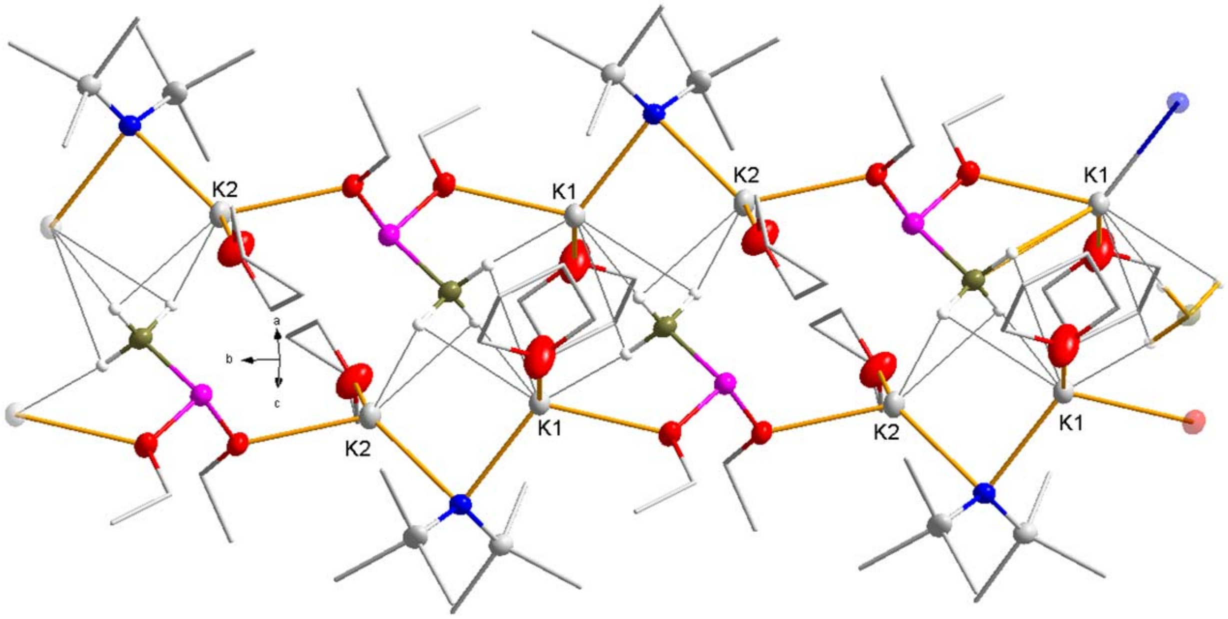

Figure S1: Representation of a section of the coordination polymeric structure in crystalline [K(THF)][**3b**]·[K(THF)][N(SiMe$_3$)$_2$]. Thermal ellipsoids are drawn at the 50% probability level. For clarity, carbon atoms are drawn using a wire model, hydrogen atoms except those in BH$_3$-units are omitted, and only one orientation of disordered fragments (one SiMe$_3$ group and one THF) is shown.

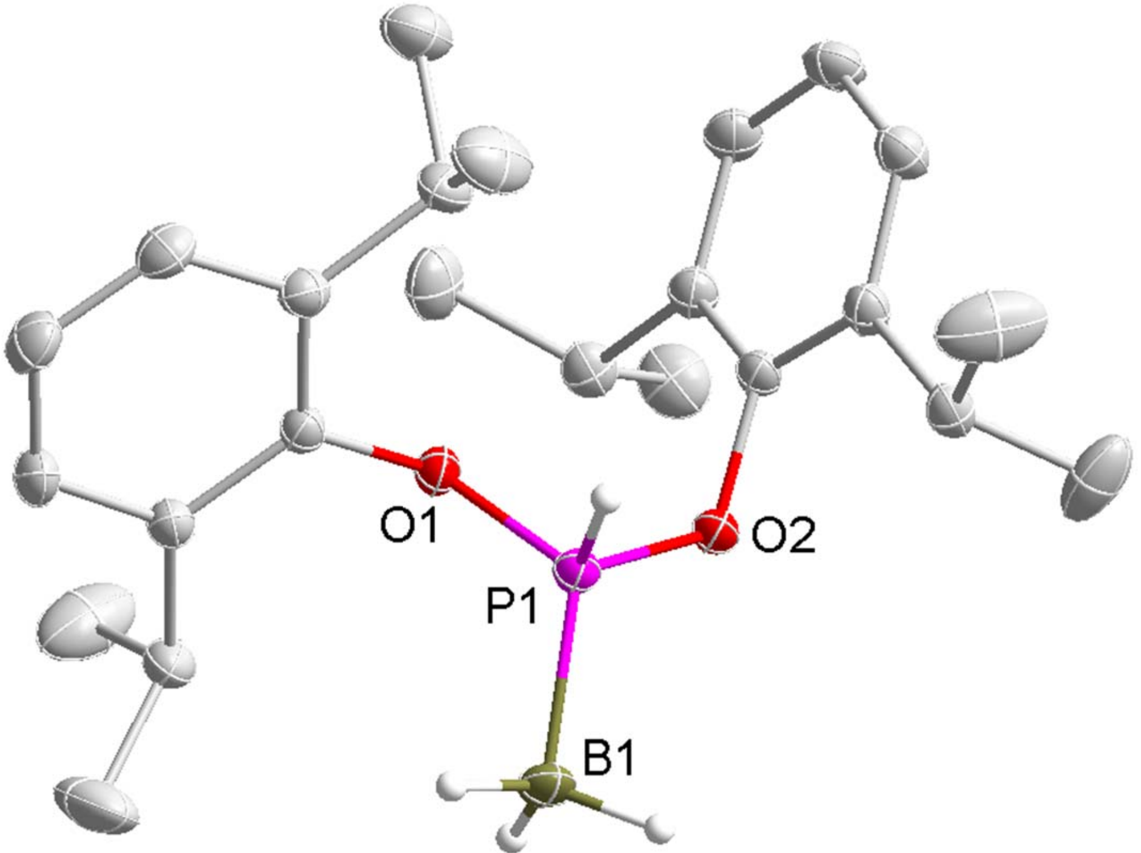

Figure S2: Representation of the molecular structure of **2d** in the crystal. Thermal ellipsoids are drawn at the 50% probability level. For clarity, hydrogen atoms except those in PH- and BH$_3$-units are omitted. Selected distances [Å]: P1–O2 1.5903(7), P1–O1 1.5987(7), P1–B1 1.8697(11), P1–H1 1.347(13).

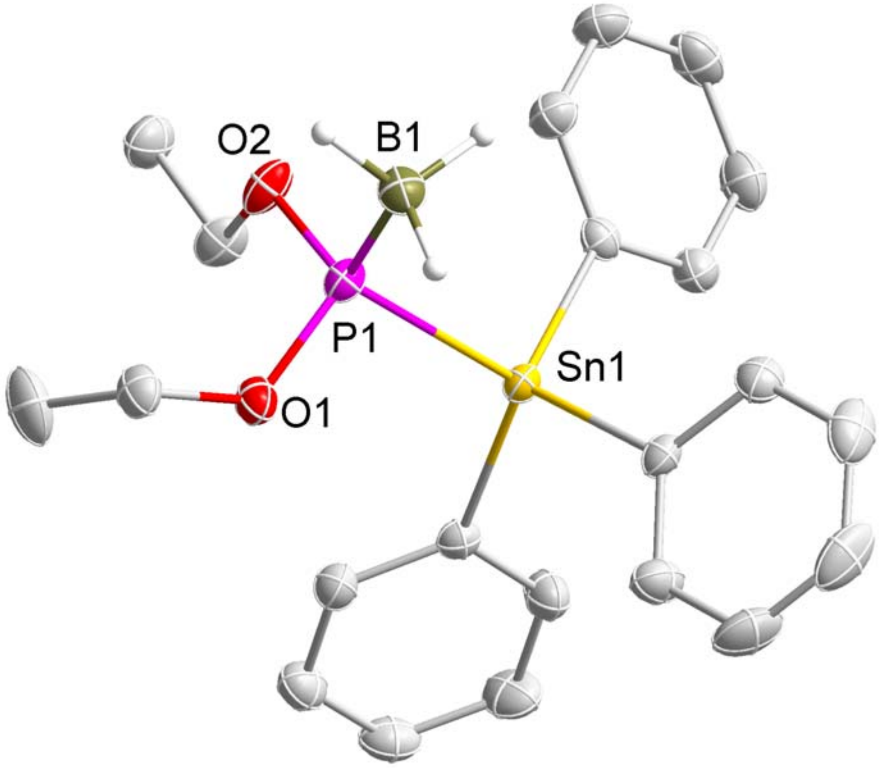

Figure S3: Representation of the molecular structure of **9b** in the crystal. Thermal ellipsoids are drawn at the 50% probability level. For clarity, hydrogen atoms except those in BH_3-units are omitted. Selected distances [Å] and angles [°]: P1–O2 1.5927(14), P1–O1 1.6007(15), P1–B1 1.890(2), P1–Sn1 2.5324(5), O1–P1–Sn1 99.95(5), O2–P1–Sn1 109.44(5), O1–P1–O2 106.64(8).

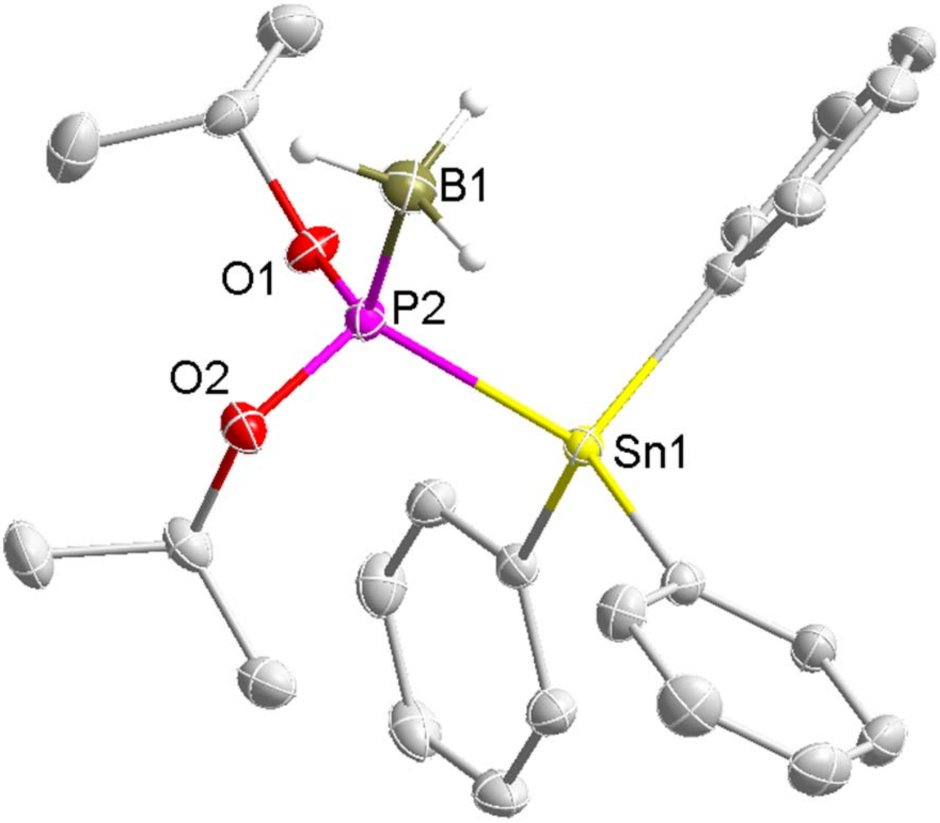

Figure S4: Representation of the molecular structure of **9c** in the crystal. Thermal ellipsoids are drawn at the 50% probability level. For clarity, hydrogen atoms except those in BH_3-units are omitted. Selected distances [Å] and angles [°]: P2–O2 1.5946(10), P2–O1 1.6015(9), P2–B1 1.8946(15), P2–Sn1 2.5193(3), O1–P2–Sn1 96.49(4), O2–P2–Sn1 112.88(4), O1–P2–O2 107.76(5).

NMR-Spectra

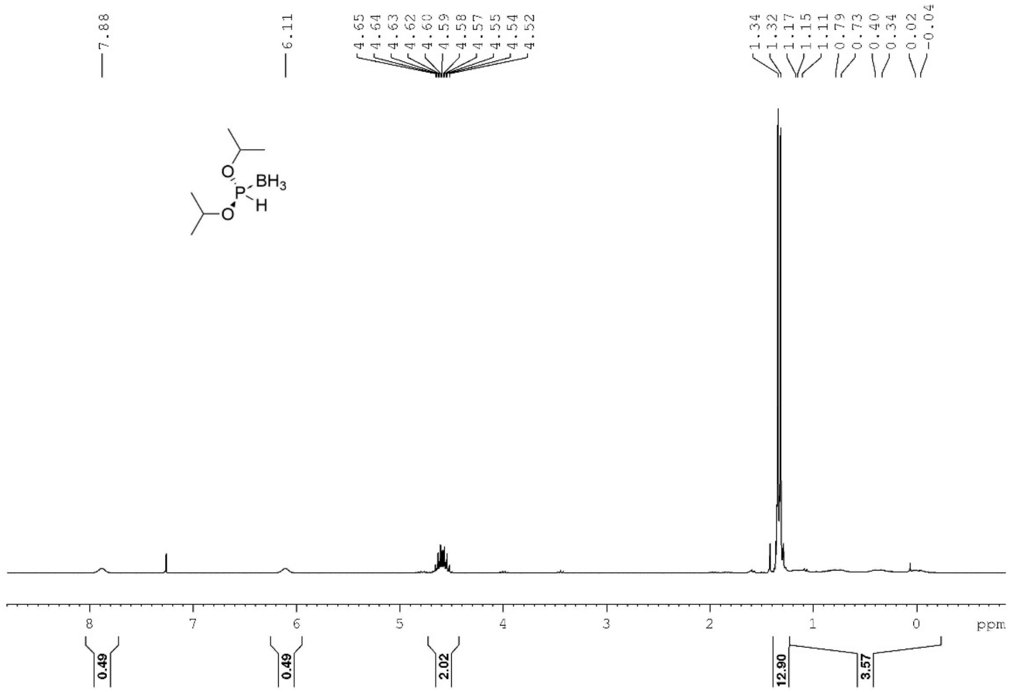

Figure S5: ^{1}H NMR spectrum of **2c** in $CDCl_3$.

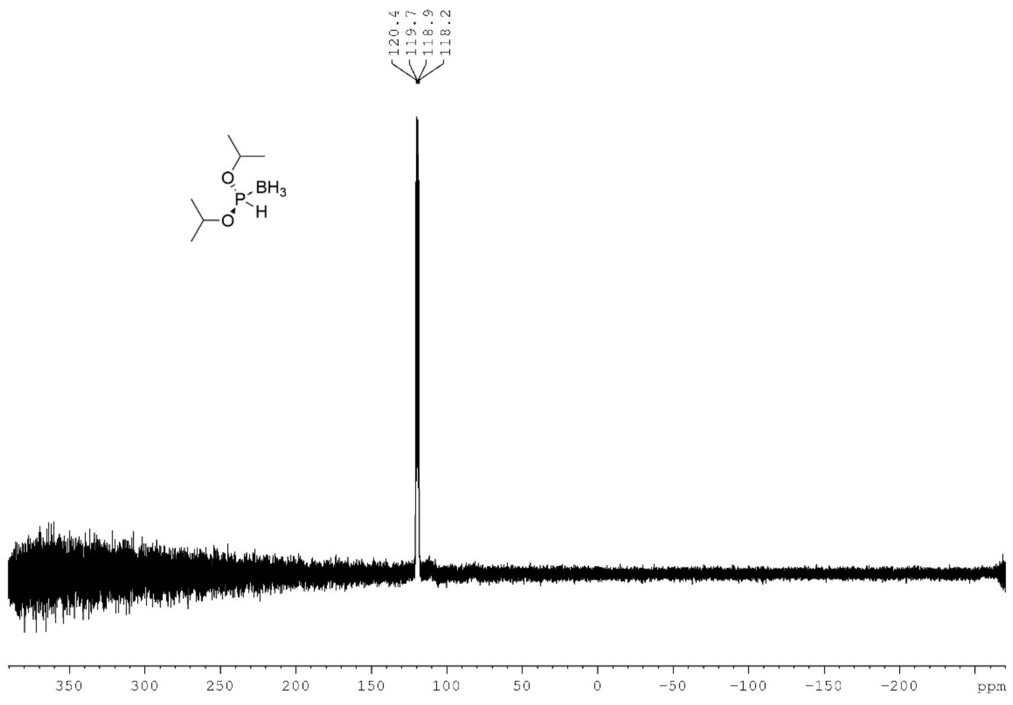

Figure S6: $^{31}P\{^{1}H\}$ NMR spectrum of **2c** in $CDCl_3$.

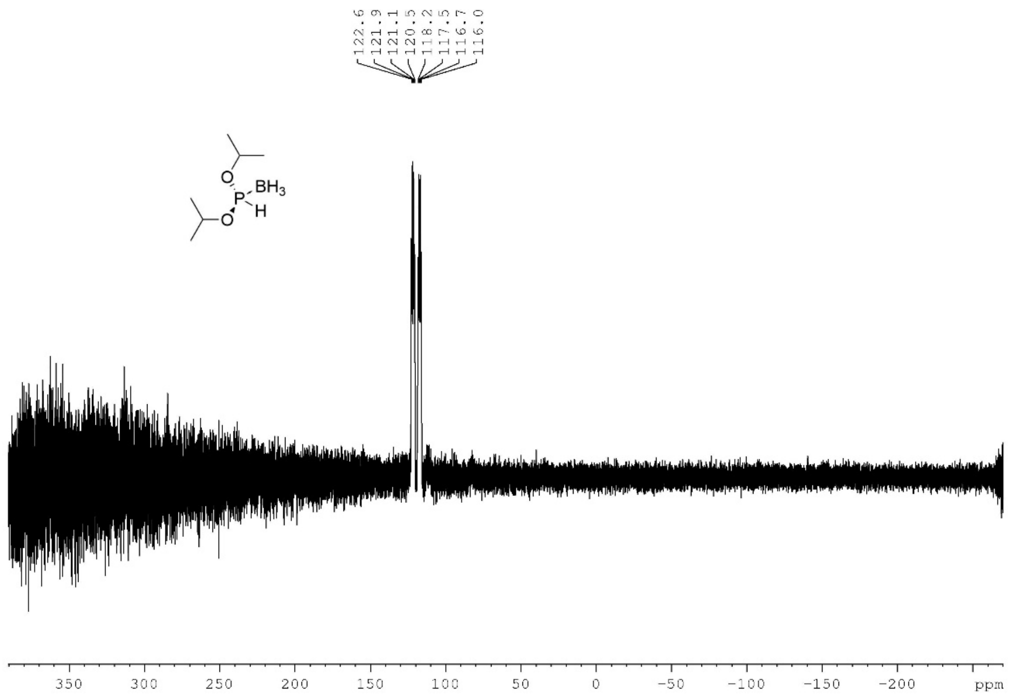

Figure S7: ^{31}P NMR spectrum of **2c** in $CDCl_3$.

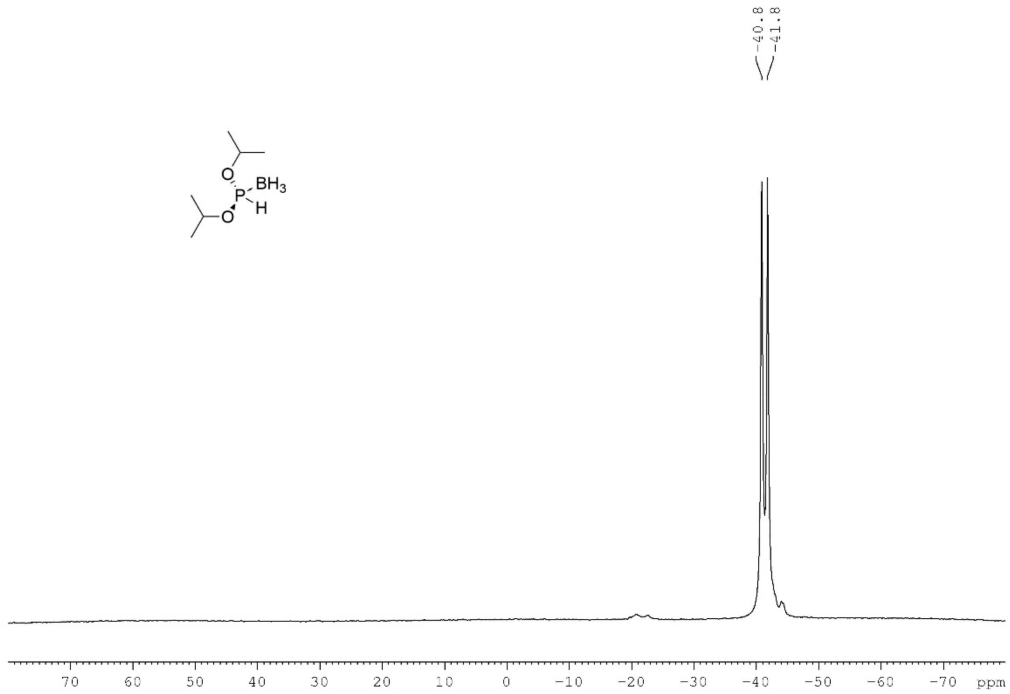

Figure S8: $^{11}B\{^{1}H\}$ NMR spectrum of **2c** in $CDCl_3$.

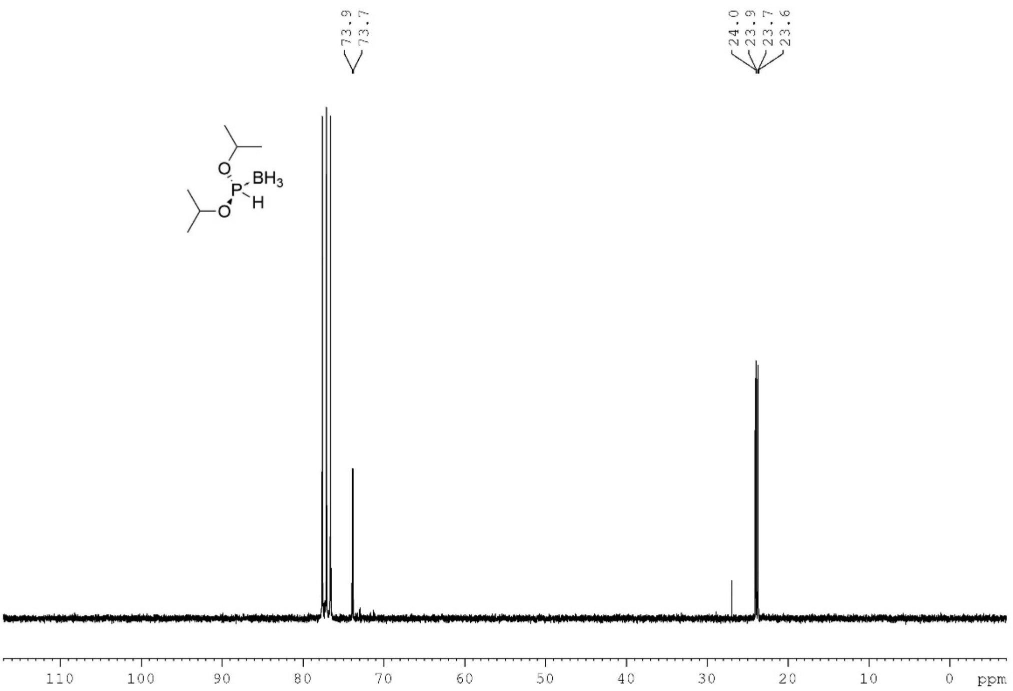

Figure S9: $^{13}C\{^{1}H\}$ NMR spectrum of **2c** in $CDCl_3$.

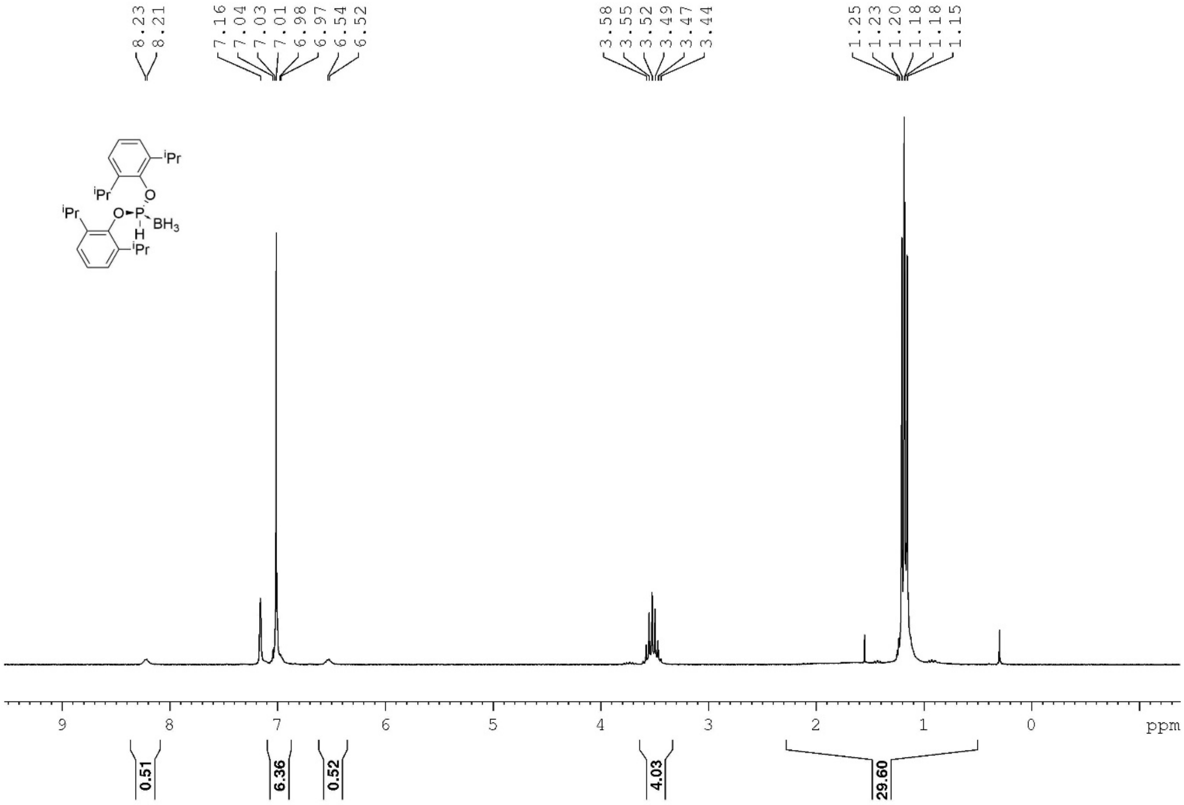

Figure S10: ^{1}H NMR spectrum of **2d** in C_6D_6.

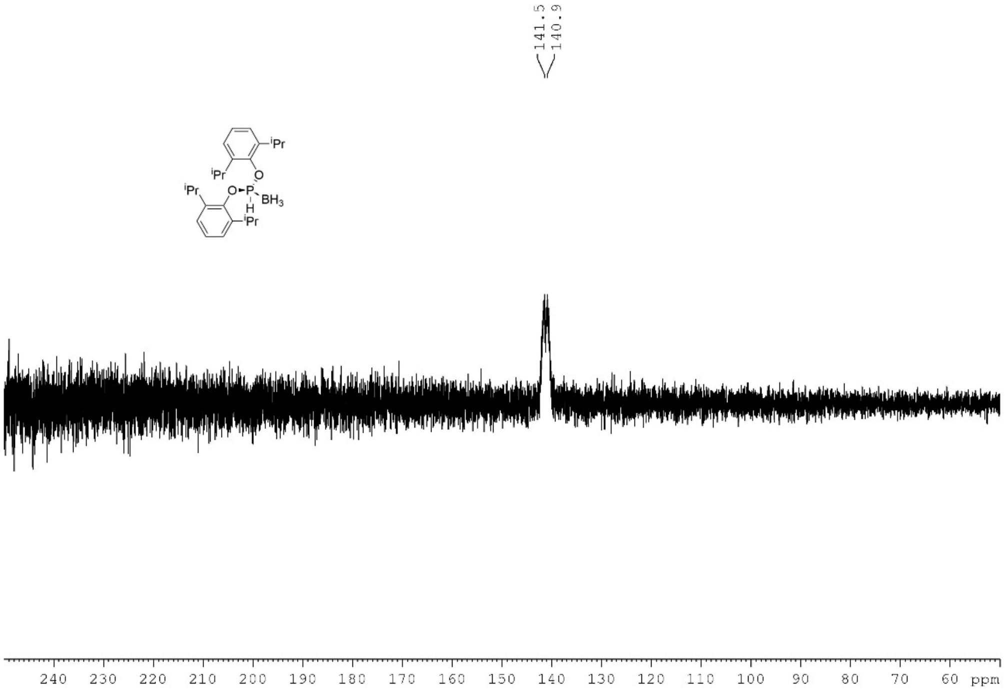

Figure S11: $^{31}P\{^1H\}$ NMR spectrum of **2d** in C_6D_6.

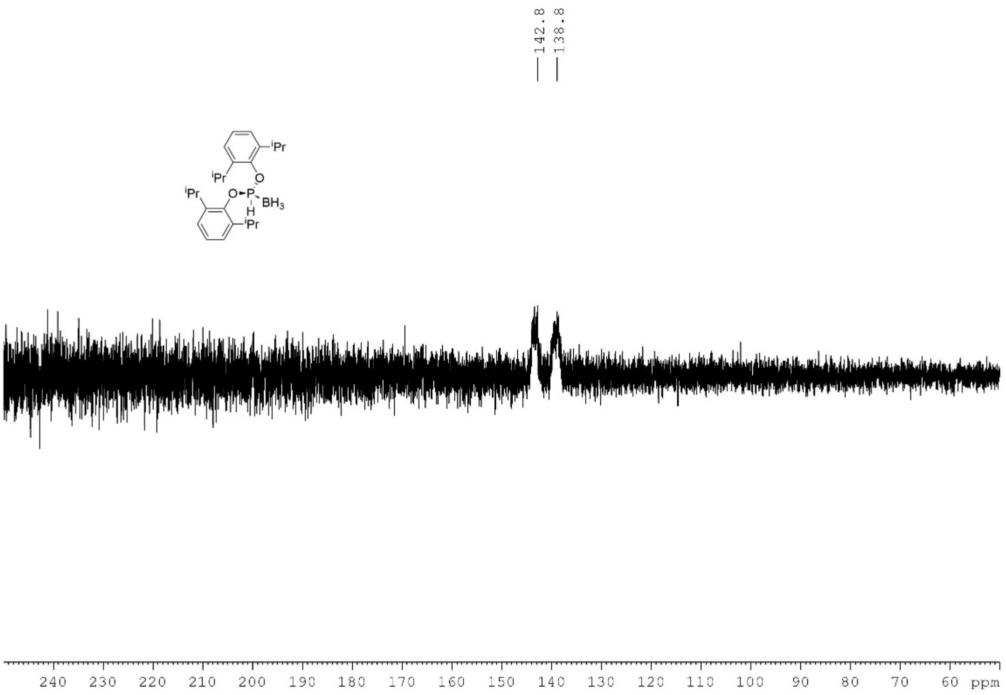

Figure S12: ^{31}P NMR spectrum of **2d** in C_6D_6.

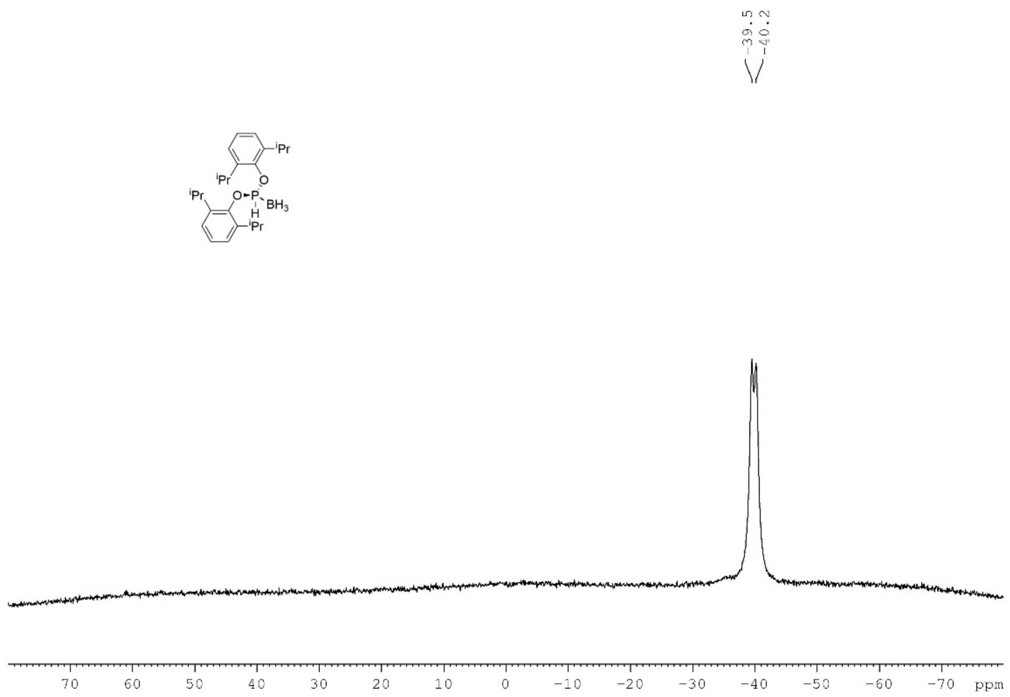

Figure S13: $^{11}B\{^{1}H\}$ NMR spectrum of **2d** in C_6D_6.

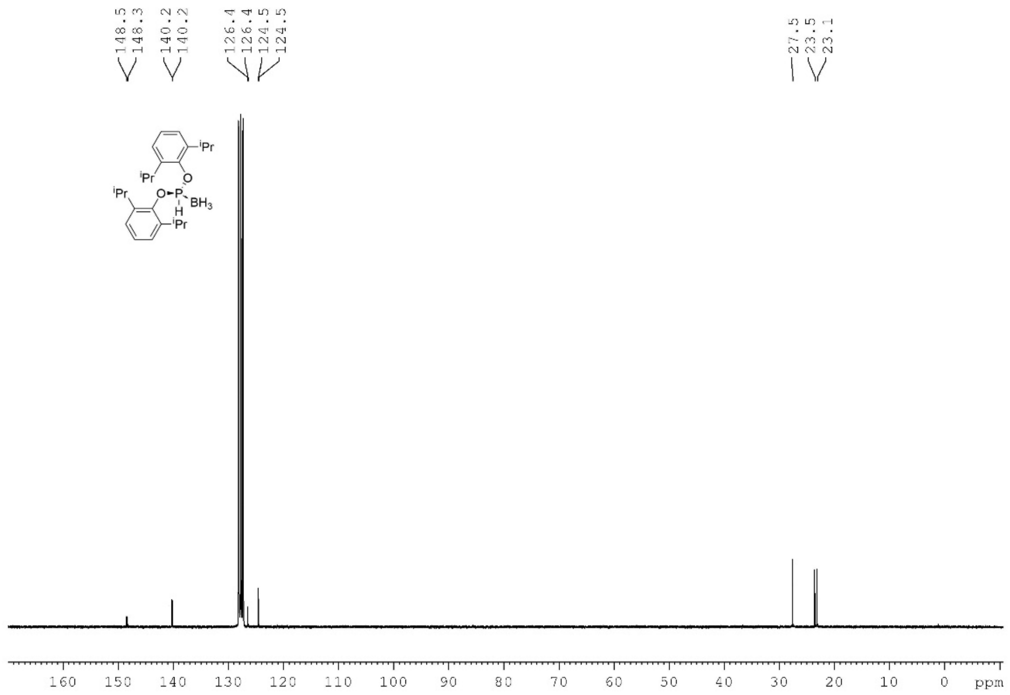

Figure S14: ^{13}C $\{^{1}H\}$ NMR spectrum of **2d** in C_6D_6.

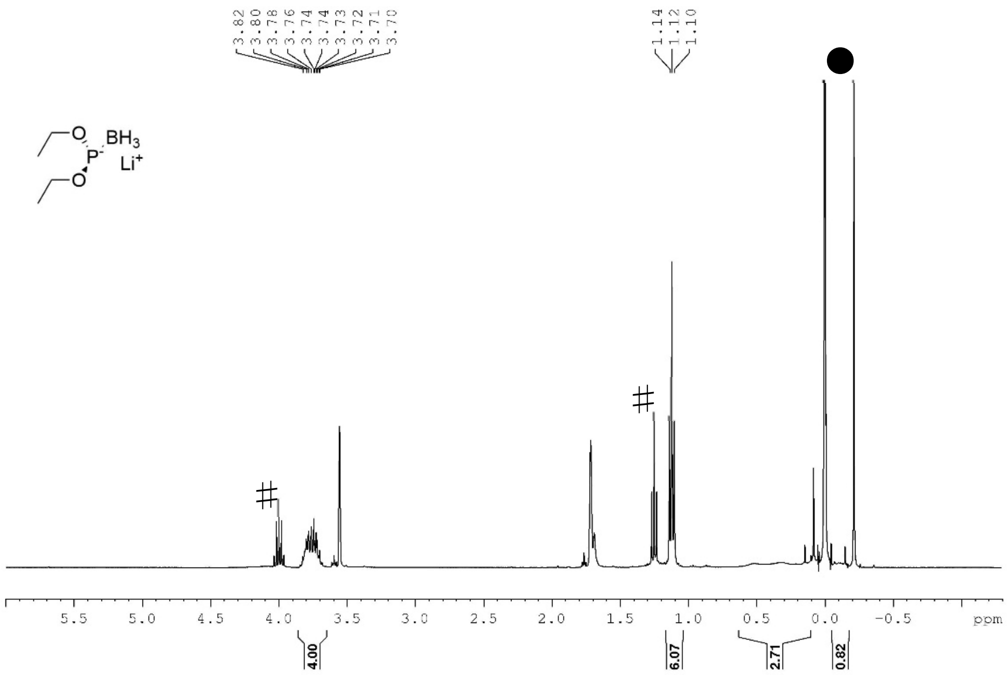

Figure S15: ^{1}H NMR spectrum of in-situ generated Li[**3b**] at 203 K in THF-d_8 (● LiHMDS/HMDS; ♯ $(EtO)_3P(BH_3)$ (impurity)).

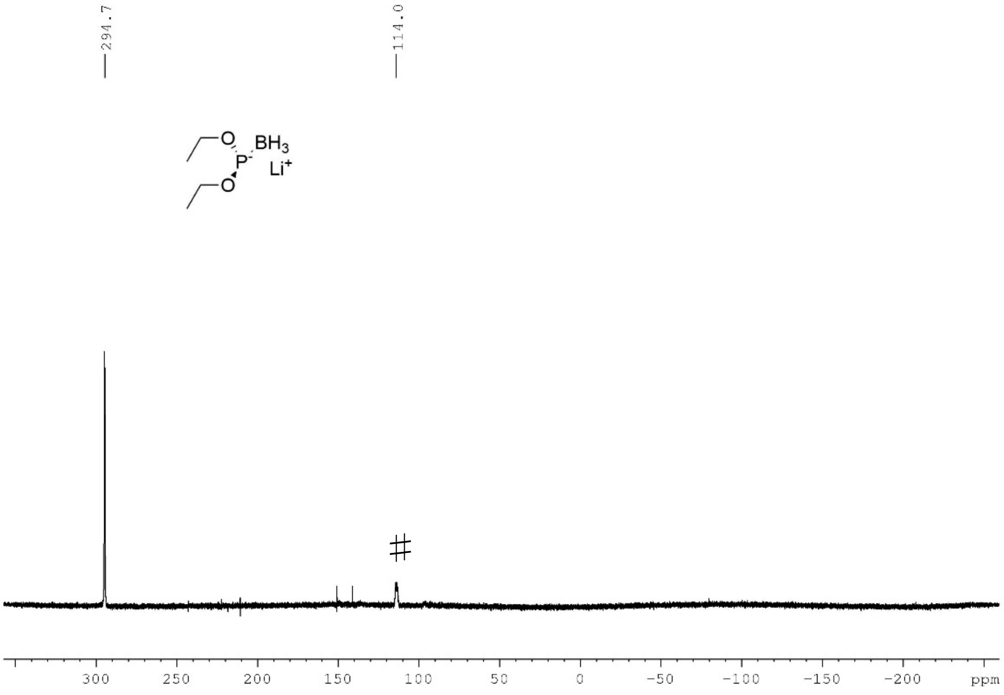

Figure S16: $^{31}P\{^{1}H\}$ NMR spectrum of in-situ generated Li[**3b**] at 203 K in THF-d_8. (♯ $(EtO)_3P(BH_3)$, impurity)

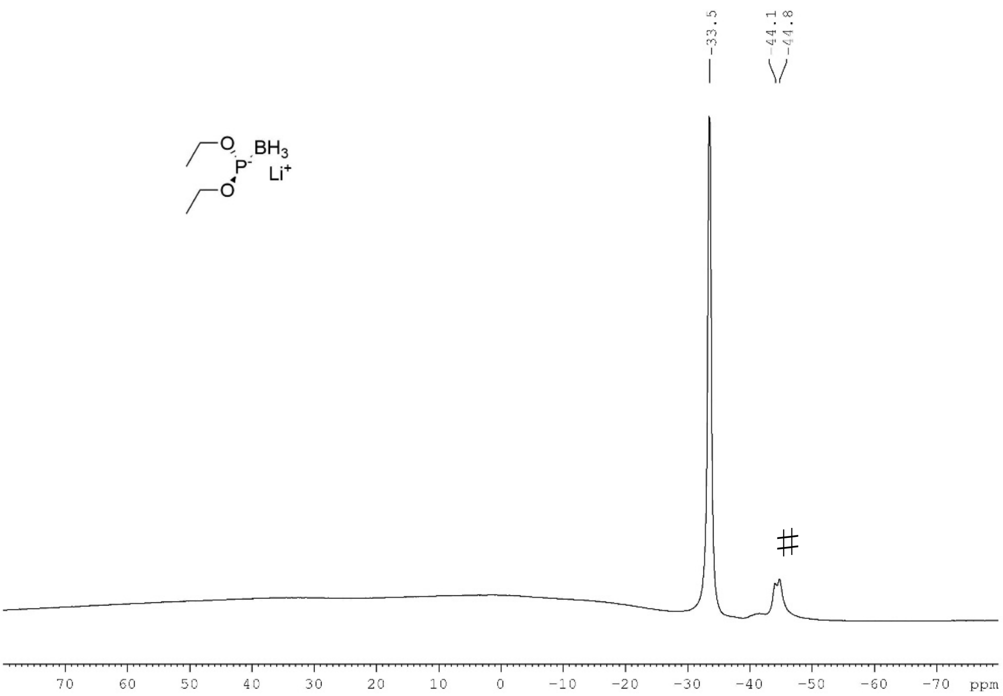

Figure S17. $^{11}B\{^1H\}$ NMR spectrum of in-situ generated Li[**3b**] at 203 K in THF-d_8 (♯ $(EtO)_3P(BH_3)$)

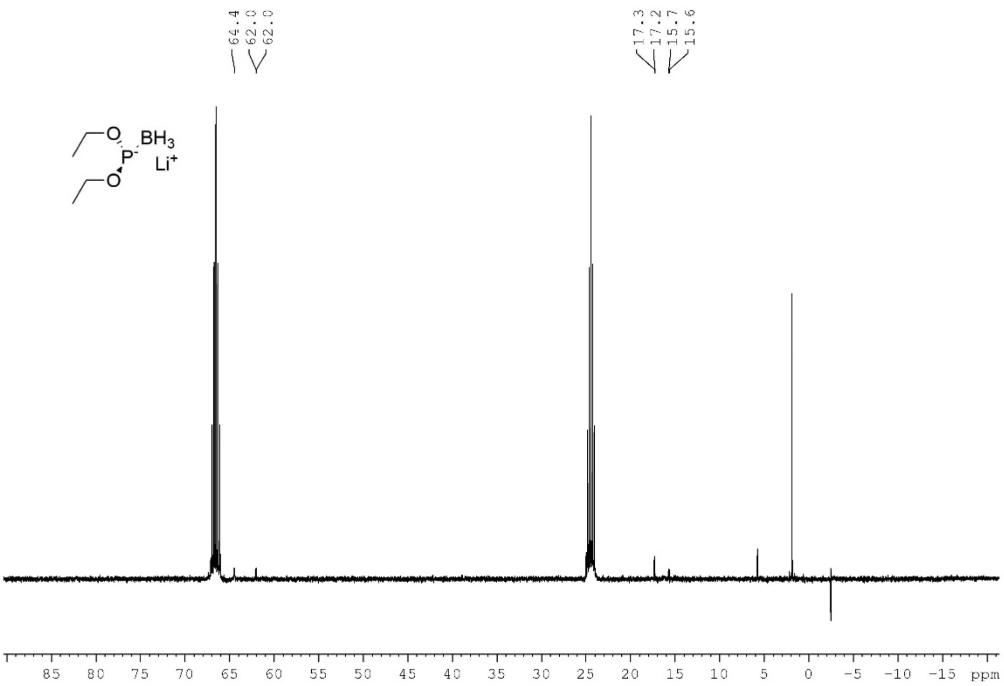

Figure S18. $^{13}C\{^1H\}$ NMR spectrum of in-situ generated Li[**3b**] at 203 K in THF-d_8.

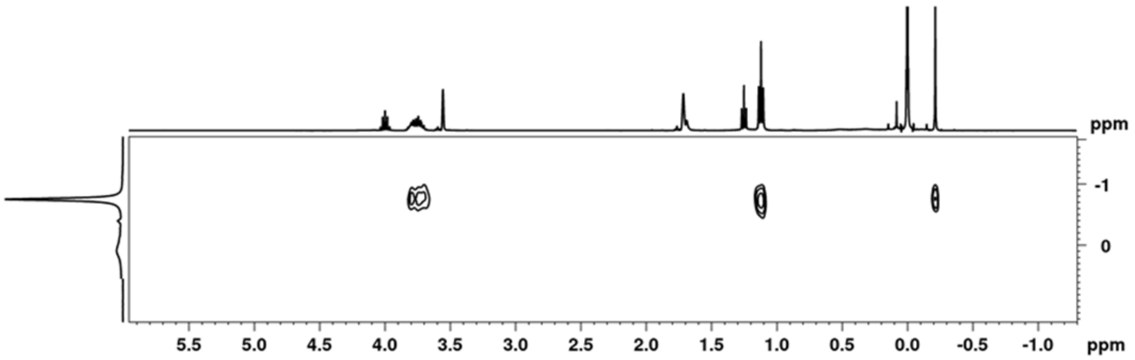

Figure S19. ^{1}H,^{7}Li gs-HOESY NMR spectrum of in-situ generated Li[**3b**] at 203 K in THF-d_8 with the ^{1}H and ^{7}Li NMR spectra as horizontal and vertical projections, respectively.

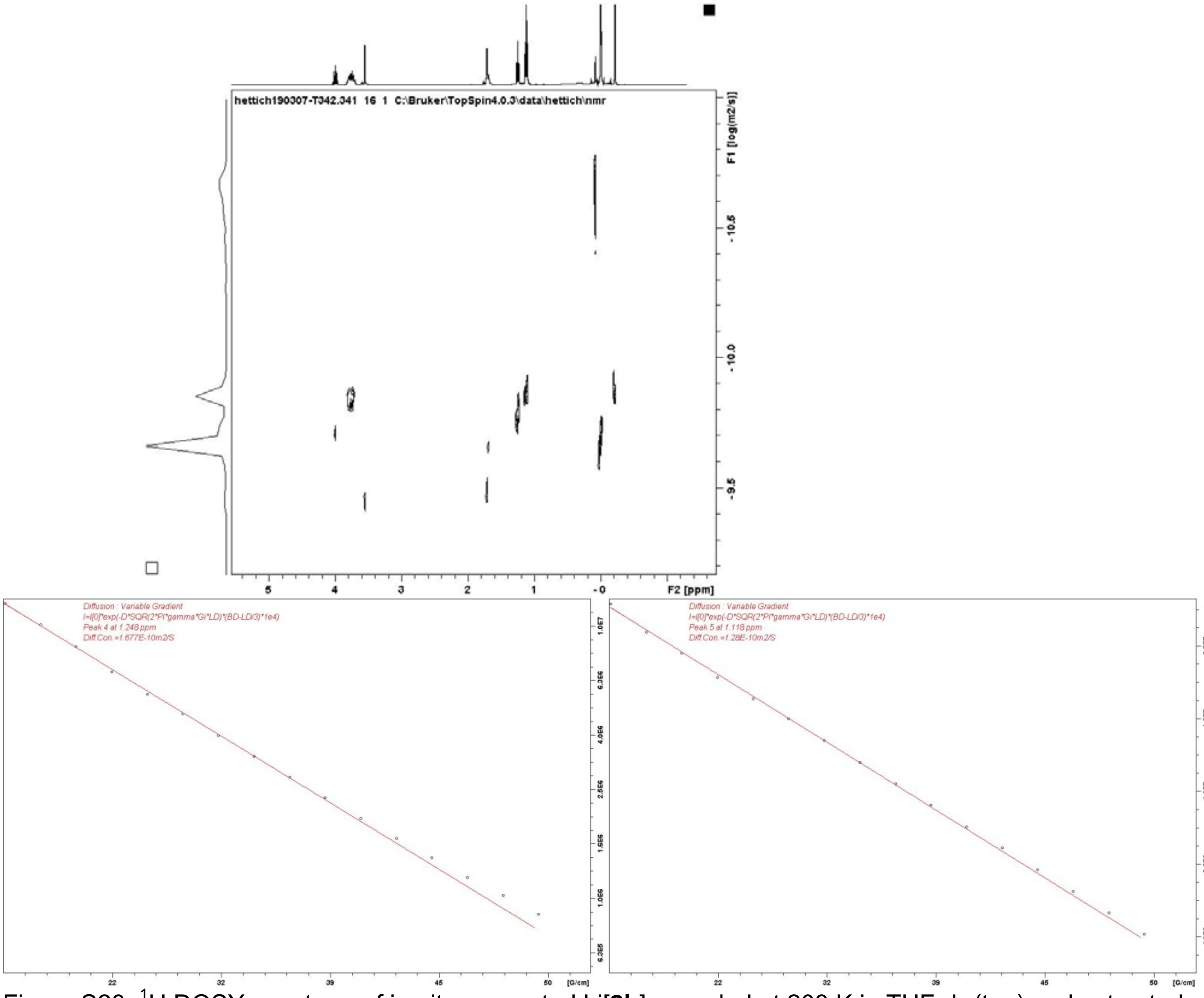

Figure S20: ^{1}H DOSY spectrum of in-situ generated Li[**3b**] recorded at 203 K in THF-d_8 (top) and extracted signal integral decays for the resonances at 1.33 ppm (bottom left) and 1.35 ppm (bottom right) attributable to the CH_3-signals of Li[**3b**] and **4**, respectively. Evaluation of the decay curves gave D(Li[**3b**]) = 1.3 m^2 s^{-1} and D(**4**) = 1.8 m^2 s^{-1}, respectively.

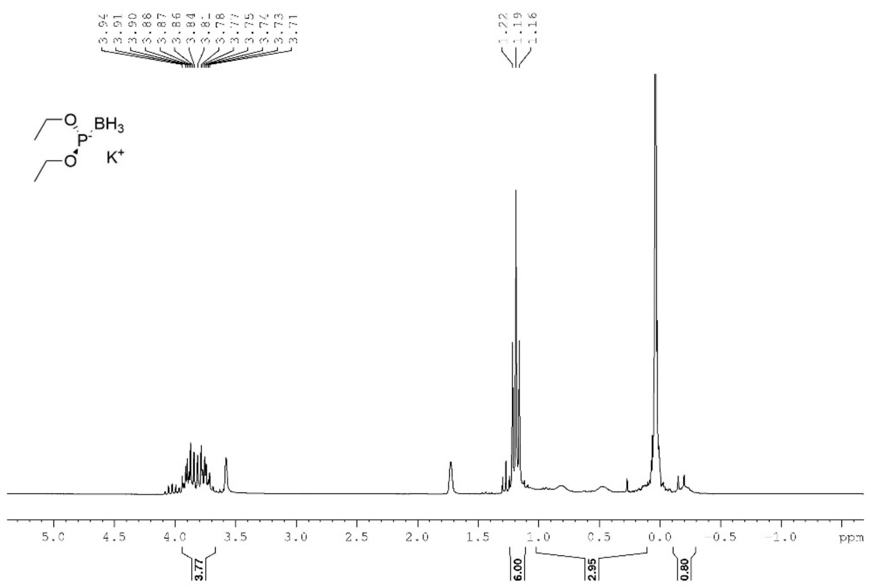

Figure S21: ^{1}H NMR spectrum of K[**3b**] in THF-d_8. The signal at 0.05 ppm is due to HMDS.

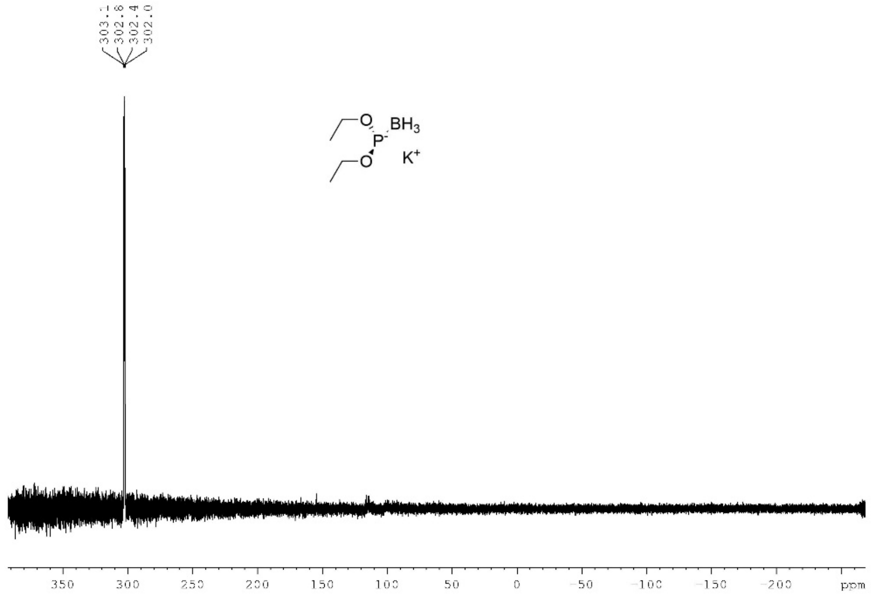

Figure S22: ^{31}P NMR spectrum of K[**3b**] in THF-d_8.

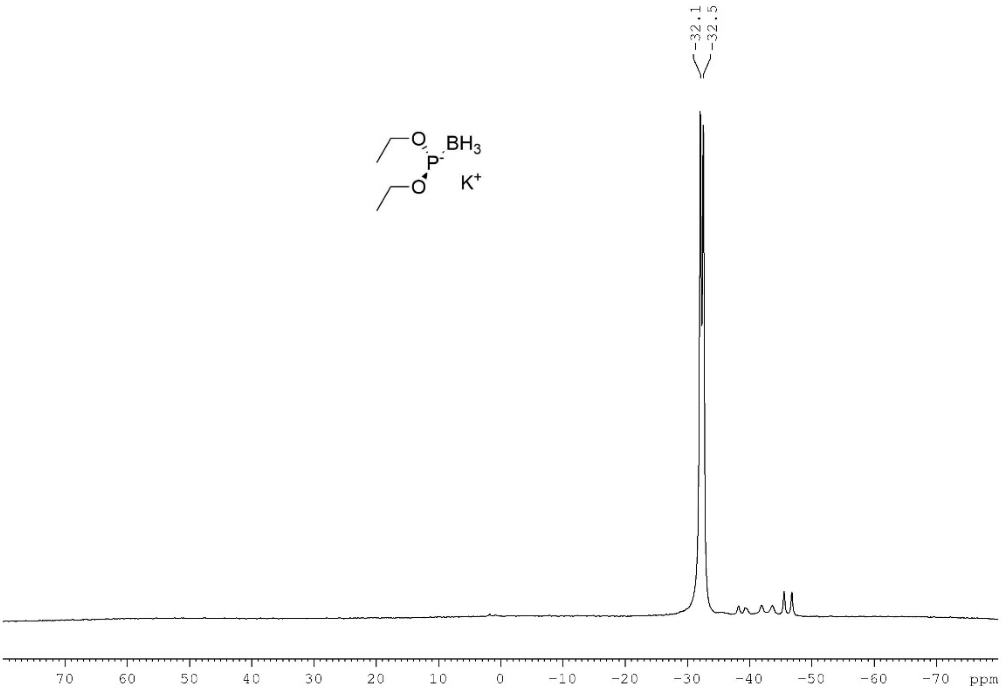

Figure S23: $^{11}B\{^{1}H\}$ NMR spectrum of K[**3b**] in THF-d_8.

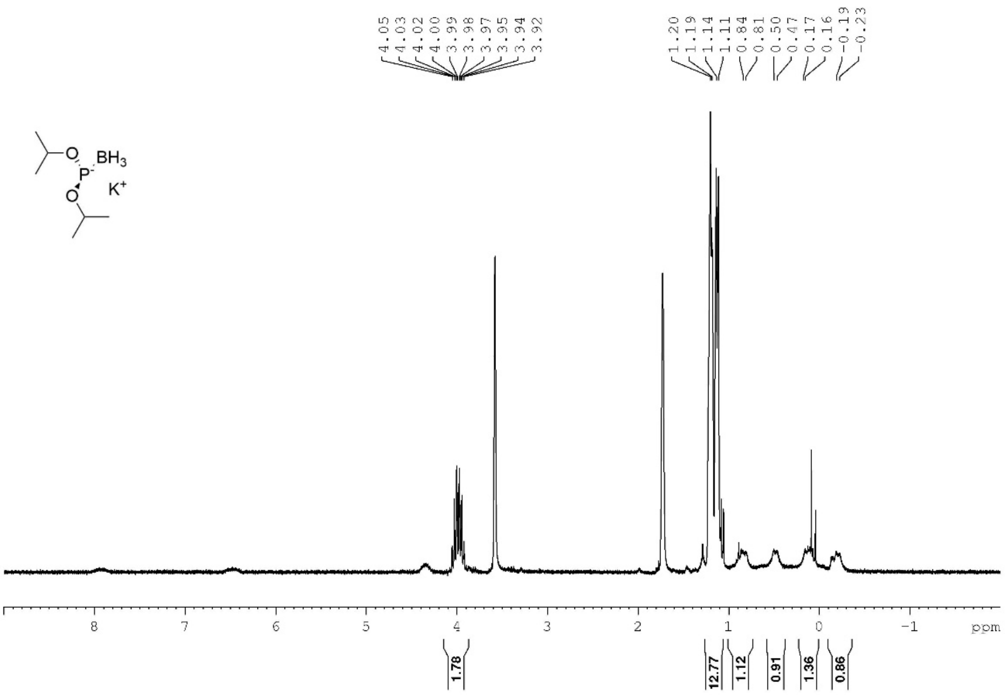

Figure S24: ^{1}H NMR spectrum of K[**3c**] in THF-d_8.

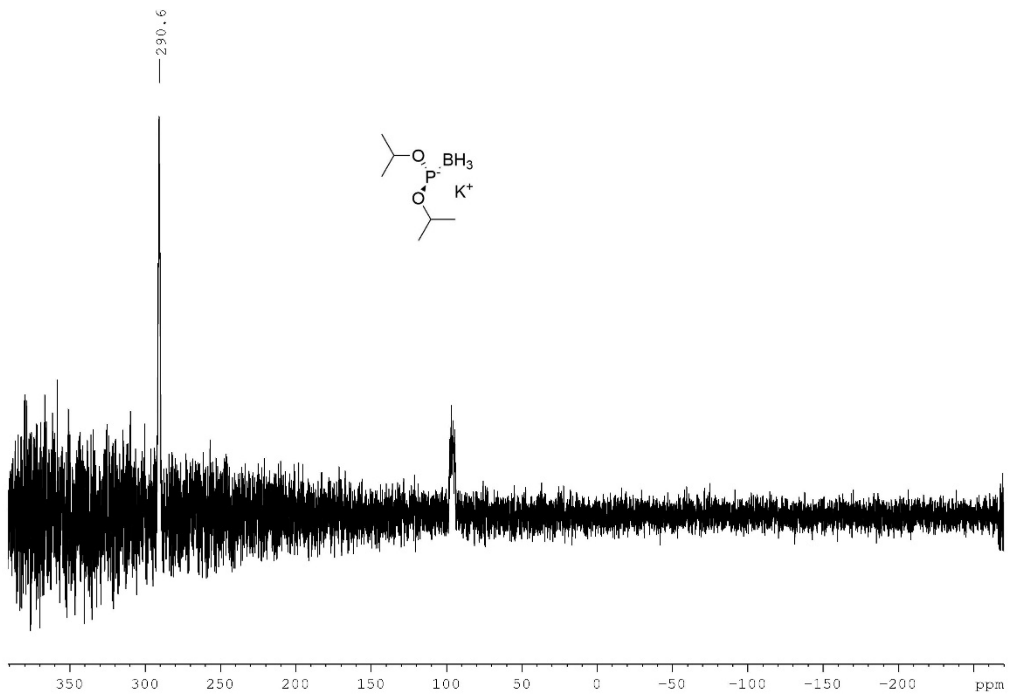

Figure S25: ^{31}P NMR spectrum of K[**3c**] in THF-d_8. The signal at 99 ppm is assigned to K[(iPrO)$_2$PO(BH_3)].

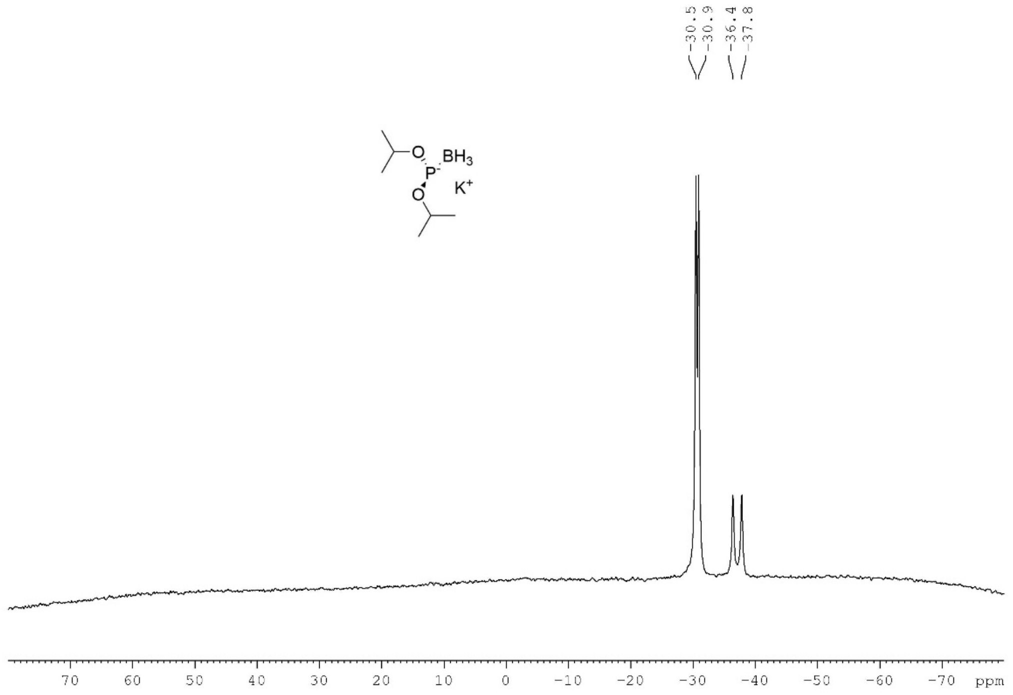

Figure S26: $^{11}B\{^1H\}$ NMR spectrum of K[**3c**] in THF-d_8. The signal at -37 ppm is assigned to K[(iPrO)$_2$PO(BH_3)].

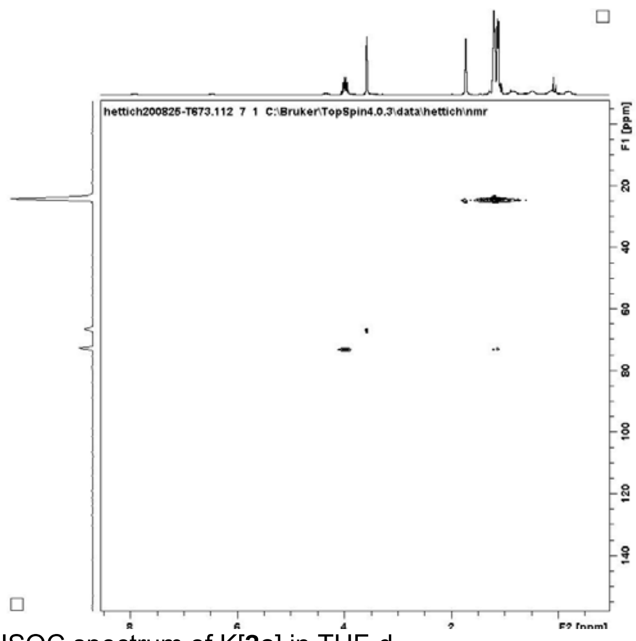

Figure S27: ^{1}H-^{13}C-HSQC spectrum of K[**3c**] in THF-d_8.

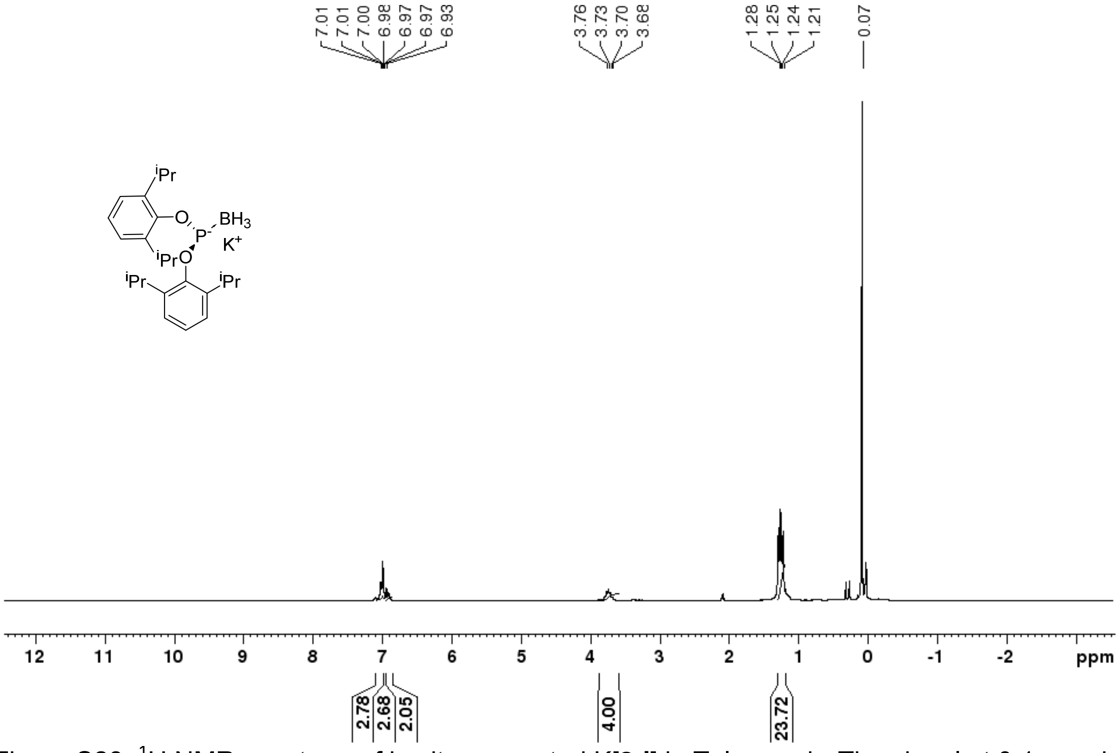

Figure S28: ^{1}H NMR spectrum of in situ generated K[**3d**] in Toluene-d_8. The signal at 0.1 ppm is due to HMDS.

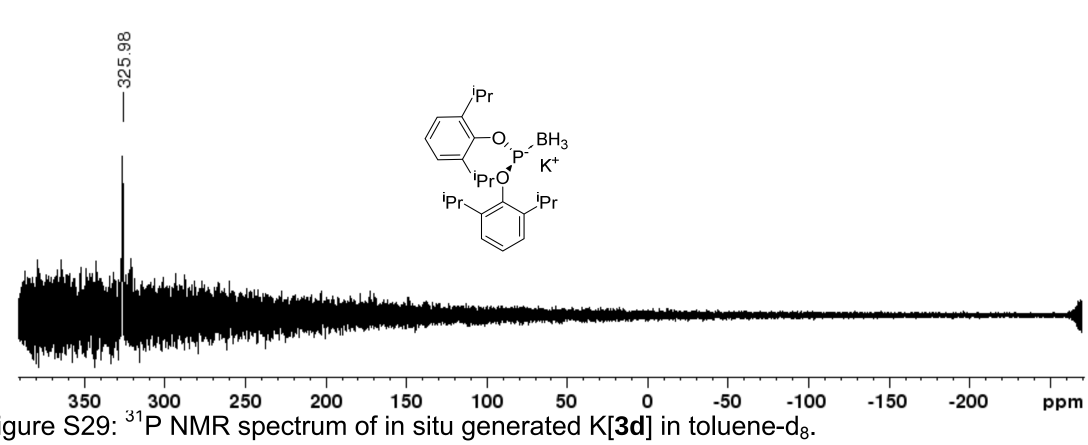

Figure S29: ^{31}P NMR spectrum of in situ generated K[**3d**] in toluene-d_8.

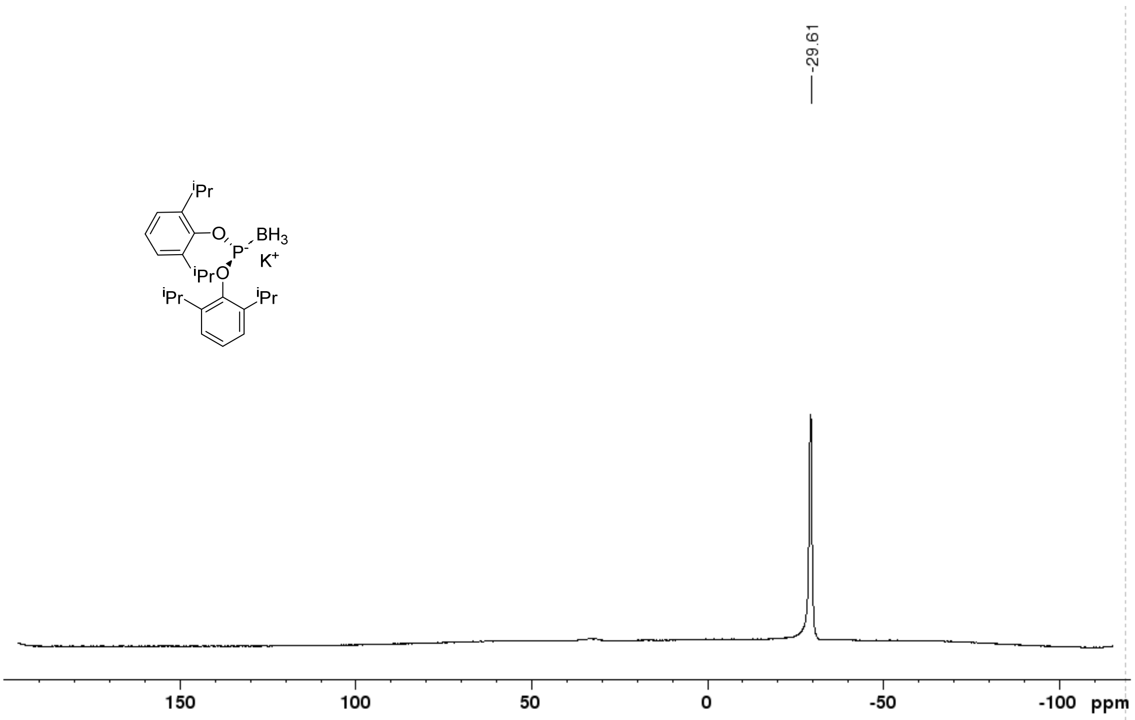

Figure S30: $^{11}B\{^1H\}$ NMR spectrum of in situ generated K[**3d**] in toluene-d_8.

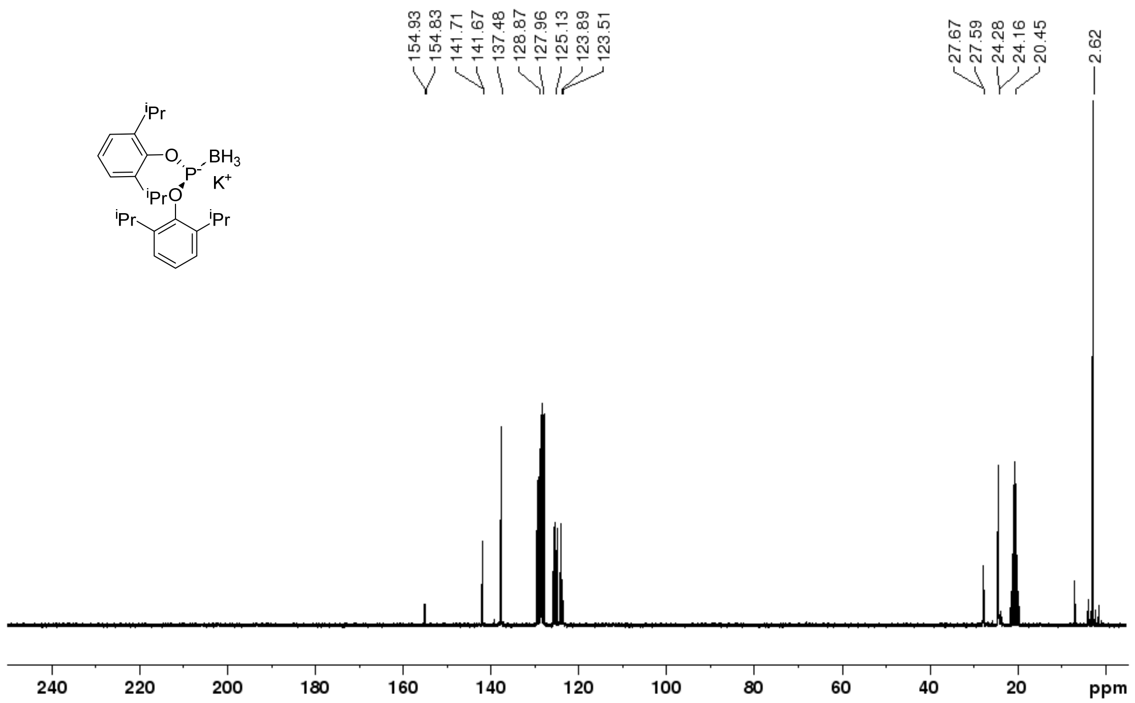

Figure S31: $^{13}C\{^1H\}$ NMR spectrum of in situ generated K[**3d**] in toluene-d_8.The signal at 2.6 ppm is due to HMDS.

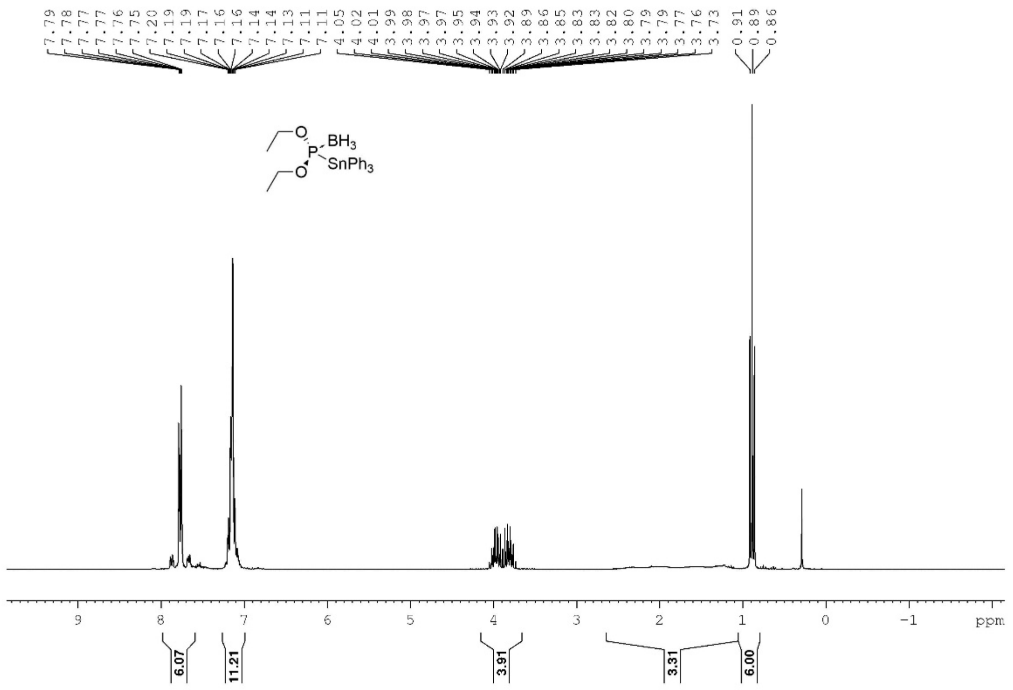

Figure S32: ^{1}H NMR spectrum of **9b** in C_6D_6.

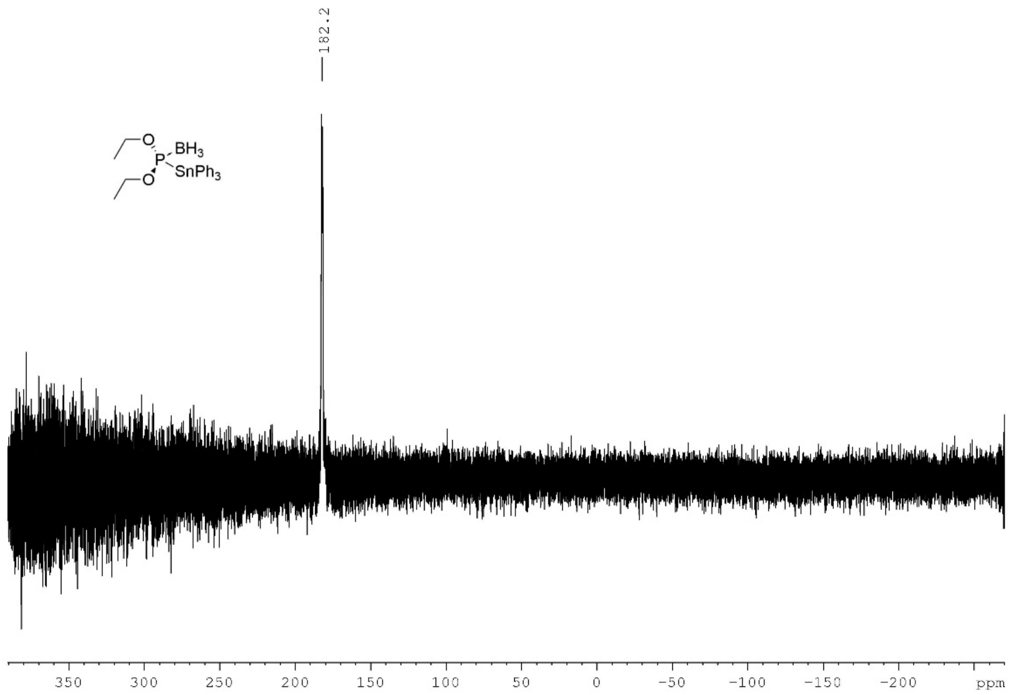

Figure S33: $^{31}P\{^{1}H\}$ NMR spectrum of **9b** in C_6D_6.

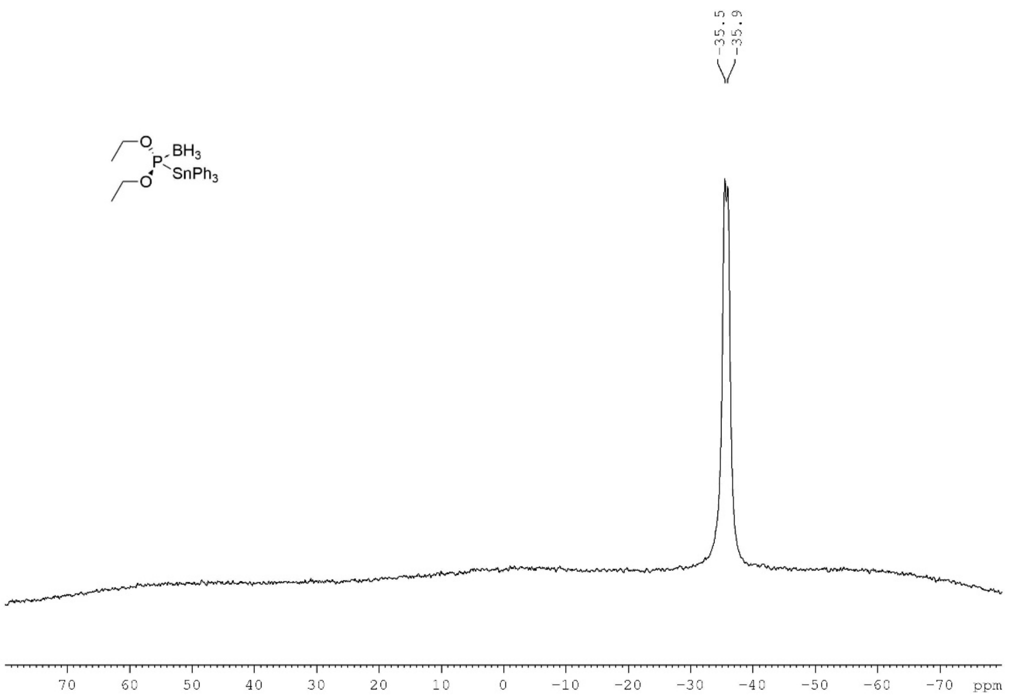

Figure S34: $^{11}B\{^{1}H\}$ NMR-spectrum of **9b** in C_6D_6.

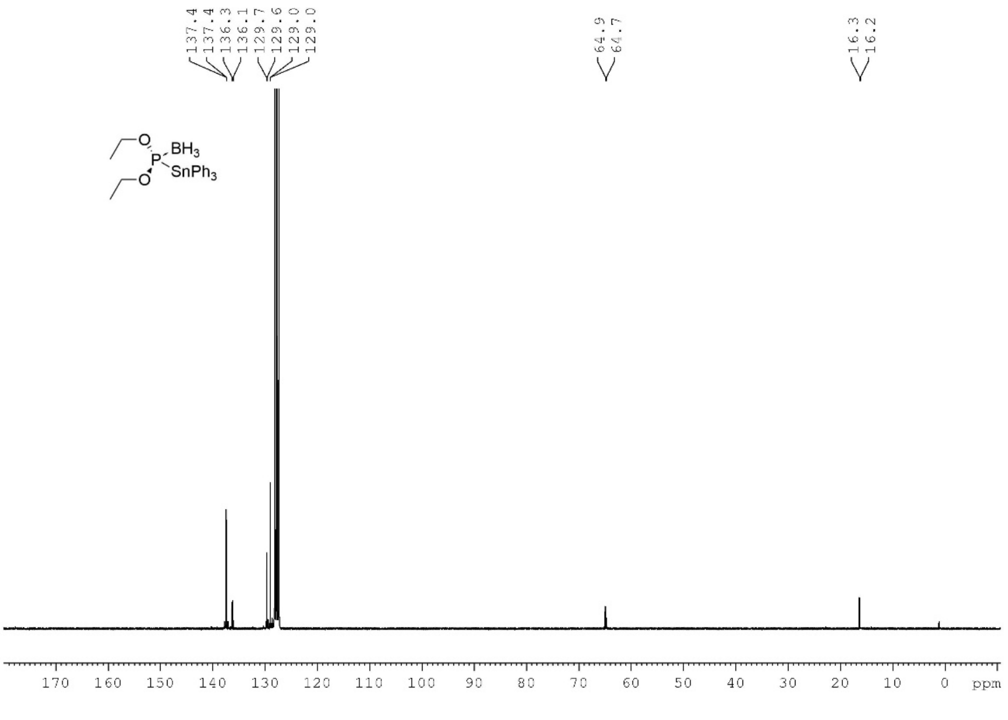

Figure S35: ^{13}C NMR-spectrum of **9b** in C_6D_6.

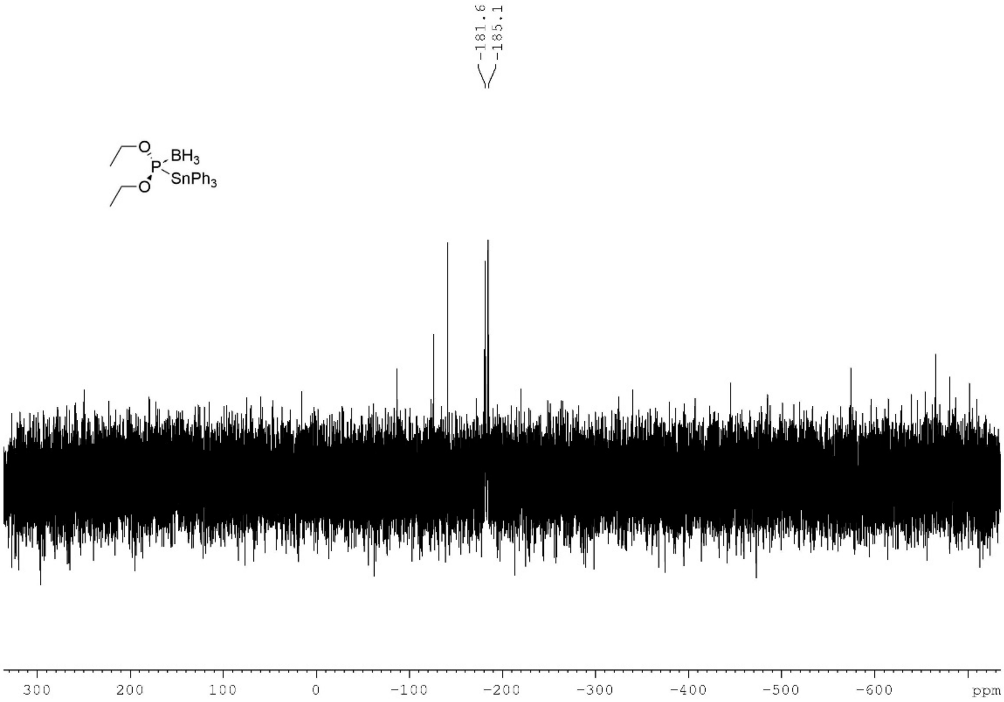

Figure S36: ^{119}Sn-DEPT spectrum of **9b** in C_6D_6.

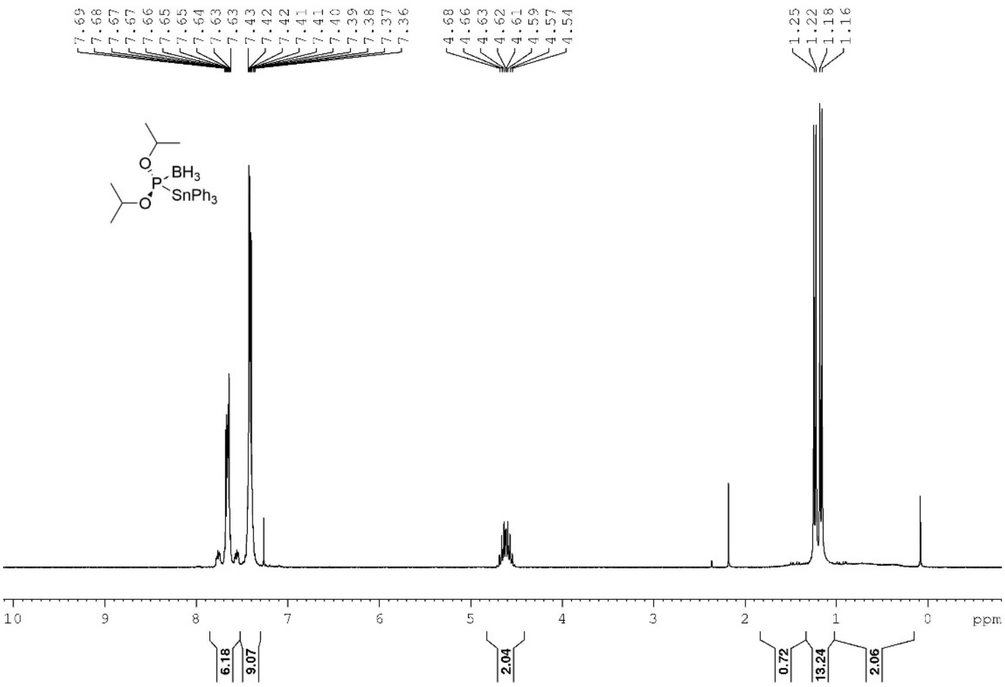

Figure S37: ^{1}H NMR spectrum of **9c** in $CDCl_3$.

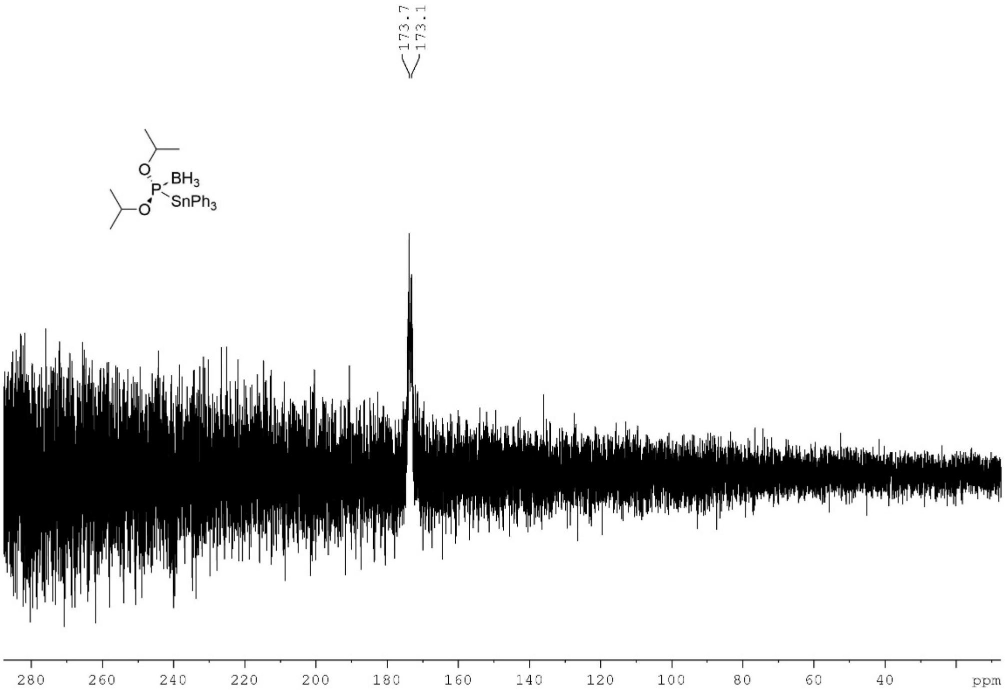

Figure S38: $^{31}P\{^1H\}$ NMR spectrum of **9c** in $CDCl_3$.

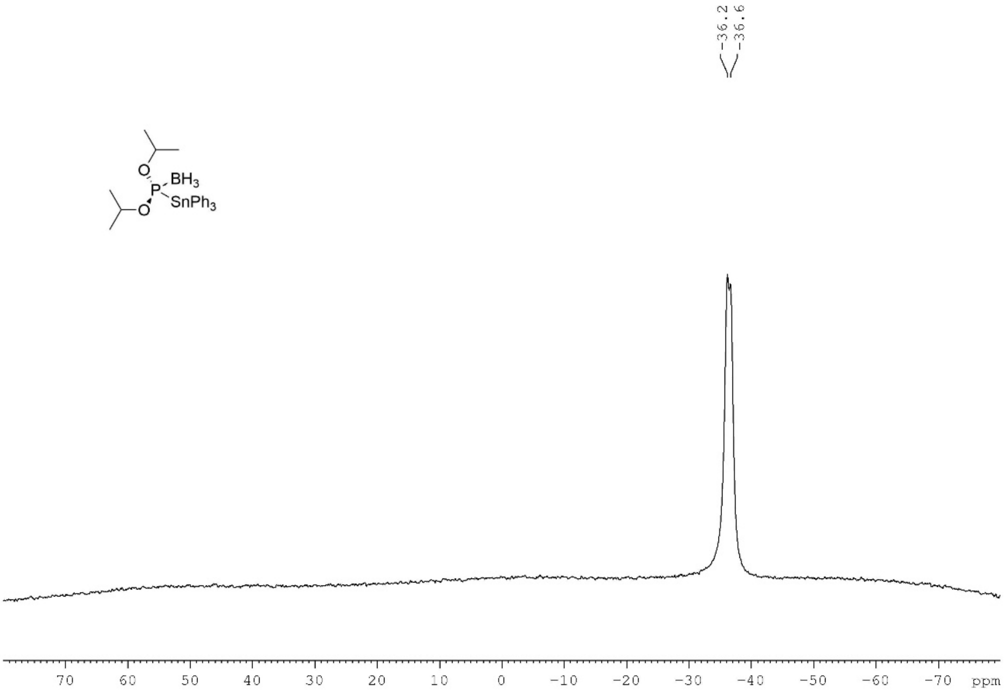

Figure S39: $^{11}B\{^1H\}$ NMR-spectrum of **9c** in $CDCl_3$.

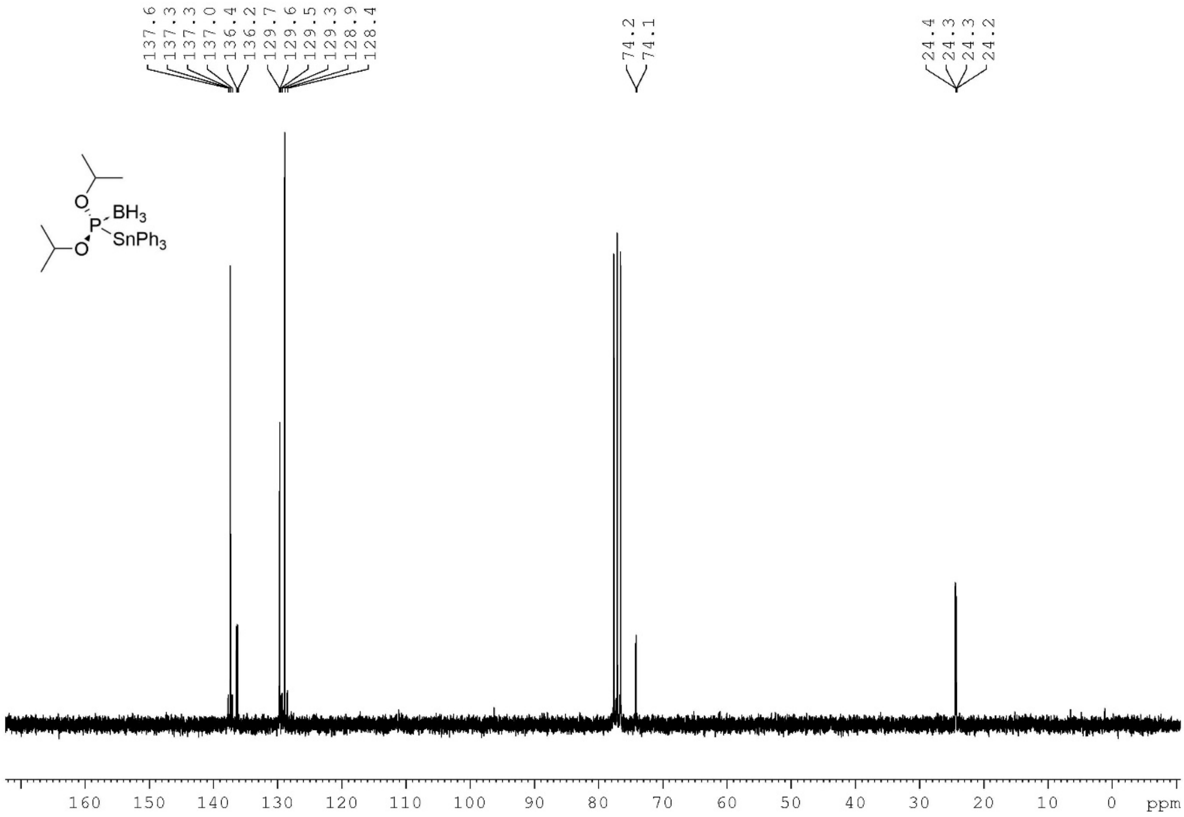

Figure S40: $^{13}C\{^{1}H\}$ NMR spectrum of **9c** in $CDCl_3$.

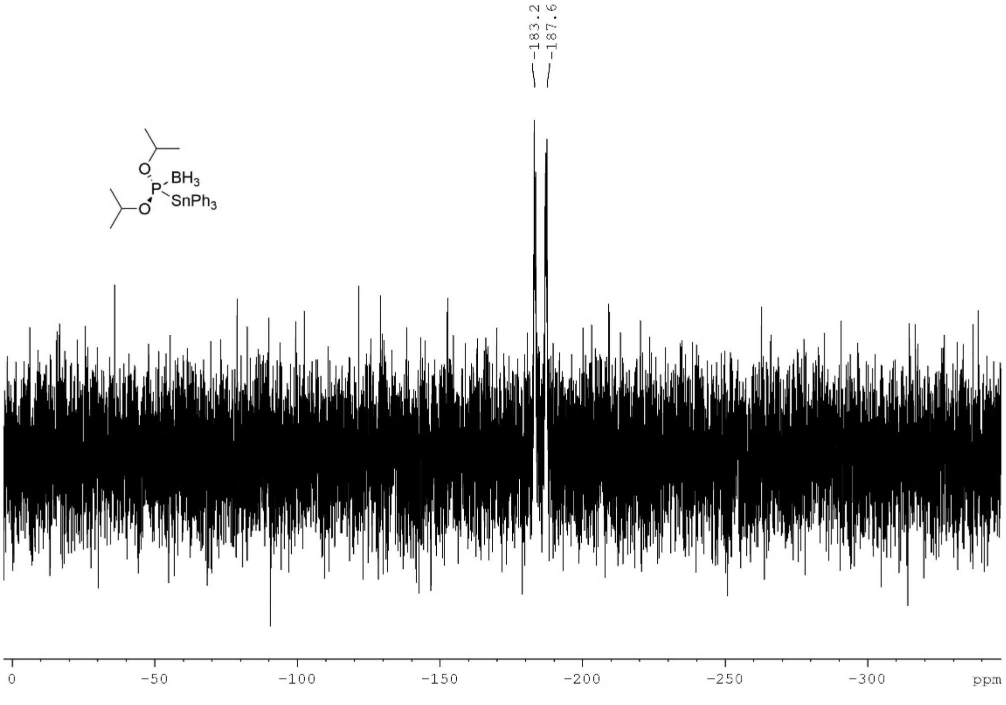

Figure S41: ^{119}Sn-DEPT spectrum of **9c** in $CDCl_3$.

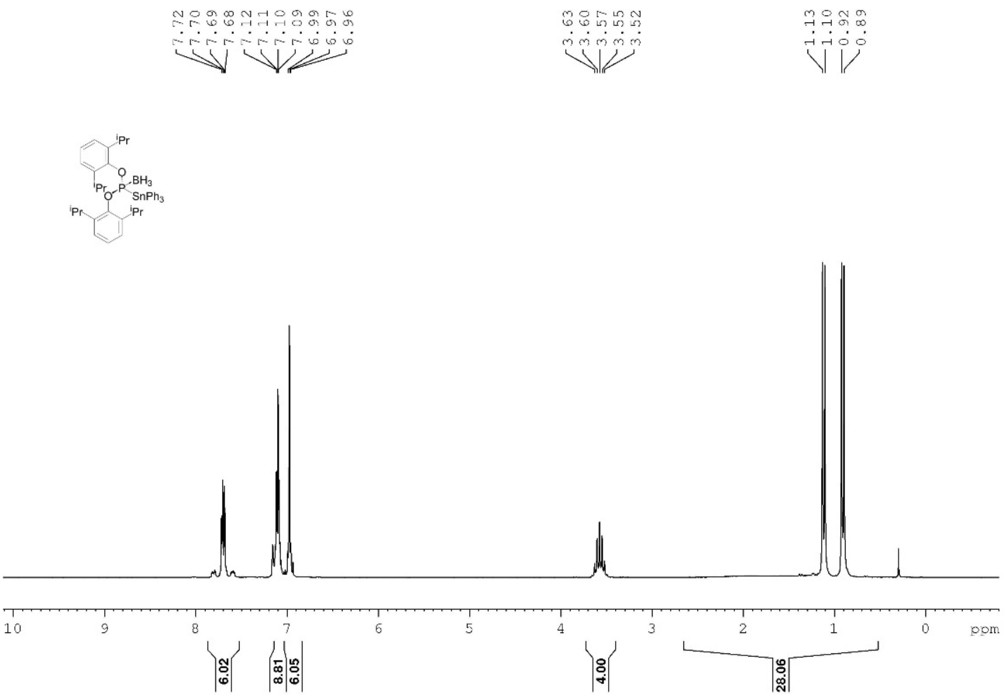

Figure S42: ^{1}H NMR spectrum of **9d** in C_6D_6.

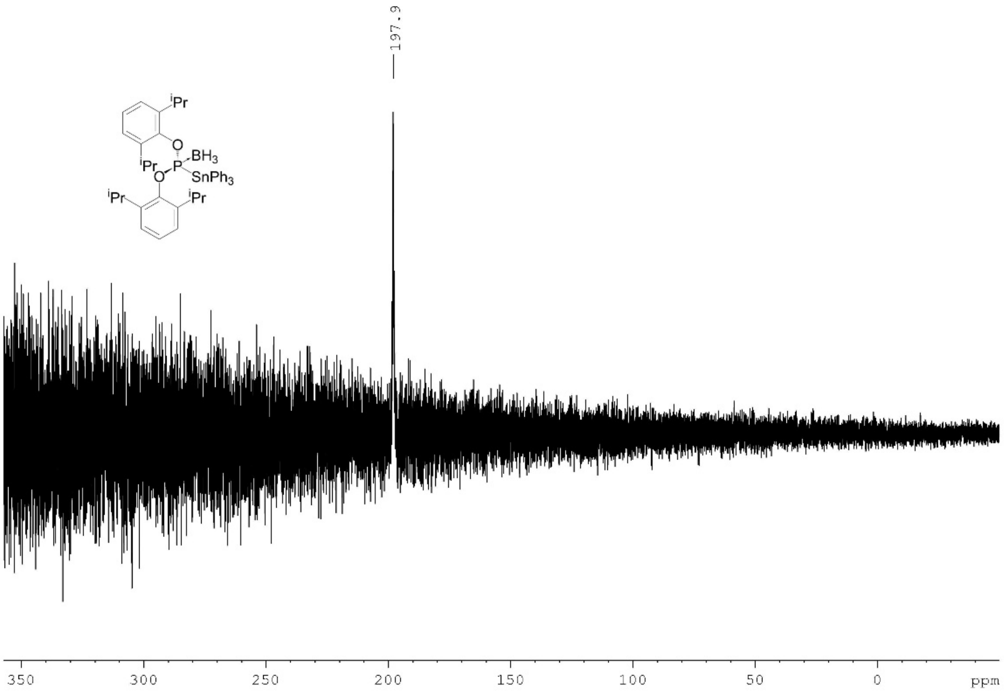

Figure S43: ^{31}P{^{1}H} NMR spectrum of **9d** in C_6D_6.

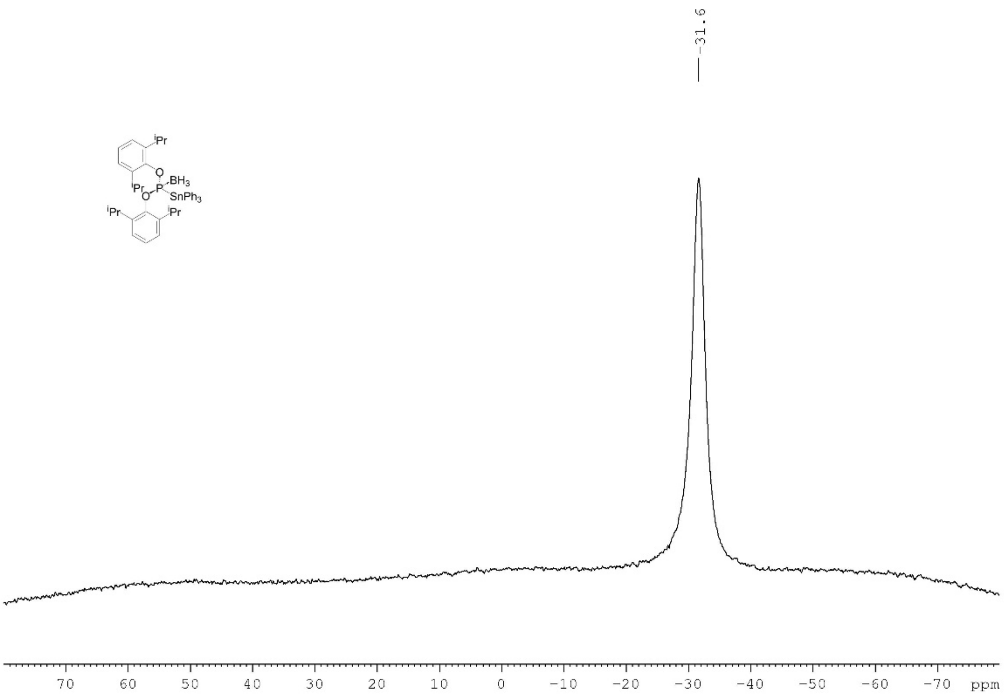

Figure S44: $^{11}B\{^{1}H\}$ NMR spectrum of **9d** in C_6D_6.

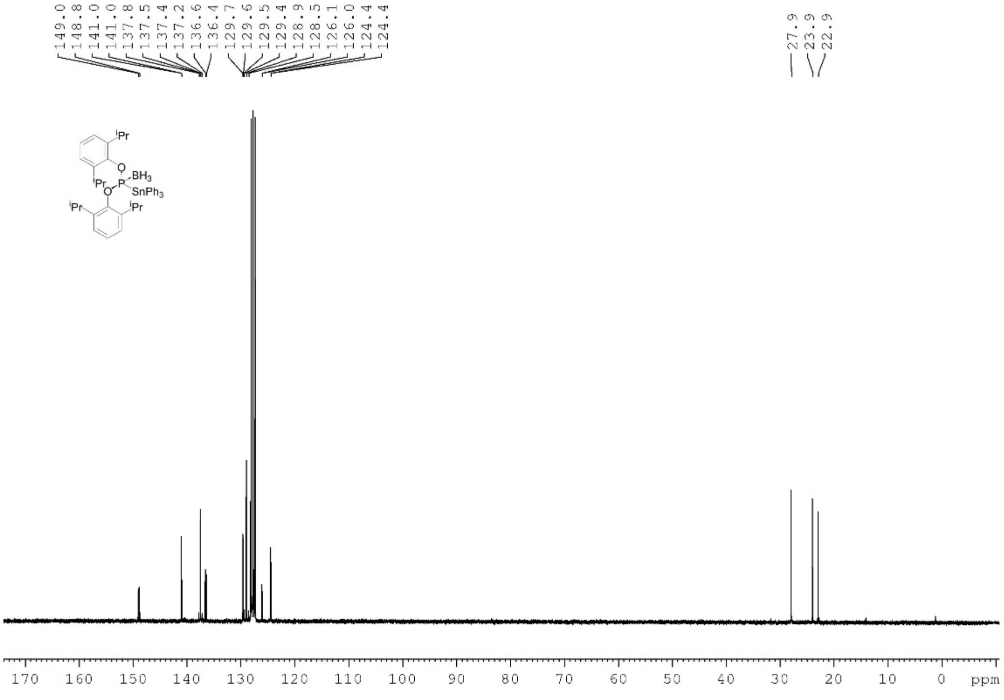

Figure S45: $^{13}C\{^{1}H\}$ NMR spectrum of **9d** in C_6D_6.

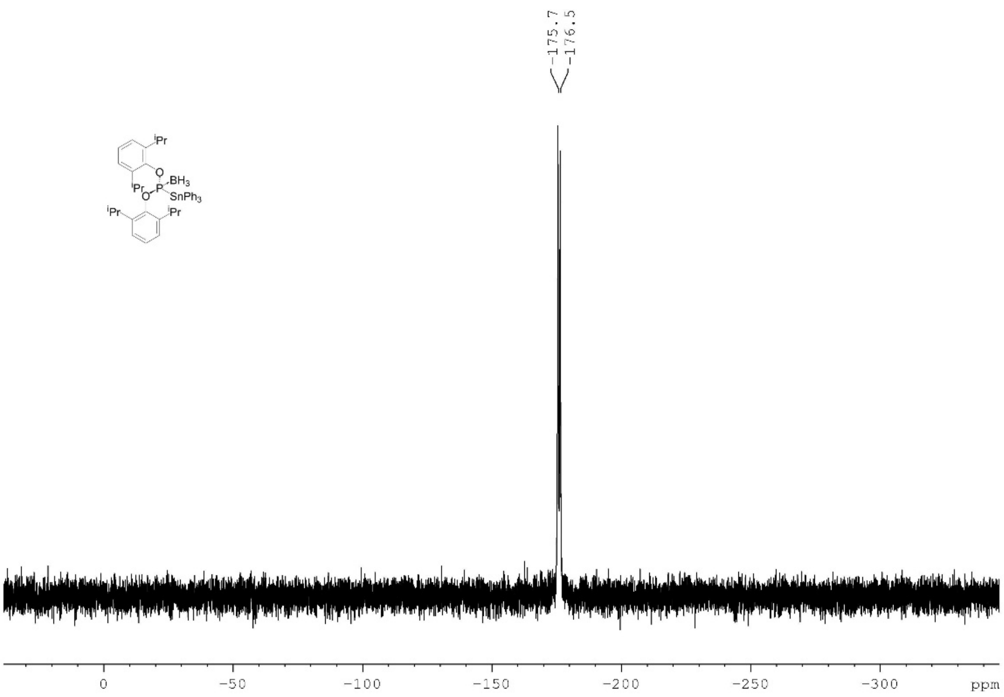

Figure S46: ^{119}Sn-DEPT spectrum of **9d** in C_6D_6.

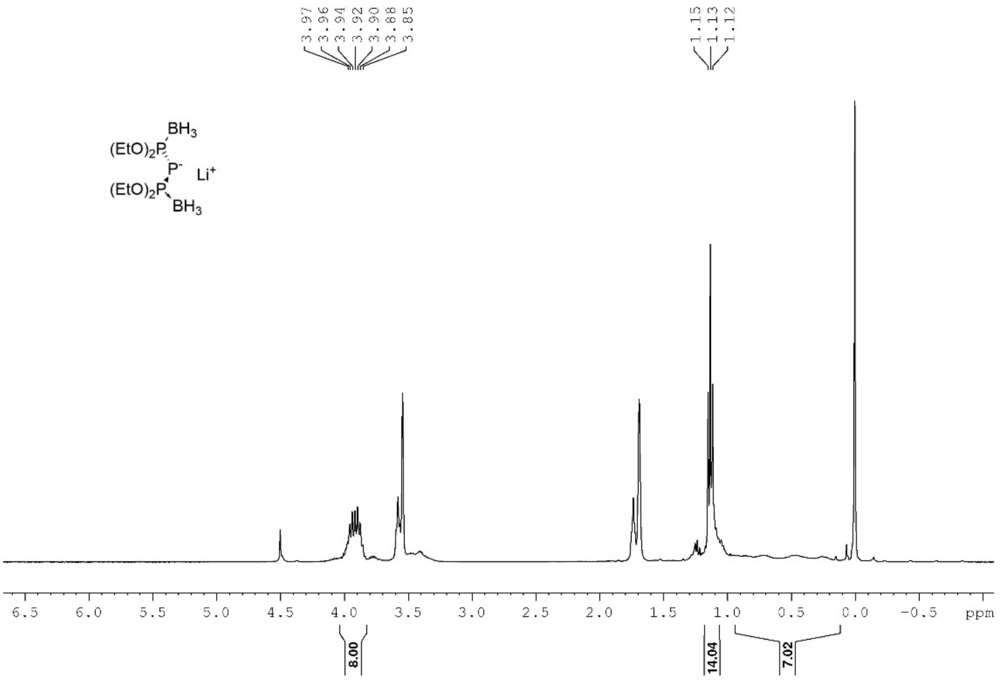

Figure S47: ^{1}H NMR spectrum of Li[**6b**] in THF-d_8.

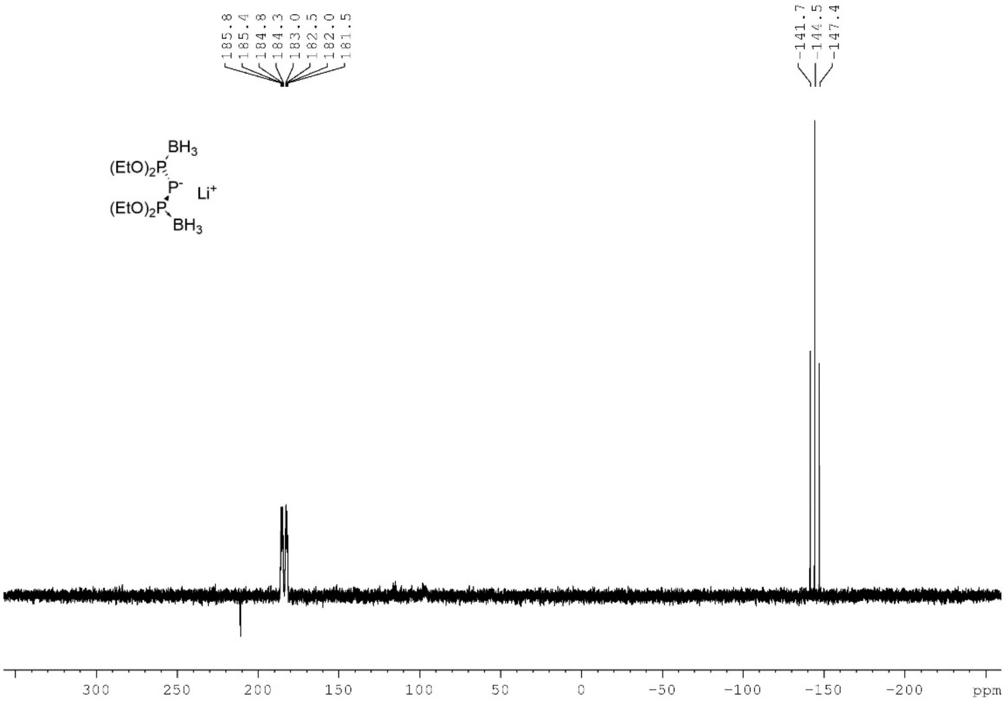

Figure S48: $^{31}P\{^{1}H\}$ NMR spectrum of Li[**6b**] in THF-d_8.

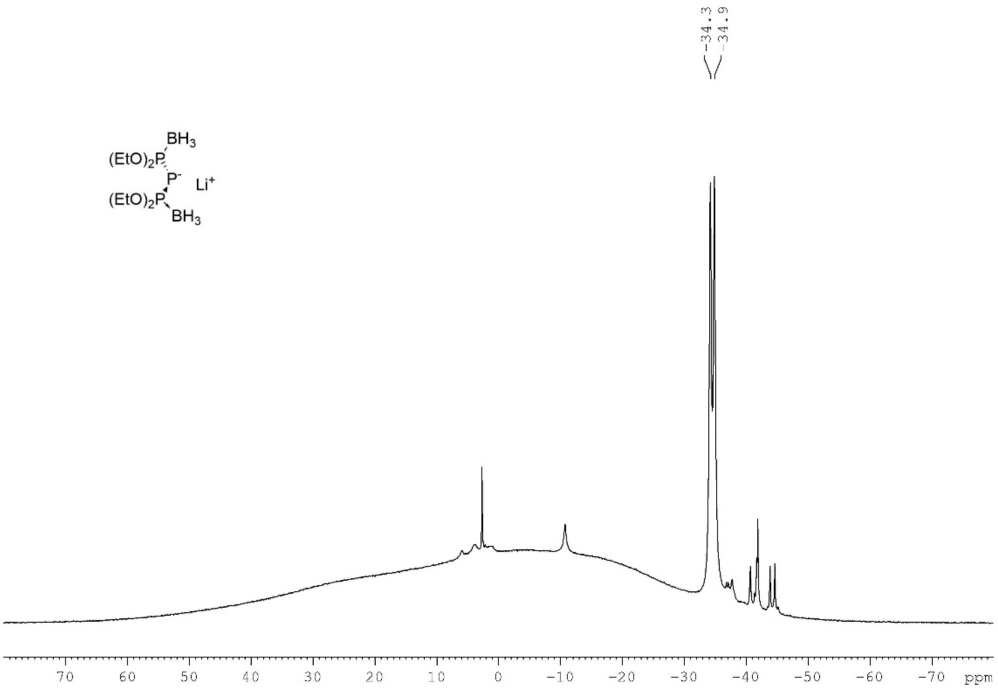

Figure S49: $^{11}B\{^{1}H\}$ NMR spectrum of Li[**6b**] in THF-d_8.

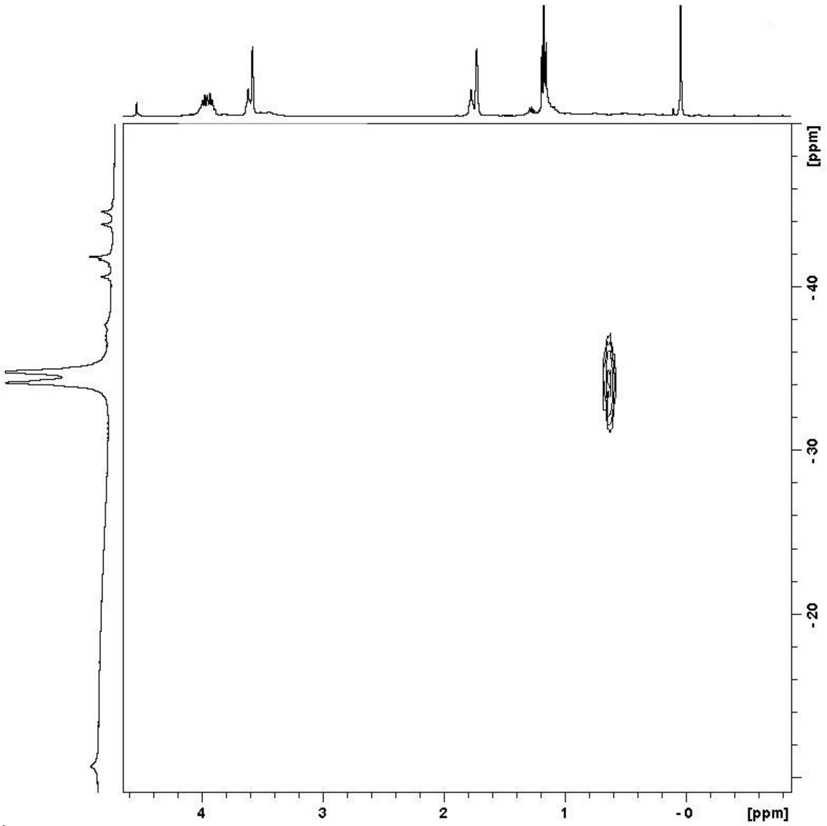

Figure S50: $^{1}H,^{11}B$ HSQC spectrum of Li[**6b**] in THF-d_8.

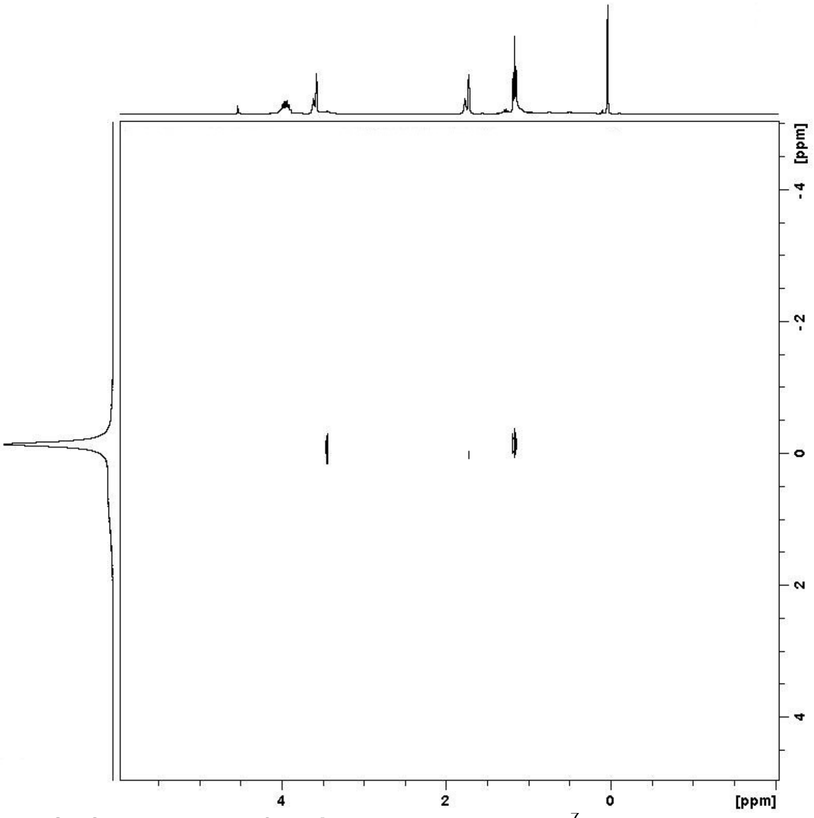

Figure S51: $^{1}H,^{7}Li$ HOESY spectrum of Li[**6b**] in THF-d_8 with the ^{7}Li NMR spectrum as vertical projection.

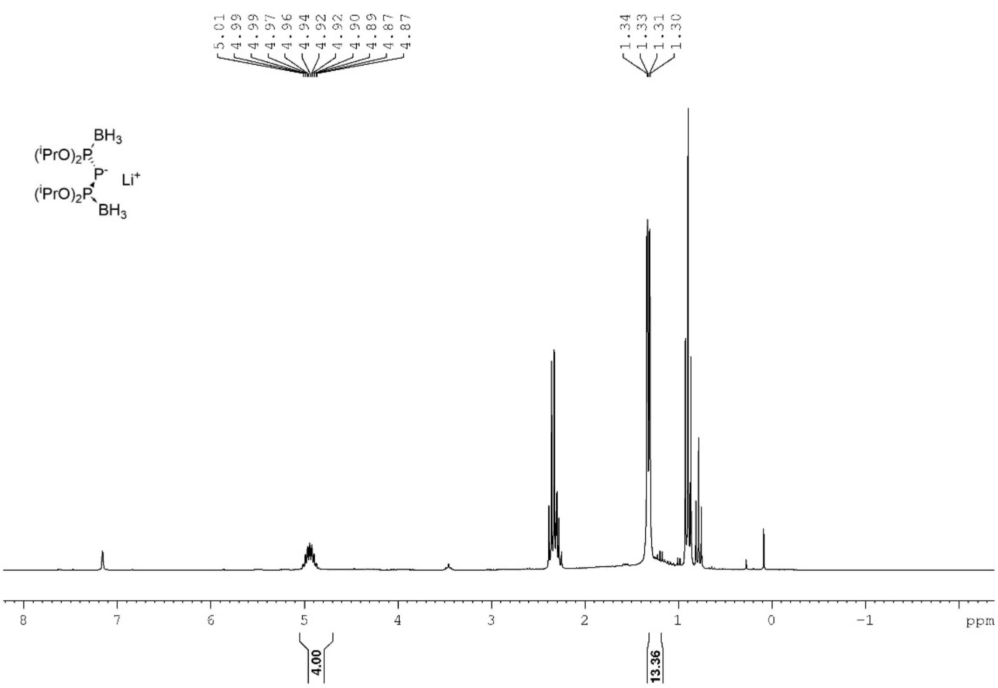

Figure S52: ^{1}H NMR spectrum of Li[**6c**] in C_6D_6.

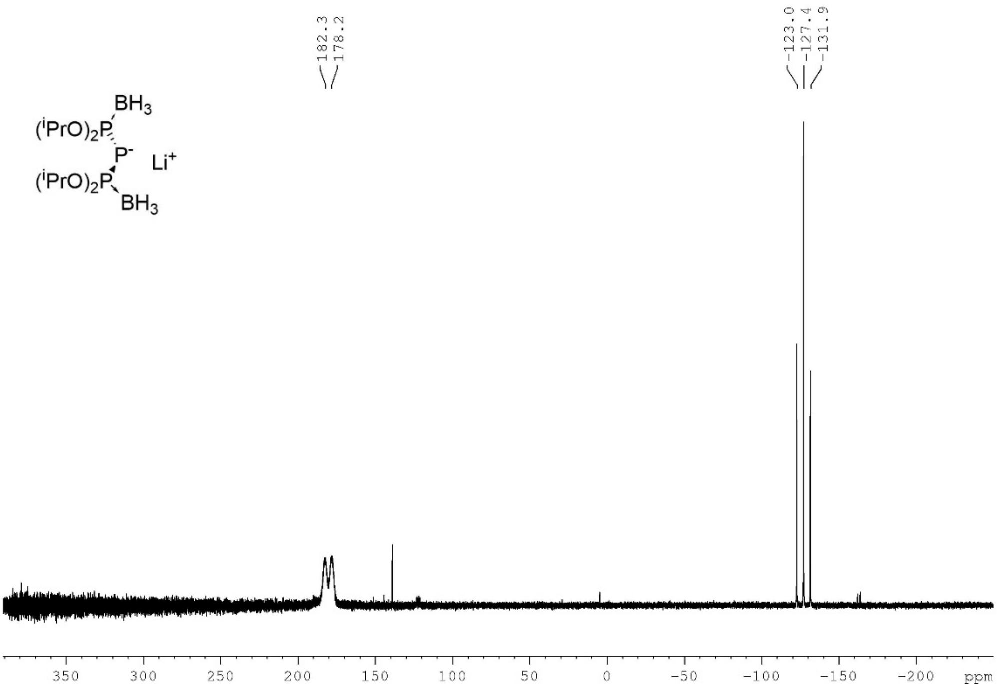

Figure S53: $^{31}P\{^{1}H\}$ NMR spectrum of Li[**6c**] in C_6D_6.

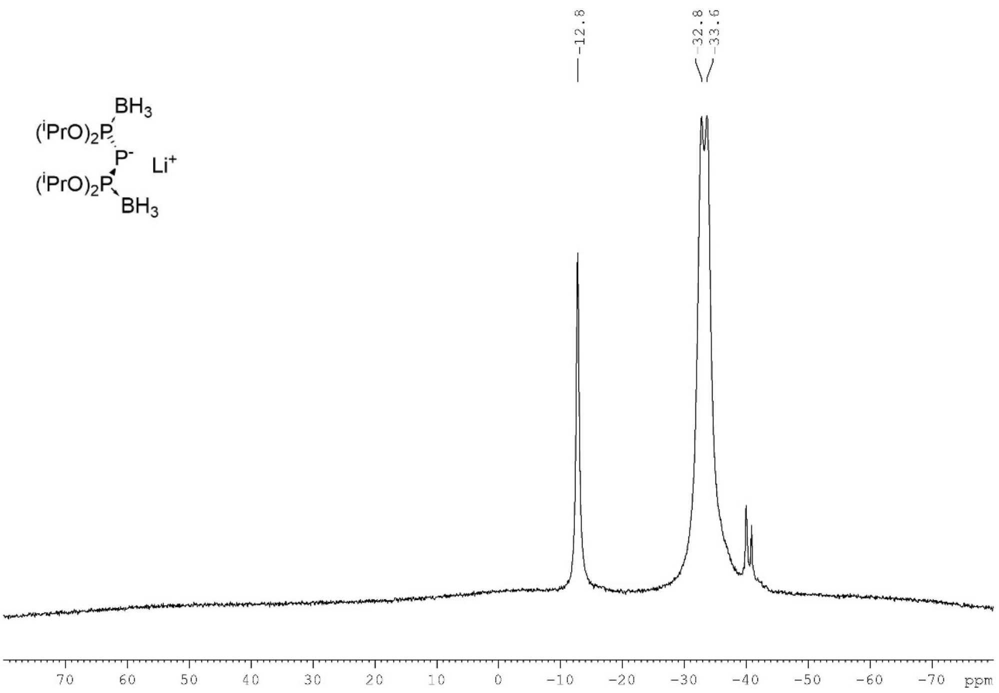

Figure S54: $^{11}B\{^{1}H\}$ NMR spectrum of Li[**6c**] in C_6D_6.

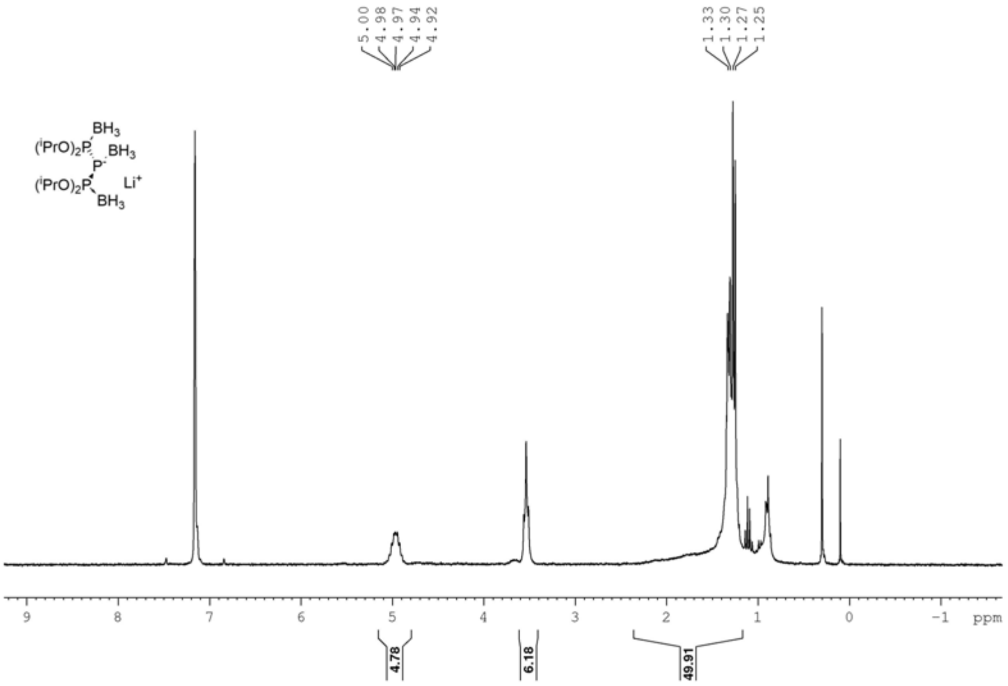

Figure S55: ^{1}H NMR spectrum of Li[**5c**] in C_6D_6.

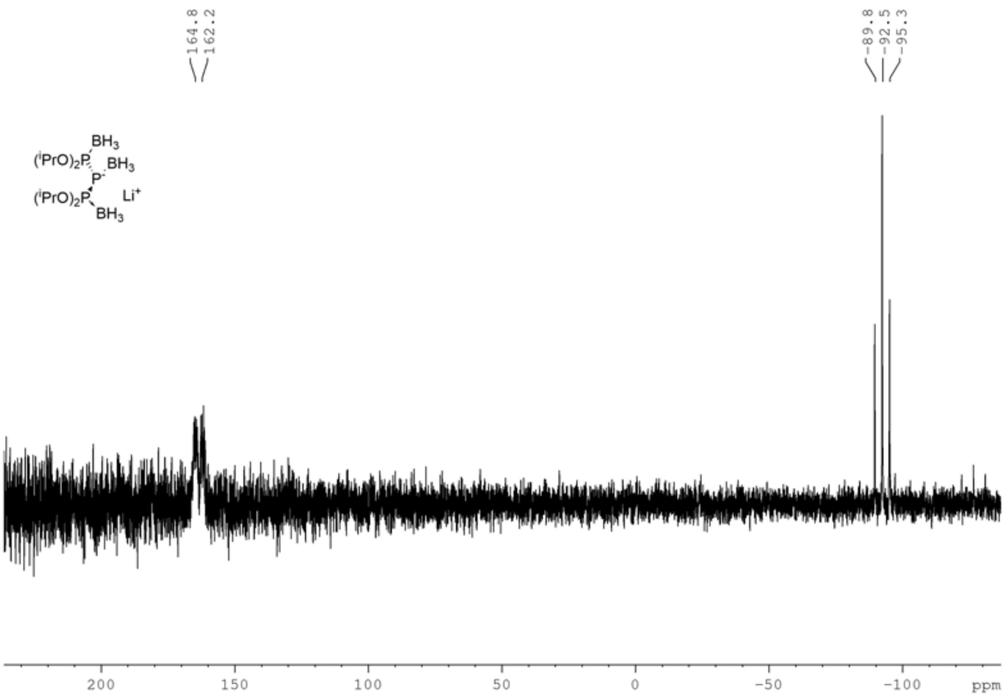

Figure S56: $^{31}P\{^{1}H\}$ NMR spectrum of Li[**5c**] in C_6D_6.

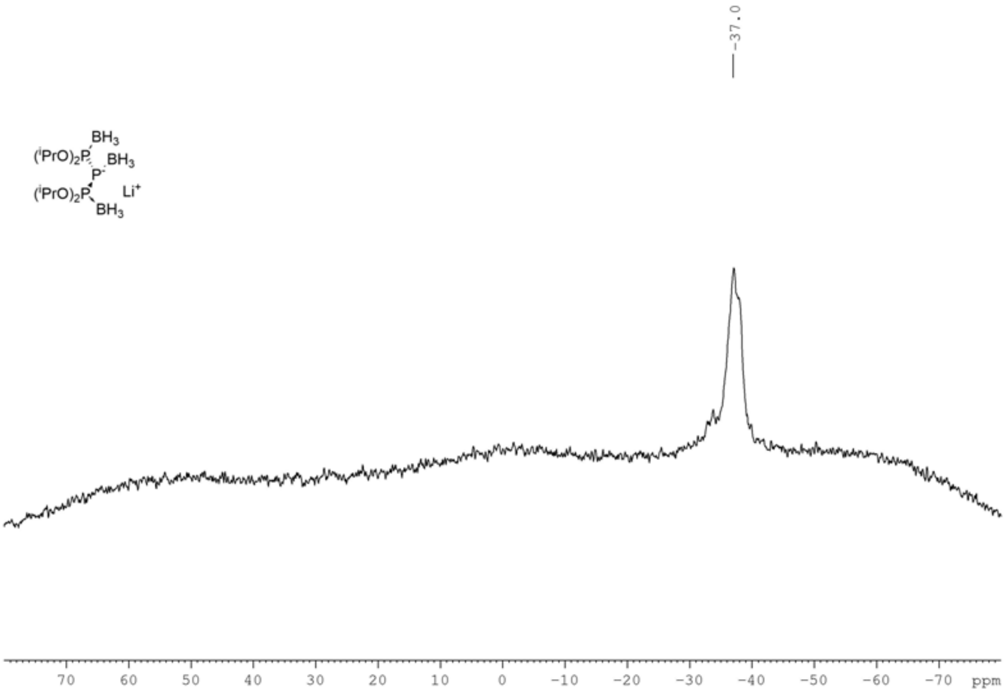

Figure S57: $^{11}B\{^{1}H\}$ NMR spectrum of Li[**5c**] in C_6D_6.

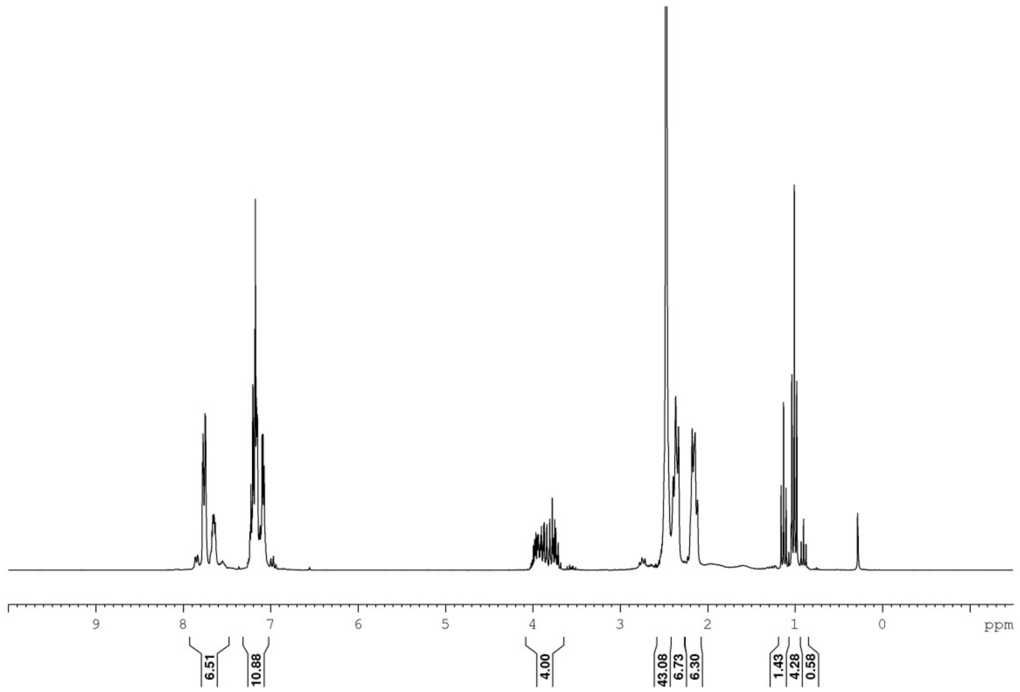

Figure S58: ^{1}H NMR spectrum of the reaction of **9b** with DABCO in C_6D_6 after 90 minutes at 50 °C.

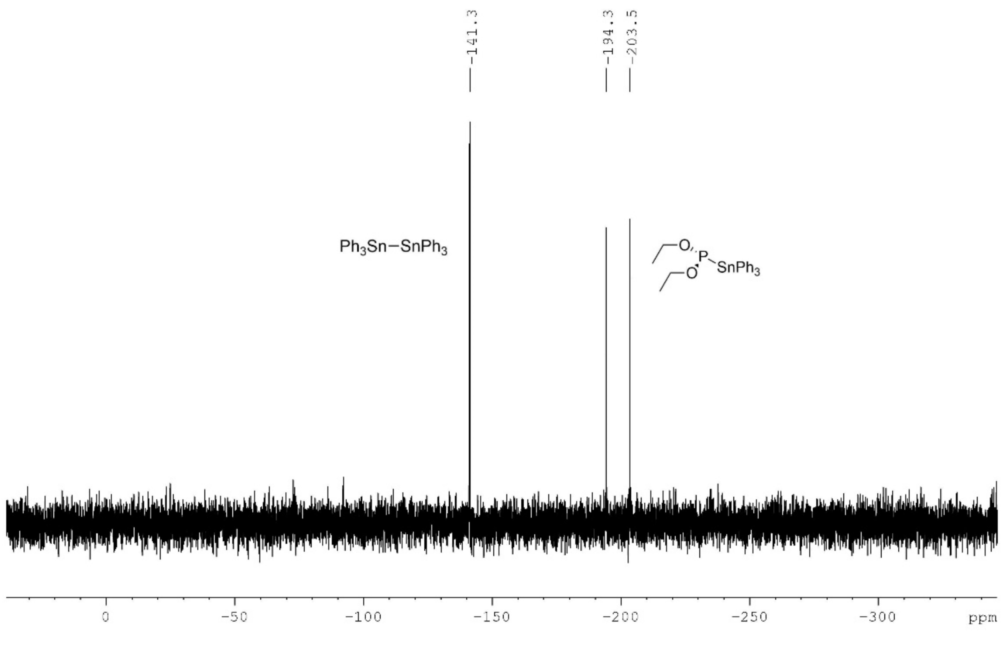

Figure S59: ^{119}Sn-DEPT spectrum of the reaction of **9b** with DABCO in C_6D_6 after 90 minutes at 50 °C.

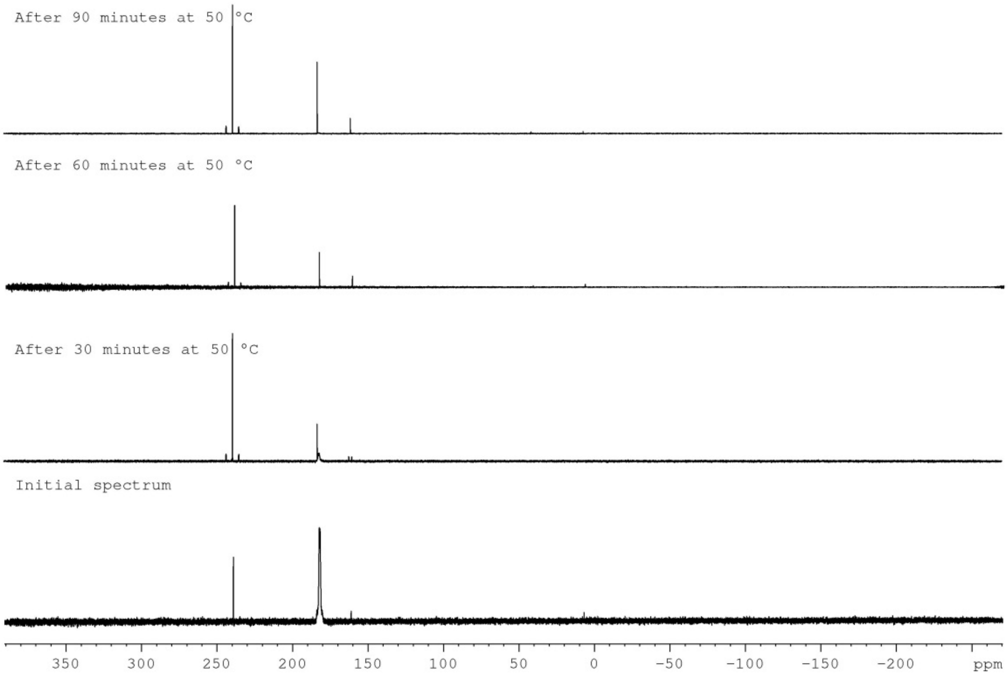

Figure S60: $^{31}P\{^{1}H\}$ NMR spectra of the reaction of **9b** with DABCO in C_6D_6 after 0, 30, 60, 90 minutes at 50 °C.

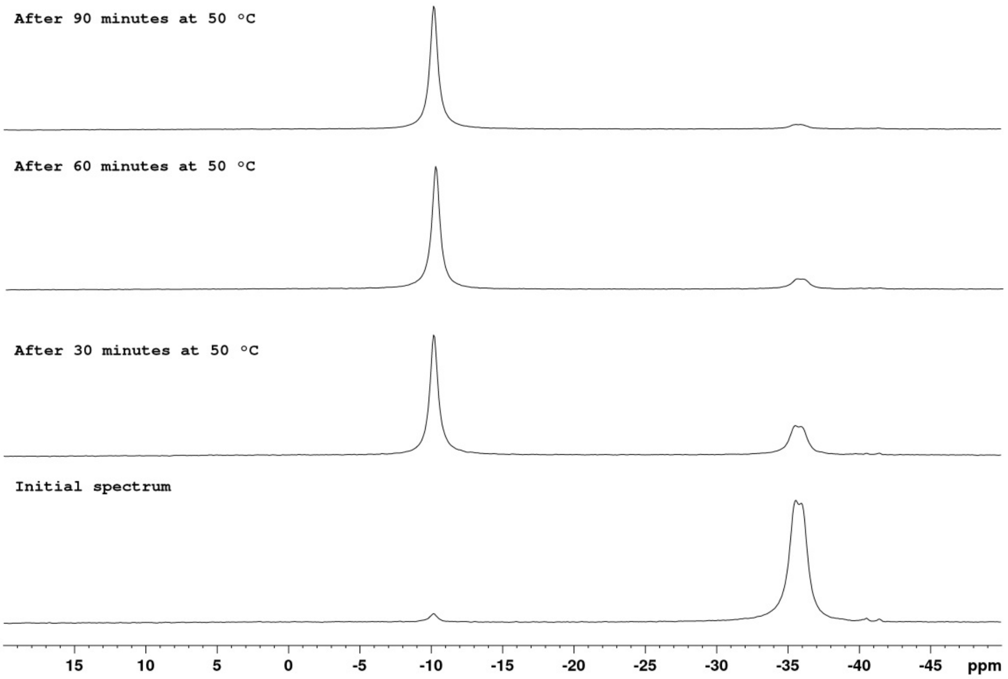

Figure S61: $^{11}B\{^{1}H\}$ NMR spectra of the reaction of **9b** with DABCO in C_6D_6 at 50°C after 0, 30, 60, 90 minutes at 50 °C

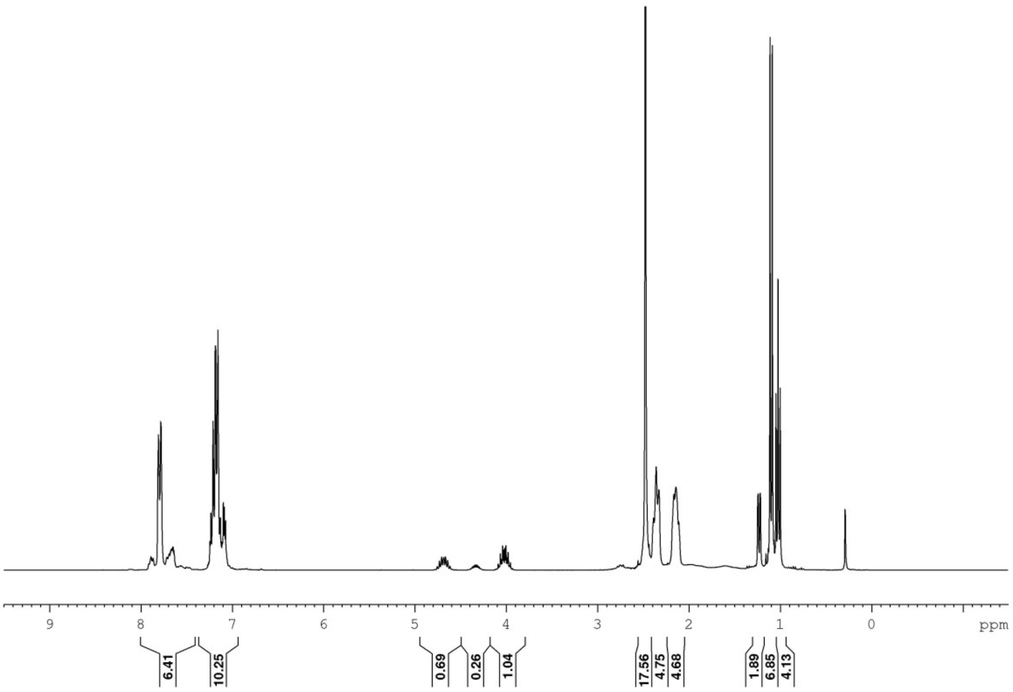

Figure S62: ^{1}H NMR spectrum of the reaction of **9c** with DABCO in C_6D_6 after 90 minutes at 50°C.

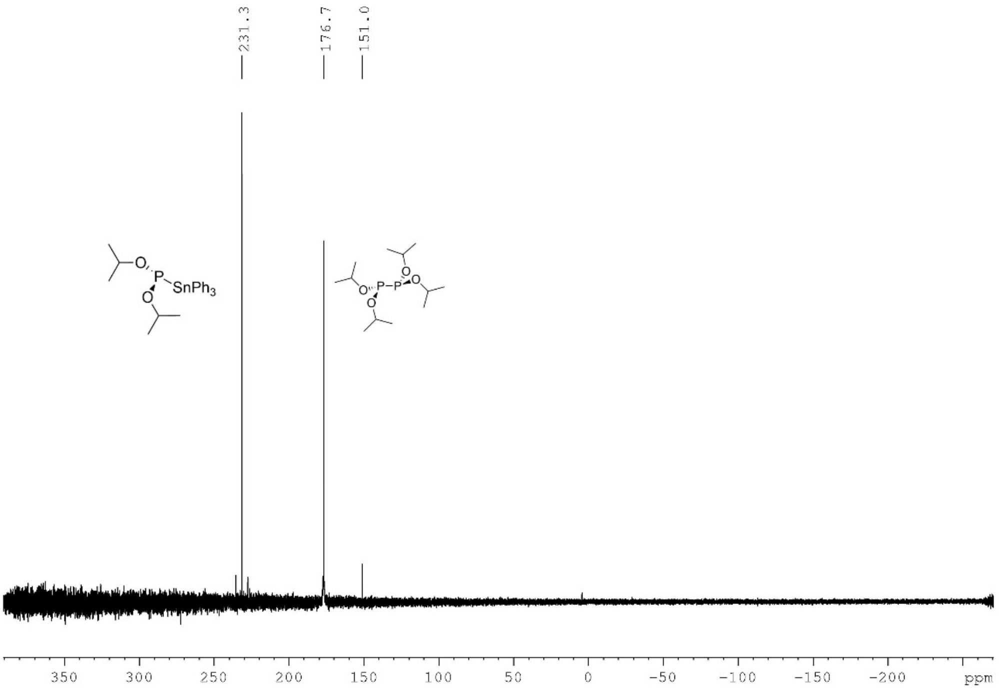

Figure S63: $^{31}P\{^1H\}$ NMR spectrum of the reaction of **9c** with DABCO in C_6D_6 after 90 minutes at 50°C.

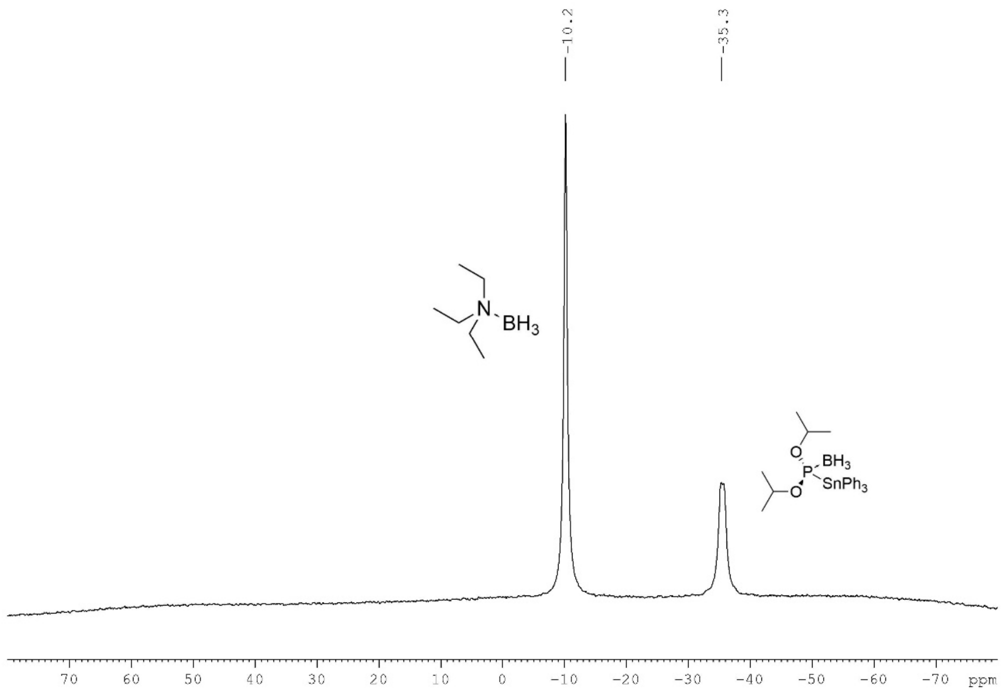

Figure S64: $^{11}B\{^{1}H\}$ NMR spectrum of the reaction of **9c** with DABCO in C_6D_6 after 90 minutes at 50°C.

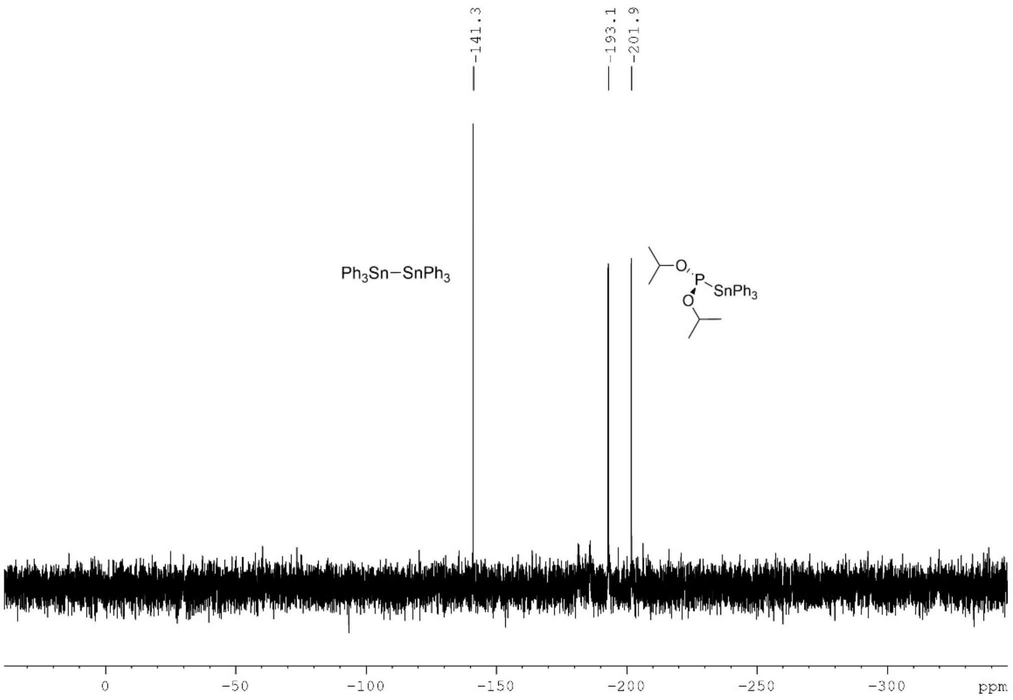

Figure S65: ^{119}Sn-DEPT NMR spectrum of the reaction of **9c** with DABCO in C_6D_6 after 90 minutes at 50°C.

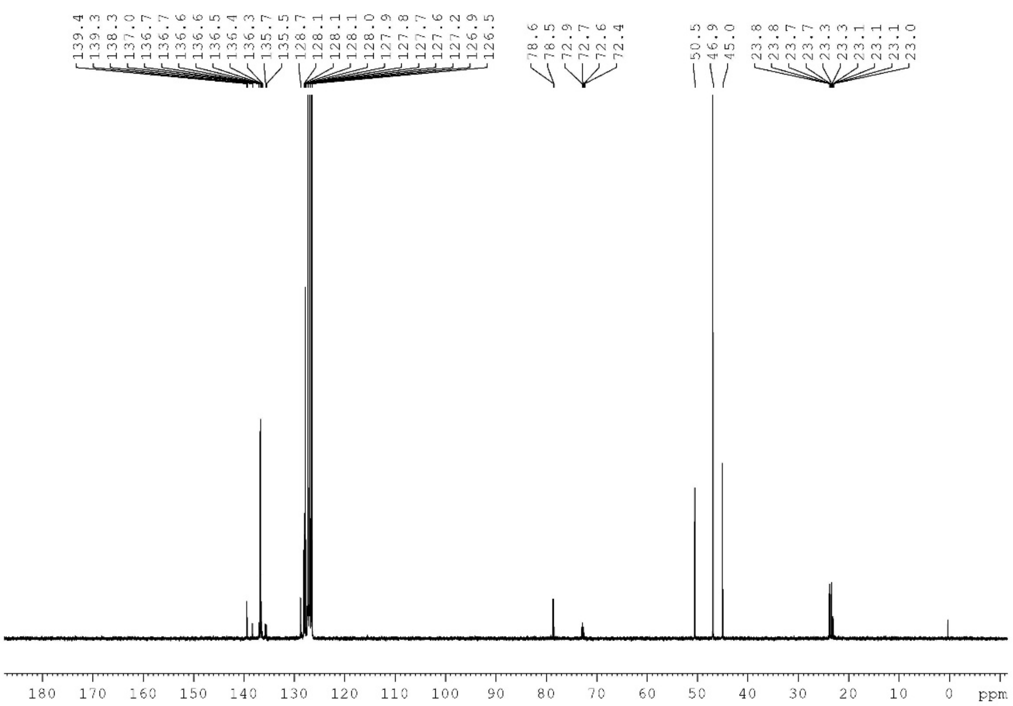

Figure S66: $^{13}C\{^{1}H\}$ NMR spectrum of the reaction of **9c** with DABCO in C_6D_6 after 90 minutes at 50°C.

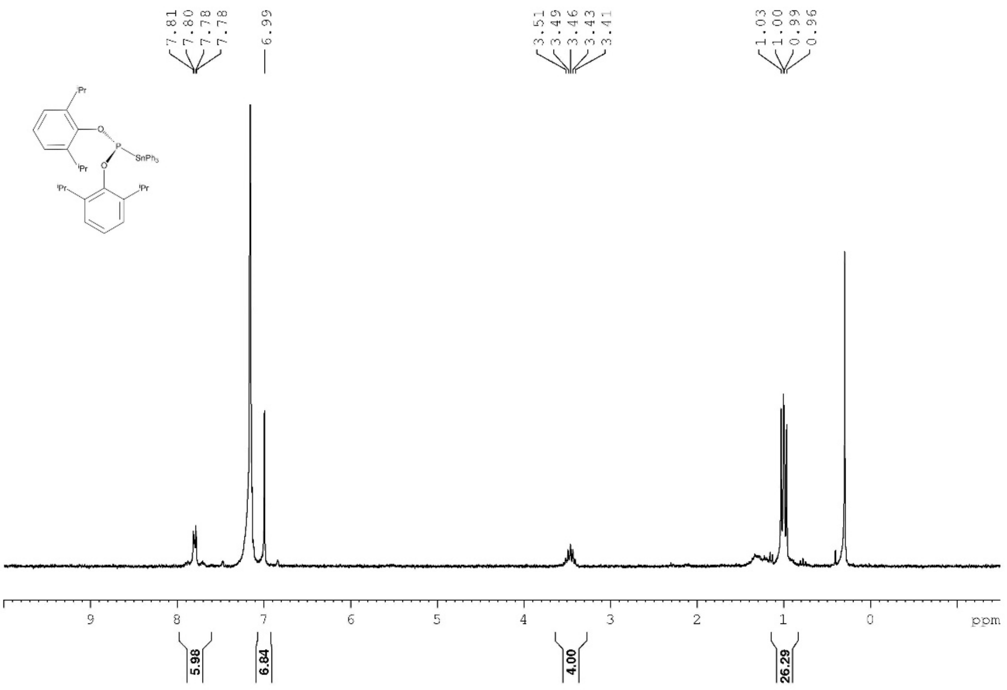

Figure S67: ^{1}H NMR spectrum of **10d** in C_6D_6.

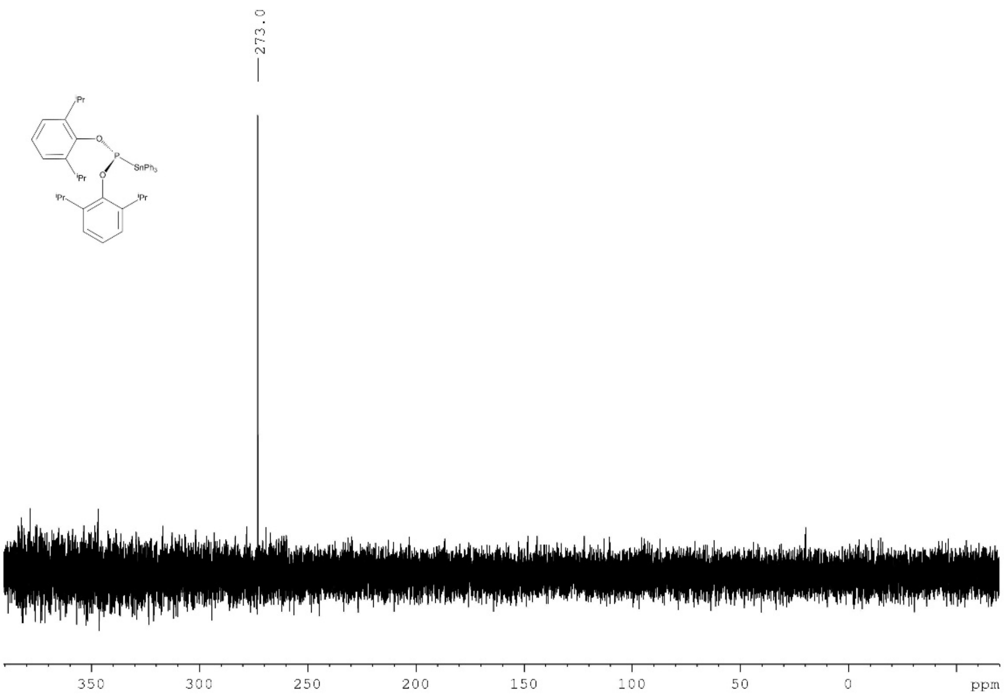

Figure S68: ^{31}P NMR spectrum of **10d** in C_6D_6.

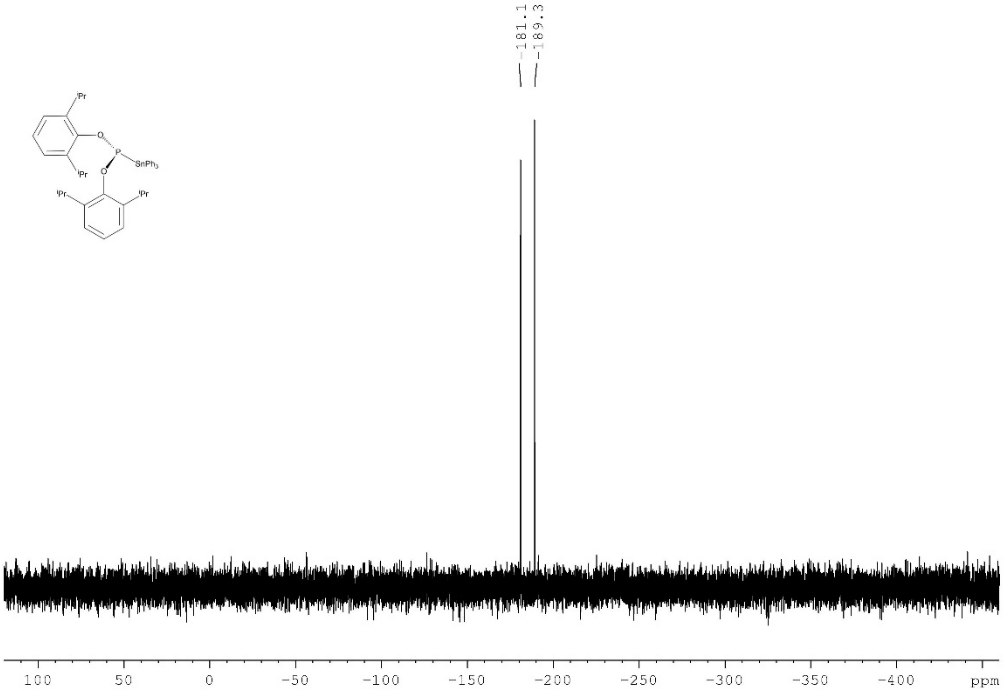

Figure S69: ^{119}Sn-DEPT spectrum of **10d** in C_6D_6.

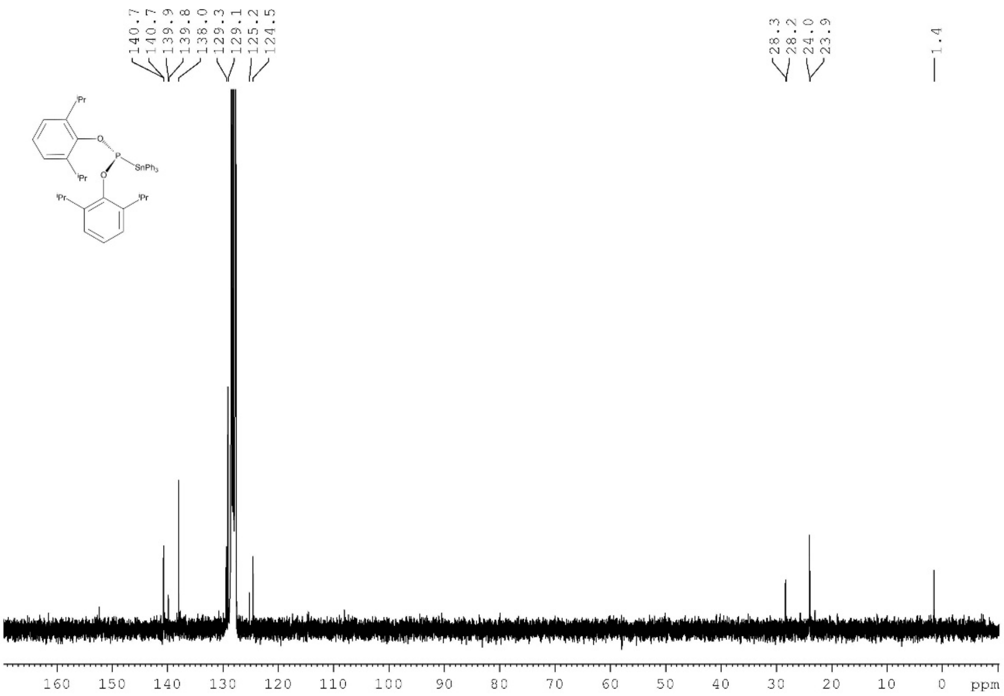

Figure S70: $^{13}C\{^{1}H\}$ NMR spectrum of **10d** in C_6D_6.

IR-Spectra

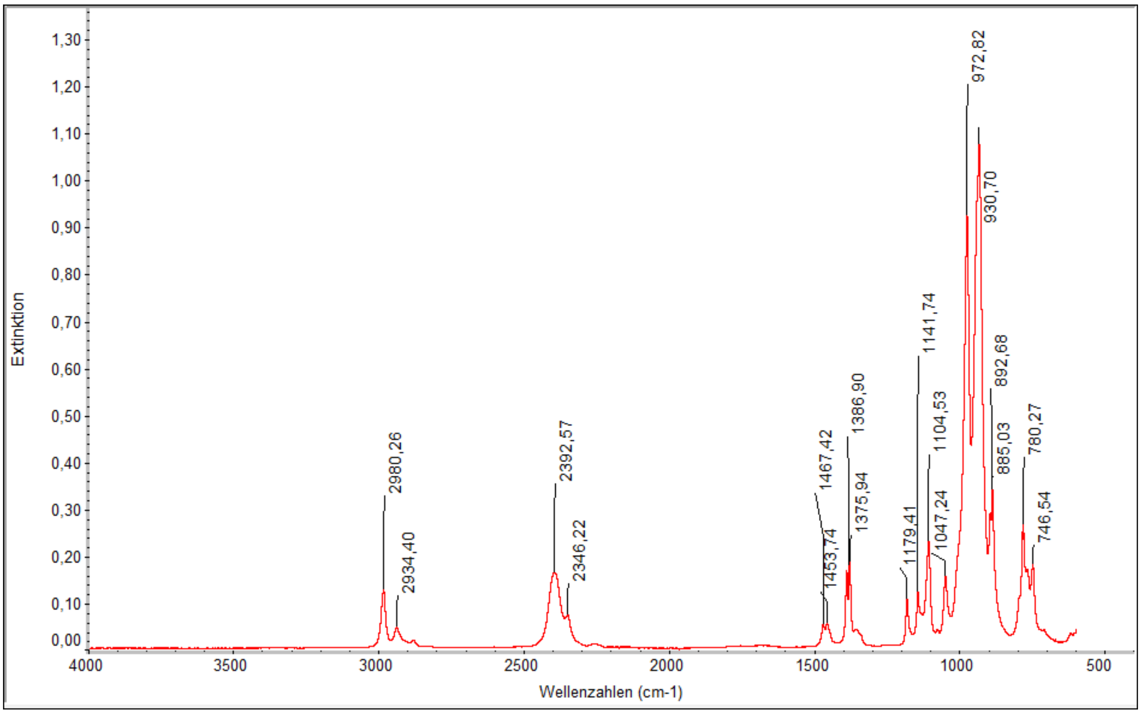

Figure S71: ATR-FTIR spectrum of **2c** (liquid, bulk).

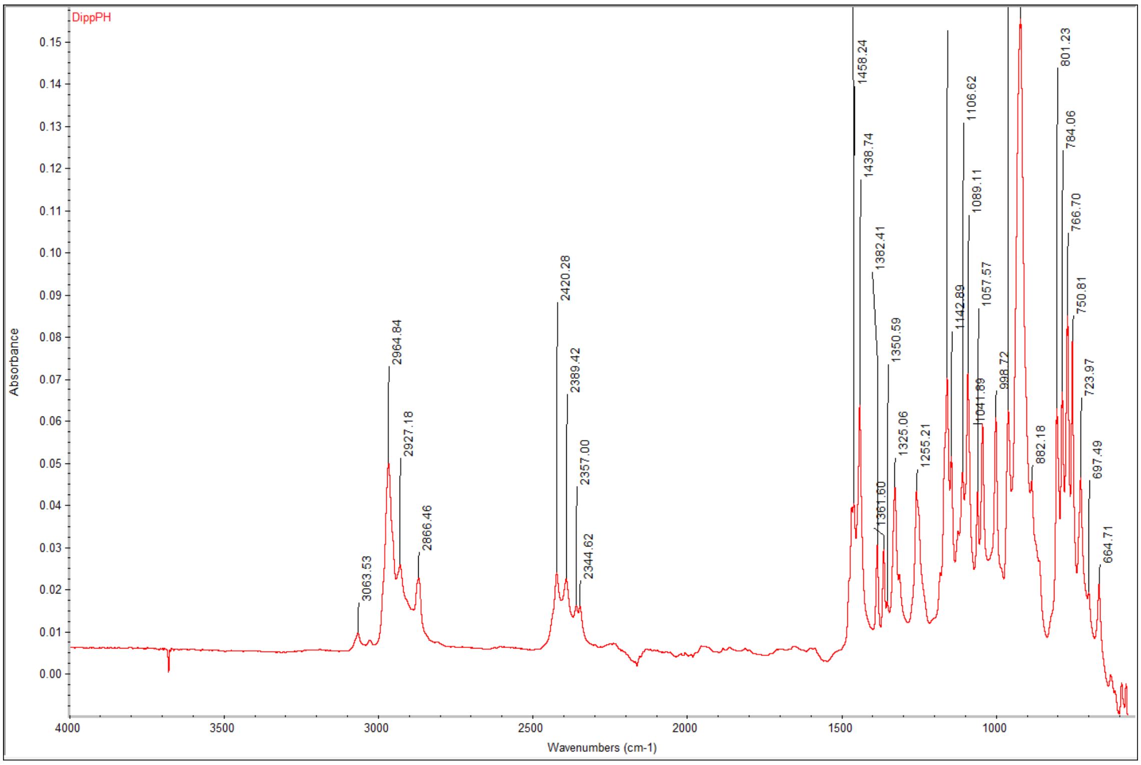

Figure S72 ATR-FTIR spectrum of **2d** (solid).

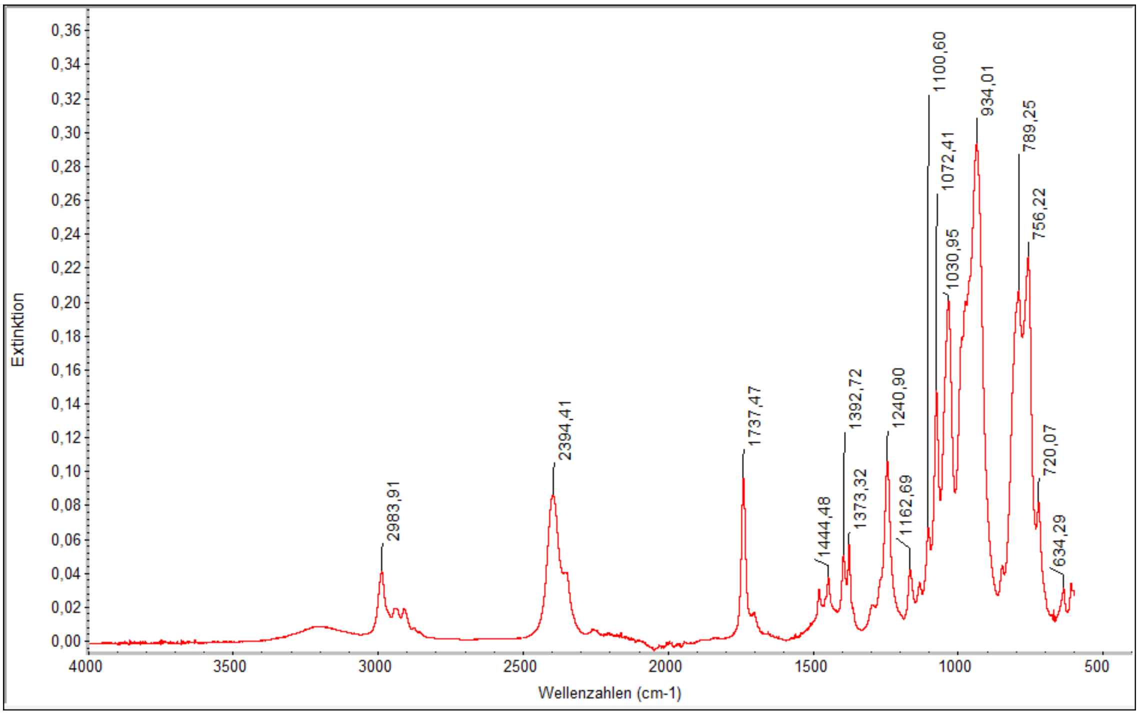

Figure S73 ATR-FTIR spectrum of K**[3b]** (solid).

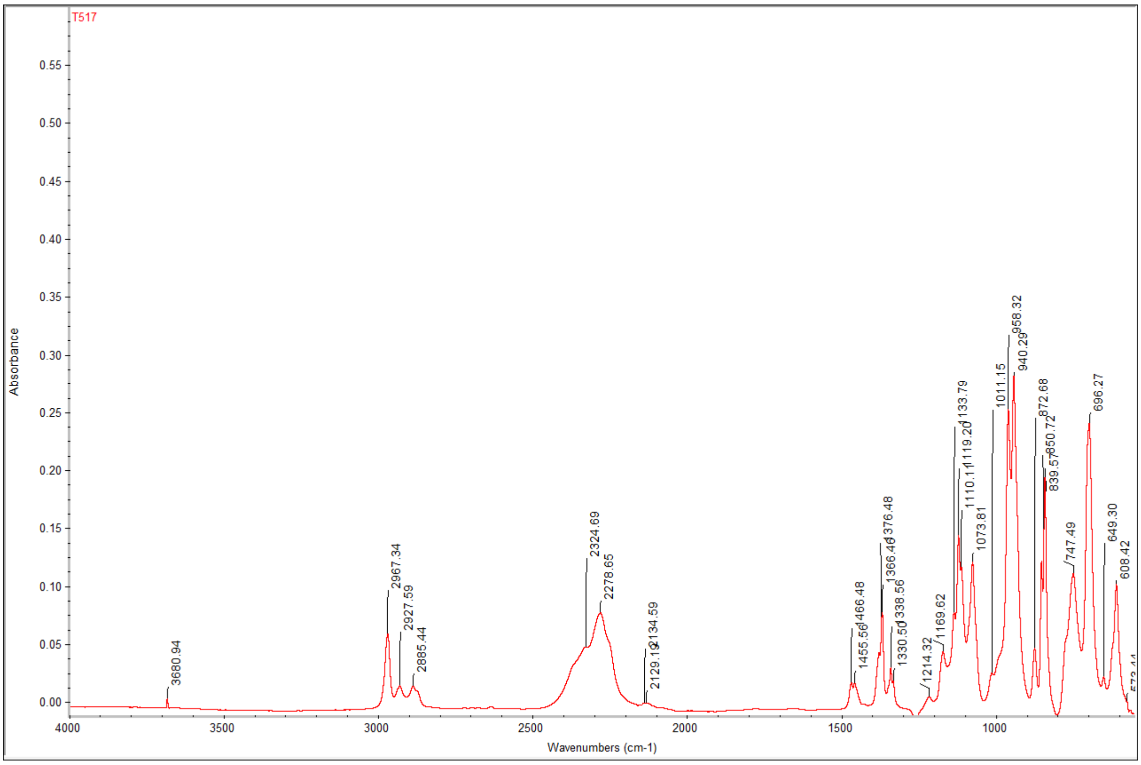

Figure S74 ATR-FTIR spectrum of K**[3c]** (solid).

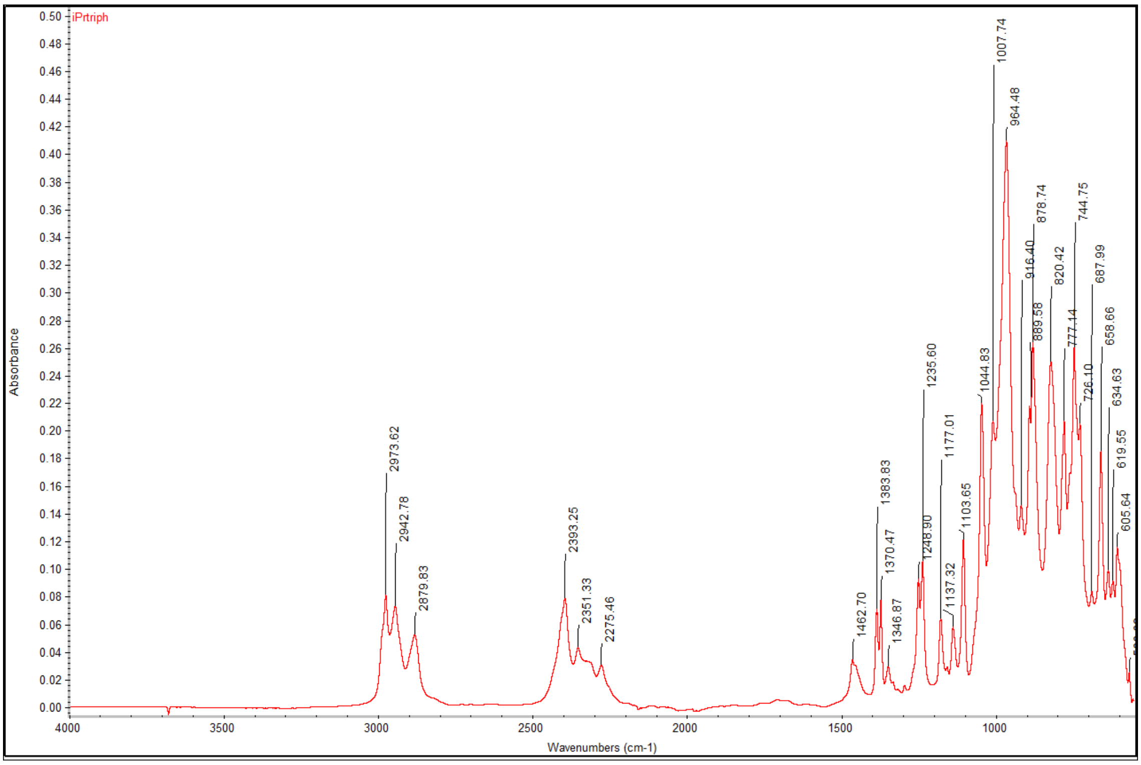

Figure S75: ATR-FTIR spectrum of Li**[5c]** (solid).

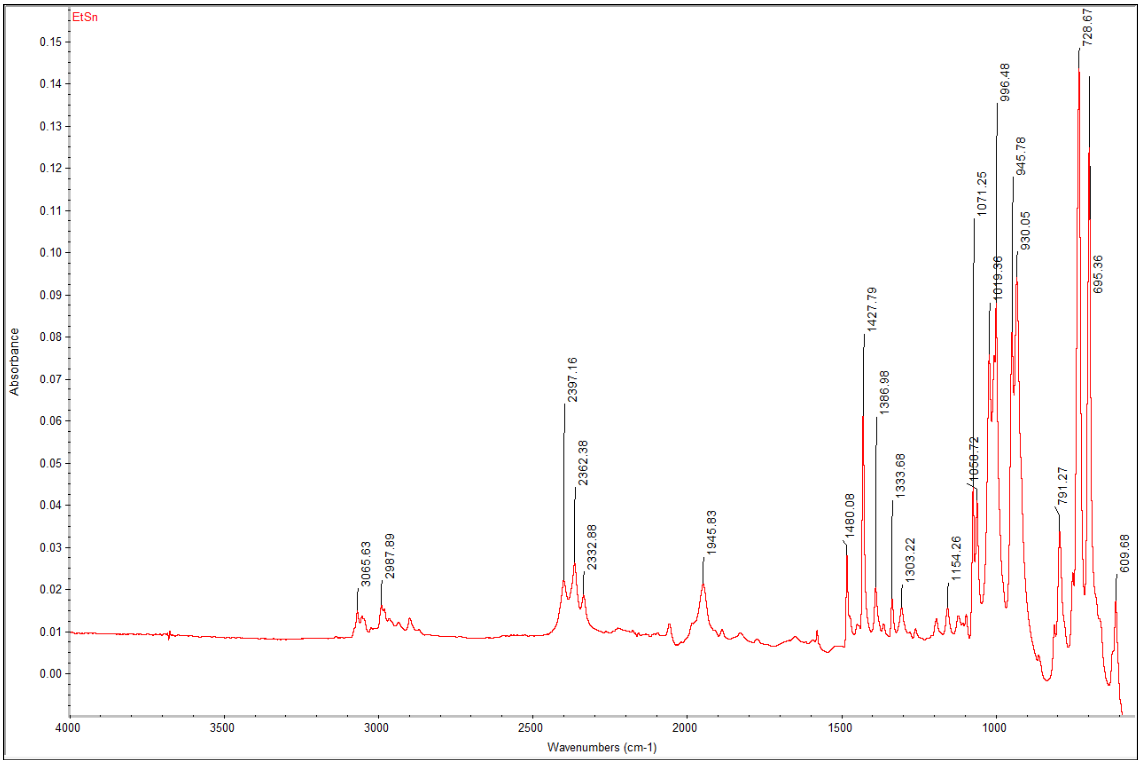

Figure S76: ATR-FTIR spectrum of **9b** (solid).

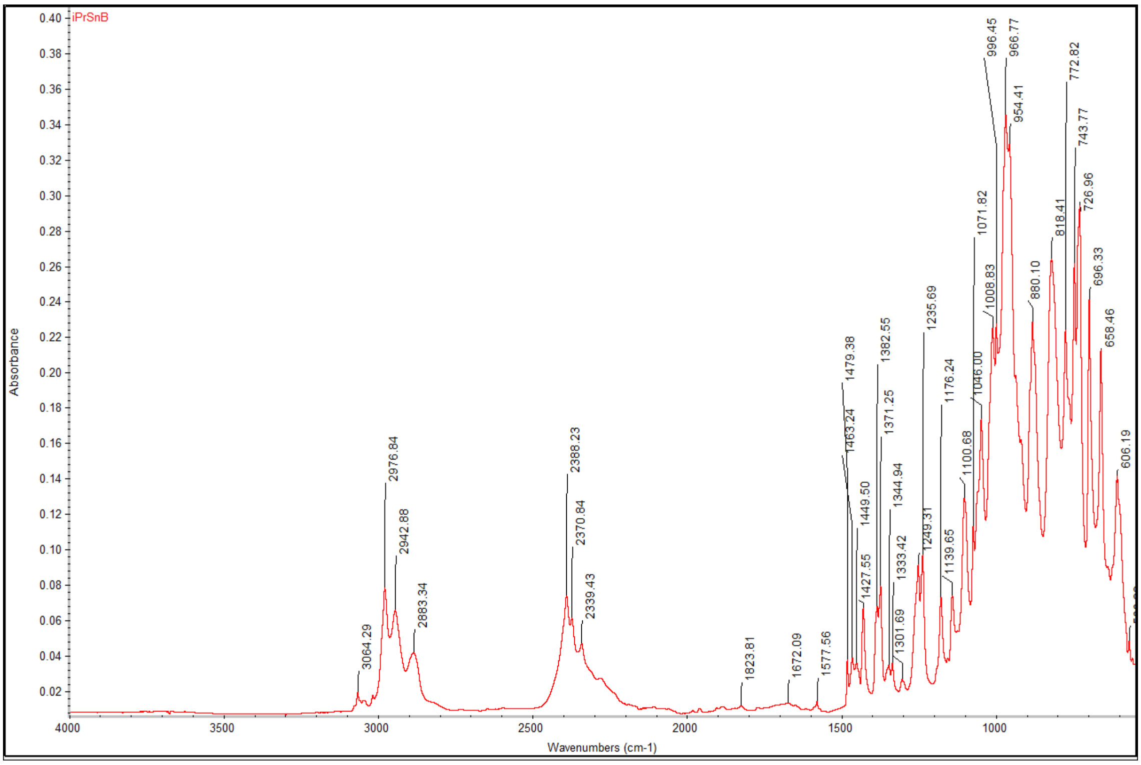

Figure S77: ATR-FTIR spectrum of **9c** (solid).

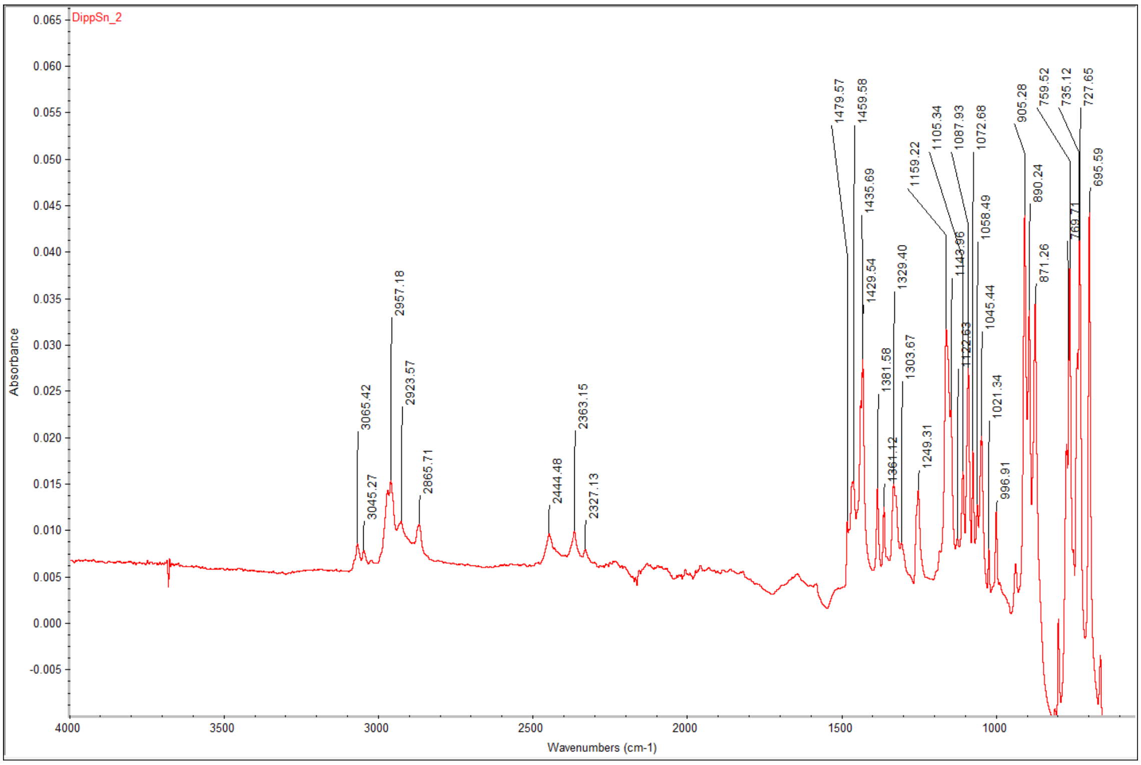

Figure S78: ATR-FTIR spectrum of **9d** (solid).

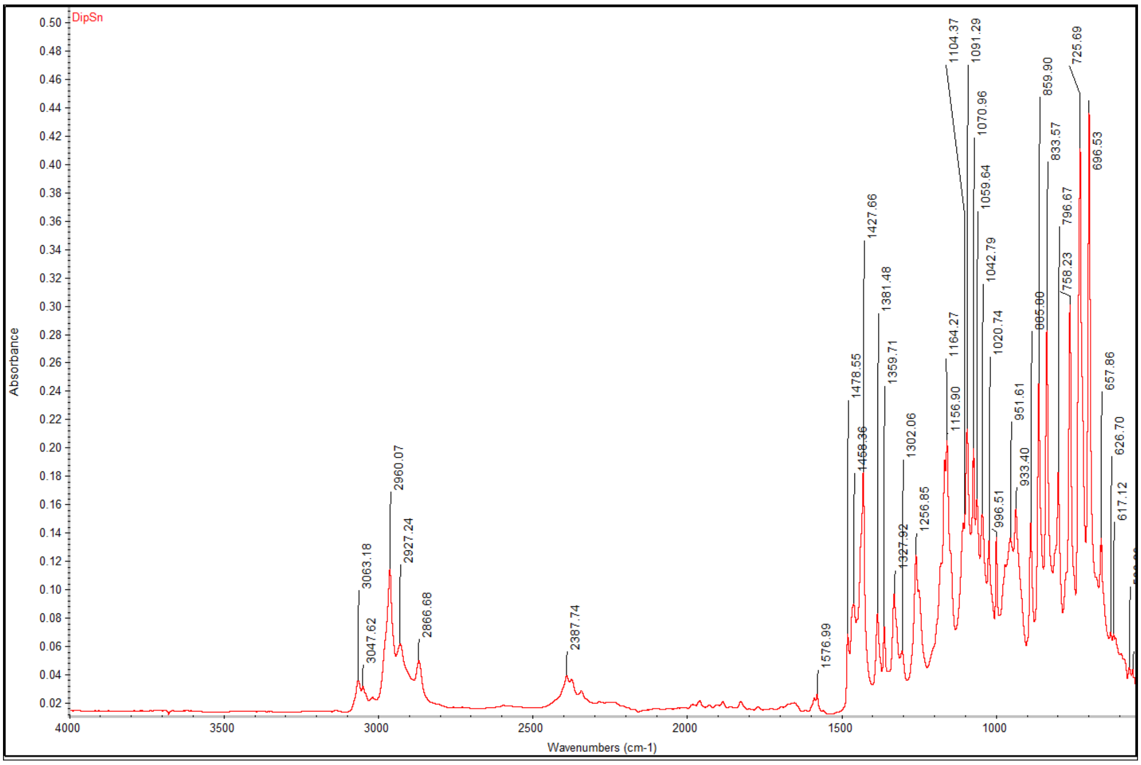

Figure S79: ATR-FTIR spectrum of **10d** (solid).

Mass Spectra

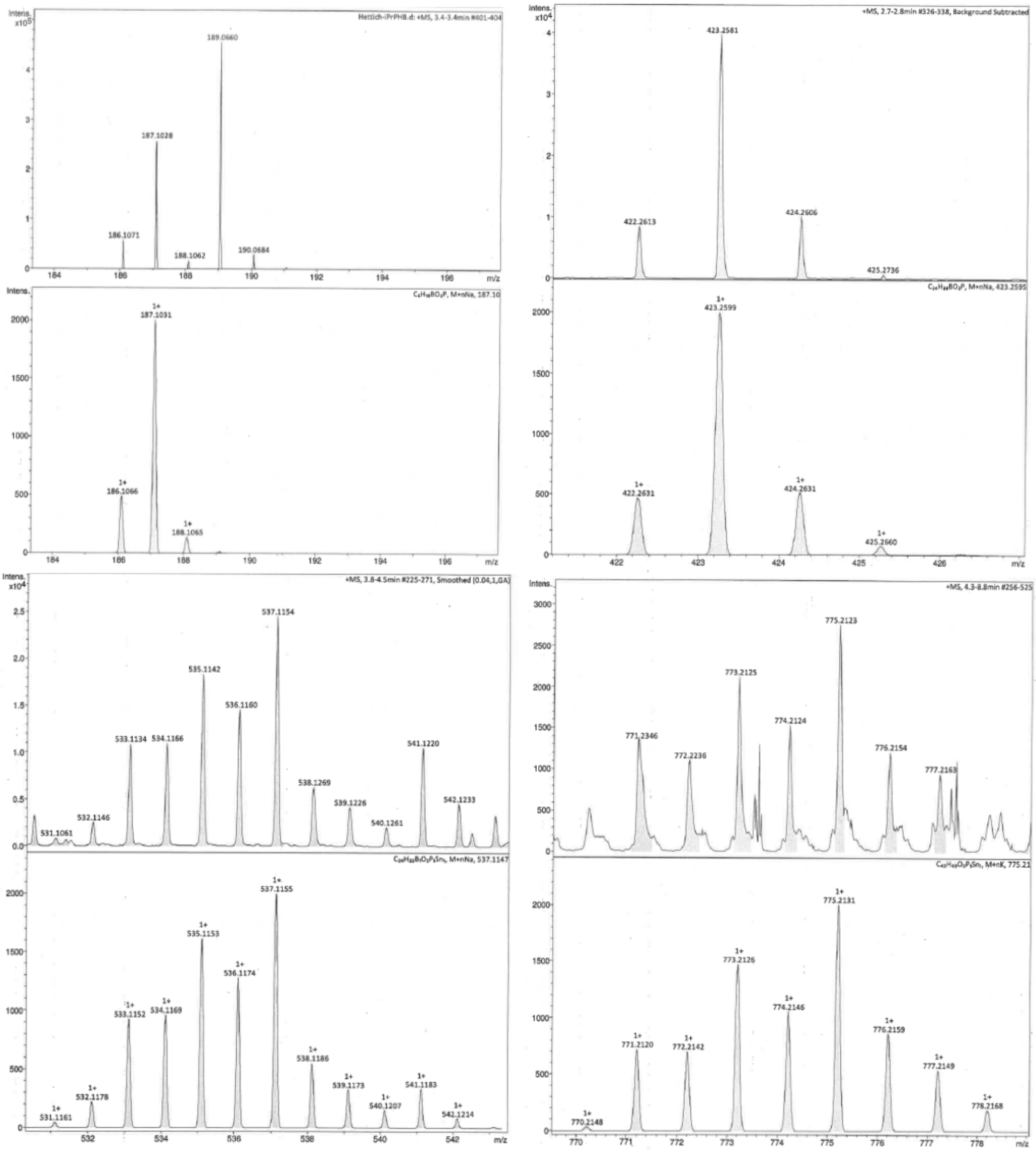

Figure S80: Observed and calculated isotopic patterns of the (pseudo)molecular ions in (+)-ESI mass spectra of **2c,d** (top row) and **9c**, **10d** (bottom row).

Computational Studies

General remarks. DFT calculations were performed with the Gaussian 16 program package[8] using the B3LYP functional,[9] which is an established standard in main group element chemistry, with basis sets from Weigend's and Ahlrichs' def2-family,[10] and application of the D3 version of Grimme's dispersion correction with Becke-Johnson damping[11] and the PCM formalism (keyword scrf, solvent=THF) to model solvation. The molecular structures were established by full energy optimization at the PCM-B3LYP-D3BJ/def2-svp level and identified as local minima on the potential energy hypersurface by subsequent harmonic vibrational frequency calculations. Magnetic shieldings were obtained from single point calculations at the PCM-B3LYP-D3BJ/def2-tzvpp level at the optimised geometries. NBO population analyses of electron densities were carried out using the NBO module implemented in the Gaussian package. The analysis of magnetic shieldings was carried out with NBO6.[12]

Chemical shifts were computed as $\delta_s = (\sigma_{ref} - \sigma_s - 266.1)$ relative to 85% H_3PO_4 [13] using the magnetic shielding constants of PH_3 (σ_{ref} = 590.5 ppm) calculated at the same computational level for referencing.

Table S2 Computed ^{31}P magnetic shielding parameters and HOMO-LUMO gaps for **2b**, **3b**$^-$, $HP(OEt)_2$, and $Me_3SnP(OEt)_2$ (**10b'**).

Compound	$\Delta E_{HOMO,LUMO}$/eV [a)]	δ^{31}P/ppm	$\sigma_{iso}(^{31}P)$	$\sigma_{dia}(^{31}P)$	$\sigma_{para}(^{31}P)$
**2b**	8.43	206.4	121.4	961.2	-891.0
**3b**$^-$	6.08	316.1	-86.7	965.2	-1004.7
$HP(OEt)_2$	7.16	155.2	70.2	960.3	-838.9
**10b'** [b)]	6.09	363.3	-39.5	960.6	-1047.3

[a)] calculated at the PCM-B3LYP-D3BJ/def2-tzvpp//PCM-B3LYP-D3BJ/def2-svp level of theory; [b)] $\sigma_{iso}(^{31}P)$ and δ^{31}P for **10b** were calculated as -39.3 and 315.9 ppm but the limitations of the available NBO module precluded further analysis. $\Delta E_{HOMO,LUMO+6}$ of **10b** (the LUMO+6 is in this case the lowest unoccupied MO with a significant local contribution at phosphorus) was computed as 5.98 eV.

Table S3 Natural orbital contributions (in ppm) to the ^{31}P paramagnetic shielding term in **2b**, **3b**$^-$, $HP(OEt)_2$, and $Me_3SnP(OEt)_2$ (**10b'**).

Compound	σ(P–O)		σ(P–B)	σ(P–H)	l.p.(P)
**2b**	-125	-123	-315	-210	--
**3b**$^-$	-103	-89	-398	--	-368
$HP(OEt)_2$	-111	-123	--	-231	-301
**10b'** [b)]	-123	-125	--	-272 [a)]	-337

[a)] σ(P–Sn) for **10b'**.

Table S4 Computed energies and Gibbs enthalpy corrections (in Hartree) and atomic coordinates (in Å) for **2b**, **3b**$^-$, $HP(OEt)_2$, and $Me_3SnP(OEt)_2$ (**10b'**).[a)]

	**3b**$^-$				**2b**		
E1	-677.224573073				-676.725397323		
ΔG-corr.	0.144201				0.133702		
E2	-677.754113127				-677.251550632		
	X	Y	Z		X	Y	Z
C	-2.372450	-1.521249	0.222454	C	-2.310589	-1.530575	0.077119
C	-2.478104	-0.039925	-0.086733	C	-2.449782	-0.012386	0.111701
O	-1.362260	0.704103	0.446437	O	-1.271940	0.629642	0.558328
P	0.016333	0.926960	-0.371180	P	-0.066203	1.007911	-0.598840
B	1.113519	2.206022	0.501344	B	1.081039	2.140946	0.499120
O	0.662237	-0.533951	-0.674567	O	0.718007	-0.501636	-0.757619
C	1.442157	-1.223113	0.331456	C	1.375950	-1.129329	0.332702
C	2.922404	-1.100121	0.037143	C	2.884264	-1.121302	0.138404
H	-0.413374	1.209651	-1.689062	H	2.087837	2.378928	-0.183024
H	2.079339	2.326940	-0.238595	H	0.432458	3.182624	0.668335
H	0.416370	3.207994	0.558198	H	1.384049	1.630197	1.581110
H	1.391612	1.739807	1.594908	H	-2.024256	-1.912702	1.070645
H	-2.255539	-1.682085	1.305052	H	-1.526660	-1.823038	-0.637080
H	-1.514517	-1.972577	-0.296080	H	-3.260860	-2.006647	-0.217584
H	-3.288643	-2.035633	-0.107051	H	-3.264081	0.275170	0.802442
H	-3.369786	0.400690	0.382013	H	-2.730933	0.356245	-0.896077
H	-2.548236	0.137129	-1.174471	H	3.264174	-0.088569	0.130386
H	3.249277	-0.051790	0.100818	H	3.152010	-1.593805	-0.821031
H	3.149134	-1.479196	-0.971082	H	3.389794	-1.673436	0.948802
H	3.499081	-1.689057	0.767560	H	1.007733	-2.171436	0.391186
H	1.110535	-2.271322	0.296578	H	1.109006	-0.639416	1.283653
H	1.198316	-0.829842	1.331305				

[a)] E1, ΔG-correction and atomic coordinates calculated at the PCM-B3LYP-D3BJ/def2-svp level of theory and E2 at the PCM-B3LYP-D3BJ/def2-tzvpp//PCM-B3LYP-D3BJ/def2-svp level of theory.

Table S4 (continued)

**10b'**				HP(OEt)$_2$			
E1	-984.020981972				-650.569328329		
ΔG-corr.	0.196403				0.115022		
E2	-984.664687646				-651.064124698		
	X	Y	Z		X	Y	Z
C	-2.493130	1.828993	-0.685015	C	-1.722146	1.768600	0.051215
Sn	-1.532947	0.027908	0.062831	C	-2.285109	0.361981	0.173559
C	-2.716395	-1.740659	-0.381305	O	-1.521920	-0.595370	-0.568411
P	0.780508	-0.197215	-0.993198	P	-0.197152	-1.374770	0.041584
O	1.316053	1.191800	-0.198419	O	0.880470	-0.168360	0.463360
C	2.526277	1.834061	-0.605599	C	1.810684	0.282177	-0.524863
C	3.324956	2.238582	0.619103	C	3.107230	0.677567	0.152950
C	-1.160793	0.187093	2.196146	H	-0.655455	-1.517554	1.397598
O	1.194488	-1.457682	0.050763	H	-1.636026	2.056774	-1.008266
C	2.344095	-2.257195	-0.234885	H	-0.726101	1.828255	0.512148
C	3.594920	-1.722527	0.440960	H	-2.388164	2.489171	0.552221
H	2.113813	-3.271614	0.129366	H	-3.308787	0.317474	-0.231829
H	2.494197	-2.322266	-1.329008	H	-2.331853	0.051396	1.234446
H	4.437419	-2.417271	0.294184	H	3.553669	-0.186515	0.669226
H	3.885600	-0.745497	0.025539	H	2.930083	1.470283	0.896496
H	3.427786	-1.600711	1.522410	H	3.828777	1.053957	-0.589135
H	3.115371	1.159862	-1.254952	H	1.372901	1.142192	-1.062445
H	2.269571	2.722788	-1.209316	H	1.985892	-0.515988	-1.270988
H	4.242807	2.770934	0.322456				
H	2.731059	2.905574	1.263185				
H	3.607592	1.354133	1.210142				
H	-2.106392	0.308358	2.744978				
H	-0.648486	-0.722170	2.540797				
H	-0.512306	1.054935	2.381690				
H	-1.803323	2.679117	-0.580572				
H	-2.756952	1.707019	-1.745734				
H	-3.407254	2.033649	-0.107882				
H	-2.141306	-2.640694	-0.119257				
H	-3.647191	-1.724724	0.205185				
H	-2.966180	-1.771616	-1.451942				

References

[1] S.-B. Chen, Y.-M. Li, S.-Z. Luo, G. Zhao, B. Tan, Y.-F. Zhao, *Phosphorus, Sulfur, Silicon Rel. Elem.* **2000**, *164*, 277–291.

[2] D. J. Collins, P. F. Drygala, J. M. Swan, *Aust. J. Chem.* **1983**, *36*, 2517–36.

[3] K. N. Gavrilov, V. N. Tsarev, M. G. Maksimova, O. G. Bondarev, E. A. Rastorguev, S. E. Lyubimov, P. V. Petrovskii, V. A. Davankov, *J. Molec. Catal. A* **2006**, *259*, 267–274.

[4] Y. Belabassi, M. I. Antczak, J. Tellez, J.-L. Montchamp, *Tetrahedron* **2008**, *64*, 9181–9190.

[5] R. H. Harris, E. D. Becher, S. M. Cabral de Menezes, R. Goodfellow, P. Granger, *Pure Appl.* Chem., 2001, **73**, 1795–1818.

[6] G. Baccolini, C. Boga, M. Mazzacurati, F. Sangirardi, *Org. Lett.* **2006**, *8*, 1677–1680.

[7] G. M. Sheldrick, *Acta Cryst.* **2015**, *C71*, 3-8; b) G. M. Sheldrick, Acta Cryst. **2008**, *A64*, 112-122.

[8] Gaussian 16, Revision C.01, M. J. Frisch, G. W. Trucks, H. B. Schlegel, G. E. Scuseria, M. A. Robb, J. R. Cheeseman, G. Scalmani, V. Barone, G. A. Petersson, H. Nakatsuji, X. Li, M. Caricato, A. V. Marenich, J. Bloino, B. G. Janesko, R. Gomperts, B. Mennucci, H. P. Hratchian, J. V. Ortiz, A. F. Izmaylov, J. L. Sonnenberg, D. Williams□Young, F. Ding, F. Lipparini, F. Egidi, J. Goings, B. Peng, A. Petrone, T. Henderson, D. Ranasinghe, V. G. Zakrzewski, J. Gao, N. Rega, G. Zheng, W. Liang, M. Hada, M. Ehara, K. Toyota, R. Fukuda, J. Hasegawa, M. Ishida, T. Nakajima, Y. Honda, O. Kitao, H. Nakai, T. Vreven, K. Throssell, J. A. Montgomery, Jr., J. E. Peralta, F. Ogliaro, M. J. Bearpark, J. J. Heyd, E. N. Brothers, K. N. Kudin, V. N. Staroverov, T. A. Keith, R. Kobayashi, J. Normand, K. Raghavachari, A. P. Rendell, J. C. Burant, S. S. Iyengar, J. Tomasi, M. Cossi, J. M. Millam, M. Klene, C. Adamo, R. Cammi, J. W. Ochterski, R. L. Martin, K. Morokuma, O. Farkas, J. B. Foresman, and D. J. Fox, Gaussian, Inc., Wallingford CT, 2016.

[9] A. D. Becke, *J. Chem. Phys.* **1993**, *98*, 5648–5652.

[10] F. Weigend R. Ahlrichs, *Phys. Chem. Chem. Phys.* ***2005***, *7*, 3297-3305.

[11] S. Grimme, S. Ehrlich, L. Goerigk, *J. Comp. Chem.* **2011**, *32*, 1456–1465.

[12] NBO 6.0. E. D. Glendening, J. K. Badenhoop, A. E. Reed, J. E. Carpenter, J. A. Bohmann, C. M. Morales, C. R. Landis, F. Weinhold (Theoretical Chemistry Institute, University of Wisconsin, Madison, WI, 2013); http://nbo6.chem.wisc.edu/

[13] C. van Wüllen, *Phys. Chem. Chem. Phys.* **2000**, *2*, 2137–2144.
